# Methods in DNA methylation array dataset analysis: A review

**DOI:** 10.1016/j.csbj.2024.05.015

**Published:** 2024-05-17

**Authors:** Karishma Sahoo, Vino Sundararajan

**Affiliations:** Integrative Multiomics Lab, School of Bio Sciences and Technology, Vellore Institute of Technology, Vellore, Tamil Nadu 632014, India

**Keywords:** DMR analysis, Biomarker identification, Prognostic models, Clustering, Molecular subtyping, Methylation segmentation

## Abstract

Understanding the intricate relationships between gene expression levels and epigenetic modifications in a genome is crucial to comprehending the pathogenic mechanisms of many diseases. With the advancement of DNA Methylome Profiling techniques, the emphasis on identifying Differentially Methylated Regions (DMRs/DMGs) has become crucial for biomarker discovery, offering new insights into the etiology of illnesses. This review surveys the current state of computational tools/algorithms for the analysis of microarray-based DNA methylation profiling datasets, focusing on key concepts underlying the diagnostic/prognostic CpG site extraction. It addresses methodological frameworks, algorithms, and pipelines employed by various authors, serving as a roadmap to address challenges and understand changing trends in the methodologies for analyzing array-based DNA methylation profiling datasets derived from diseased genomes. Additionally, it highlights the importance of integrating gene expression and methylation datasets for accurate biomarker identification, explores prognostic prediction models, and discusses molecular subtyping for disease classification. The review also emphasizes the contributions of machine learning, neural networks, and data mining to enhance diagnostic workflow development, thereby improving accuracy, precision, and robustness.

## Highlights

• Aberrant methylation sites can function as biomarkers and are applicable for the diagnosis of diseases.
• Several global repositories show easy availability of methylation to begin the analysis of the DNA methylation datasets.
• Clustering methods are extensively applied to group DNA methylation data into meaningful categories.
• Utilization of DMR analysis strategies and algorithms proves to be effective in identifying aberrant methylation sites.
• Annotation offers a thorough biological perspective of the results derived from the analysis.

## Graphical Abstract

**Figure:**
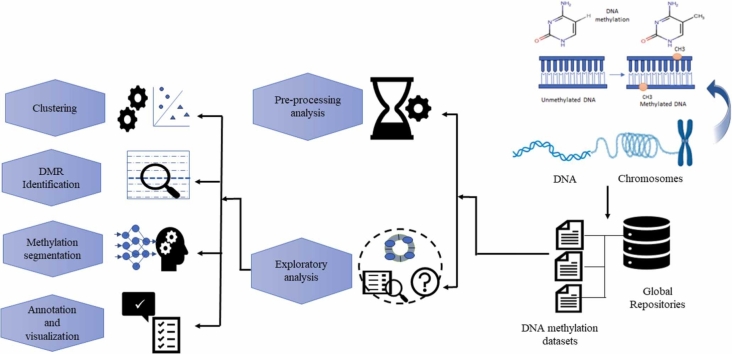

## 1 Introduction

DNA methylation has a long evolutionary history and can be found in all kingdoms of life, including eukaryotic and archaebacterial organisms, is extensively reported as a significant process for embryonic development and cellular function. This process adds a methyl group to the cytosines region of the CpG sites in the genome and is closely involved in the regulation of gene expression [1], [2]. DNA methylation is a crucial epigenetic modification of the genome important for cellular reprogramming, tissue differentiation, and proper development connected to many biological processes, including the control of gene expression [3], [4]. It is known that CpG dinucleotides, which are primarily located in so-called CGI (CpG islands) areas, undergo DNA methylation at the 5′ of the cytosine. Approximately 70% of gene promoters reside within the CpG islands, majorly including the promoters of the housekeeping genes [5]. DNA methylation at CpG islands (CGIs) regulates gene activity, playing a crucial role in gene silencing through promoter methylation, and can contribute to the pathogenesis of diseases [6], [7].

Various forms of cancer such as colon, breast, liver, bladder, oesophageal, prostate, and bone cancers, have been reported with aberrant DNA methylation of imprinted sites [8]. Multiple omics-based research studies have also revealed that a variety of malignancies, including hepatocellular carcinoma [9], glioblastoma, breast cancer, squamous cell lung cancer, thyroid carcinoma, and leukemia [10], have diverse DNA methylation patterns. Moreover, DNMT mutations, various DNMT expression levels, dysregulation of TETs, and frequent observations of cancer, point towards a strong association between DNA methylation and cancer [11].

Various experimental techniques are employed for the detection of DNA methylation in the genomic DNA. These include targeted bisulfite sequencing with TruSeq Methyl Capture, whole genome bisulfite sequencing, methylated DNA immunoprecipitation (MeDIP), pyrosequencing, Illumina Infinium DNA methylation, Nanopore DNA sequencing, and ultra-high performance liquid chromatography merged with mass spectrometry (UHPLC-MS/MS)[12], [13]. TruSeq EPIC sequencing offers targeted coverage of 3.34 million CpG sites, surpassing EPIC-array capabilities [14]. MeDIP-seq extends coverage to approximately 10% of the genome, with RRBS notably covering 85% of CGIs, primarily in promoter regions [15]. WGBS provides comprehensive genome coverage but is resource-intensive [16]. Pyrosequencing offers targeted analysis, while Illumina Infinium assays offer high-resolution single-CpG-site measurements. The data file sizes for methylation beta values typically range from 20 GB (unzipped) to 4 GB (zipped). Costs vary, with sequencing services such as WGBS and RRBS priced at around $300 per sample, while methylation array expenses average $425 per chip, covering reagents and labor costs for multiple samples. Further, the cost is also dependent upon the company providing the platform services. The scalability of the methods can range from moderate (done for multiple samples) to high (done for large amounts of samples). Many bioinformatics methods and pipelines, including Bigmelon [17], EpiScanpy [18], EpiMOLAS [19], MADA [20], AmpliconDesign [21], COHCAP [22], Bicycle [23], and ChAMP [24], have been developed for analyzing the extent of high throughput methylation dataset produced by the various platforms for conducting epigenome-wide association studies, whose output is in the proclaimed repositories.

Differentially methylated regions (DMRs), which are genomic areas that exhibit noticeable variations in levels of methylation between various biological states (e.g., normal versus diseased), have been discovered to be connected to several diseases [25]. Hence, one of the most important problems in understanding the mechanism of the disease at the molecular level is the detection of DMRs. Even though DNA methylation patterns arse stable throughout the cell growth mechanism of normal somatic cells, variation seen in genomic methylation might be caused by genetic differences or vice versa. However, whether methylation alteration is a cause or an effect is typically overlooked in traditional DMR analysis. For differential methylation analyses at the cell-type level, Rahmani et al., 2019 investigate the impact of model directionality and also state whether the methylation can affect the condition of interest (phenotype) or vice versa [26]. They demonstrate that identifying cell type-specific differential methylation depends significantly on properly accounting for model directionality.

The connections between methylation modifications and copy number variations (CNVs) provide a wider, and therefore more useful picture of the samples under analysis, particularly for tumor data defined by significant genomic rearrangements, according to current research [27]. As a result, the ability to measure CNVs using DNA methylation data is possible with recent developments in technology. One of the primary advantages of DNA methylation-based CNV approaches is their ability to incorporate epigenomic (methylation) information and genomic (copy number) information. In 2022, Mariani and colleagues introduced MethylMasteR, an R software package that incorporates CNV calling algorithms based on DNA methylation, making it easier to standardize, compare, and customize CNV investigations [28]. MethylMasteR enables performance evaluation, comparing runtime and memory usage, and assessing the detection of large-scale CNVs in cancer samples using four well-known methylation-based CNV algorithms: ChAMP [24], SeSAMe [29], Epicopy [30], and a modified version of cnAnalysis450k [31].

**Fig. 1:**
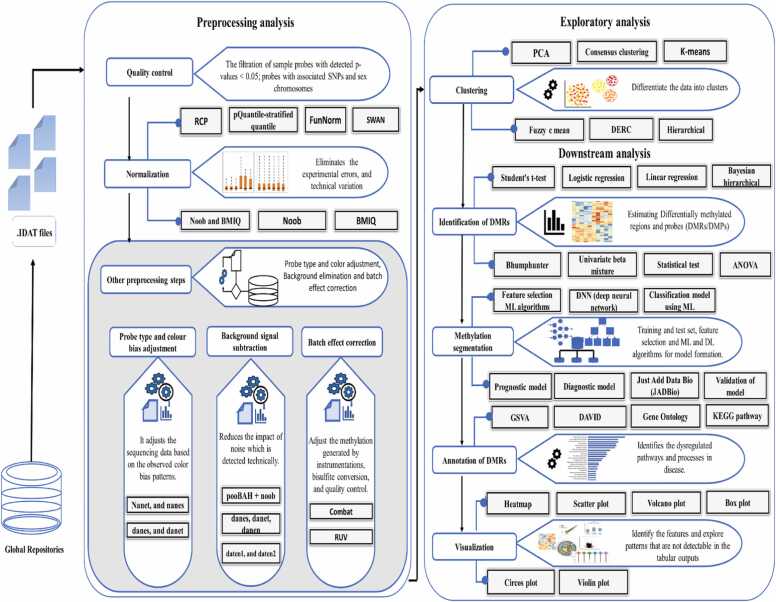
Visual representation of the step-wise analysis of methylation data with its significance and majorly used algorithms in the reviewed manuscripts. Depending upon the researcher objective, some manuscript demonstrates the application of all the steps within the single paper, while others showcase the application of a few selective steps.

A DNA methylation dataset encompasses the chromosome number, UCSC reference genome information, the chromosomal coordinates of the CpG island, experimentally determined differentially methylated regions with the specification of disease, regulatory feature details, and Hidden Markov Model Islands providing the details of the computationally predicted disease. The metadata describes the technical details of the DNA methylation profiling experiment, including the sequencing platform used, the title, repositories, and term accession, as well as a summary of information about the experimental conditions, sample preparation details (for control and case-defined groups), and experimental conditions [32]. The size of raw methylation array data files is dependent on the number of samples, which ranges from > 100 MB (with the inclusion of one sample) to 5–10 GB (maximum of 987 patient samples), accessible at the NCBI GEO database[33]. A data storage and retrieval system/platform, reference genome databases like UCSC Genome Browser [34] or ENCODE [35], bioinformatics tools like R studio, Anaconda, and Bioconductor packages, along with statistical tools R, SAS, or SPSS [36] are all necessary to work with large-scale data files from repositories. To process, interpret, and analyze the methylation array data for finding DMRs, computational methods are combined with statistical and data visualization techniques. This is followed by the use of tools for enrichment analysis necessary for the biological interpretation of the expressed methylation. Fig. 1 illustrates the systematic process, starting with data collection from public repositories, followed by the application of significant computational algorithms to analyze methylation datasets, in line with multiple predefined hypotheses delineated by the researchers.

Given the current worldwide emphasis on integrated disease management, it is impossible to overlook the emergence of novel computational paradigms available for fundamental and biomedical investigations. These approaches are crucial in examining their potential to significantly improve disease diagnosis capabilities. Despite the widespread usage of numerous methylation profiling approaches, the individual analytical processes of each go beyond the scope of this review and are therefore not discussed. These techniques include Whole Genome Bisulfite Sequencing (WGBS), Reduced Representation Bisulfite Sequencing (RRBS), MeDIP (Methylated DNA Immunoprecipitation), single-cell RRBS (scRRBS), and Next generation sequencing (NGS) [37], [38]. Technically, RRBS-seq, scRRBS, MeDIP, BS-Seq, and WGBS have established protocols for detecting methylated cytosines in genomic DNA. In contrast, Next-Generation Sequencing (NGS) provides a comprehensive view of nucleotide sequences across entire genomes or specific DNA/RNA regions. Existing literature reviews have thoroughly explored both the experimental profiling techniques related to DNA methylation and the computational methods employed for the analysis of DNA methylation data (array-based and sequence-based data) [15], [39], [40]. WGBS, RRBS, and NGS are the high throughput sequencing methods providing data whose analysis pipeline contains library preparation, alignment, quality control, methylation calling, and annotation [41], [42]. Similarly, MeDIP and MethylCap-seq data analysis also follow these post-sequencing steps [43], [44]. Also, methylation serves as a potent marker for distinguishing cells under varying conditions or cell types, pushing the boundaries of research into single-cell DNA methylation profiling whose analysis method is similar to that of bulk methylation data analysis [45]. But, as these technologies are promising, DNA hybridization microarrays are increasingly utilized for their cost-effectiveness, rapid analysis, and broad coverage using a predetermined set of CpG sites. This technology also supports a wide range of experiments, encompassing genotyping, epigenetics, translation profiling, and gene expression analysis. Notably, the Illumina Infinium HumanMethylation BeadChip array is a widely used high-throughput option, providing the most comprehensive genome-wide DNA methylation data available in the GEO database for disease research [46]. Therefore, this review highlights studies that utilize DNA Infinium microarray data to effectively identify methylation markers for disease. The article selection focuses exclusively on identifying methylated regions/sites as diagnostic or prognostic biomarkers for human diseases, supplemented by analyses/methods that enhance their biological relevance. We compiled the articles by searching the web, PubMed, and Google Scholar with the keywords "DNA methylation array analysis in cancer", “DNA methylation array analysis in diseases” AND "Computational methods for analyzing DNA methylation array," along with the filters (selected parameters) used for the collection of datasets were "Homo sapiens" as the organism choice; selected platform as "Illumina arrays including Infinium MethylationEPIC, Infinium Human Methylation27, and the Infinium HD 450 K methylation array”, respectively. We selected and analyzed relevant publications in the past 5 years (Table 1 and Table 2) to provide an overview of existing DNA methylation-based biomarker studies and to outline the progression and future aspects of this research domain. We seek to meticulously address the practical challenges faced by the researchers in the selection of methodologies and give an updated perspective on algorithms/packages used for processing array-based DNA methylation data. We also aim to present an end-to-end methodological framework that guides the selection of computational algorithms for diverse research outcomes and demonstrates advancement in the analysis of methylation array data for diagnostic and prognostic studies in disease pathology.

**Table 1:** Summary of studies reviewed focused on exploring methods and algorithms for the identification of DMPs/DMRs for different types of breast cancer and other diseased genome profiles.

S.no.	Algorithm	Samples size	Role	Survival analysis and Prognostic/diagnostic behavior analysis	Outcome	Reference
DNA methylation profile datasets analysis in various types of breast cancer						
1	Hierarchical linear models; Mann—Whitney U test	9 patients’ samples with LBBC and 5 non-tumor controls (raw IDAT files)	(i) Measures the accurate estimates of the methylation differences between different groups.	(i) Receiver operating characteristic (ROC) curve depicts the diagnostic behavior of genes.	(i) 28,799 differentially methylated CpGs	[106]
2	Univariate and multivariate Cox regression analysis	560 patients’ samples of breast cancer (raw IDAT files)	(i) This method screens the prognostic CpGs and survival analysis of patients.	(i) Methylation prognostic model deriving methylation-based gene prognostic signature	(i) Favourable prognosis: 66 and 49 CpG loci poor prognosis: 17 CpG loci.	[119]
3	Linear regression model	Eight vaccinated subjects' blood samples (raw IDAT files)	(i) Examine the association between DNA methylation and other variables such as antibody titers and IFN-γ production.	None	(i) 182 differentially methylated CpG probes	[48]
4	DMRcate Cox proportional	168 ILBC cases from TCGA, 130 (ILBC) tumors diagnosed (raw IDAT files)	(i) DMRcate was used to identify variably methylated regions (VMRs) in ILBC tumors	(i) Cox proportional hazards models were then used to assess the connection between the ten most important VMRs and their extent of methylation.	(i) 2771 variable methylation regions (VMRs)	[47]
5	Conditional or unconditional logistic regression, fixed-effect meta-analysis, Wald test, and Cochran’s Q test.	1663 breast cancer cases and 1885 controls from four different cohorts Cohort: MCCS, EPIC-Italy, the IARC cohort of the European Prospective Investigation into Cancer and Nutrition (EPIC-IARC), and the Prostate, Lung, Colorectal, and Ovarian Cancer Screening Trial (PLCO)	(i) The Minfi package allows normalization, quality control, and analysis of the data integrated from multiple studies. (ii) Logistic regression was used to determine the OR (odds ratio) and SE (associated standard error) per one standard deviation increase in methylation. (iii)Fixed-effects meta-analysis was used to calculate pooled OR and their associated SE. (iv) The calculation of P values is done by the Wald test.	(i) Adjusted logistic regression was used in each of the four investigations to evaluate the relationship between the risk of breast cancer and a one standard deviation increase in global DNA methylation.	(i) The validation of the blood DNA methylation in a large cohort is done, showing the probable risk of breast cancer. (ii) Individual CpG methylation analysis did not predict breast cancer risk.	[120]
6	Comparison of methylation levels of CpG sites. Houseman algorithm	23 TNBC tissues at DKFZ using two types of arrays: HumanMethylation450K (52 samples) and EPIC (71 samples). 221 Normal samples.	(i) The Houseman algorithm was applied for deconvolution to address cell-type heterogeneity in the methylation data. (ii) Differential methylation regions (DMRs) were identified by comparing methylation levels at selected CpG sites across TNBC tissues (with both sets of 123 samples) and 221 normal samples.	(i) An XGBoost model was trained to identify predictive CpG sites from selected DMRs.	(i) Identification of 23 DMRs containing 18 hypermethylated and 5 hypomethylated with 52 CpGs. (ii) Using XGboost, 23 DMRs are streamlined to 6 (SPAG6, LINC10606, and TBCD/ZNF750 genes) for increasing the diagnostic efficiency as they showed significant differences between the TNBC patients and healthy individuals.	[121]
7	ChAMP, linear mixed effects models	40 estrogen-receptor positive DCIS and 15 adjacent-normal tissues.	(i) ChAMP pipeline was used for preprocessing of DNA methylation data such as processing the raw IDAT files and other preprocessing steps. (ii) The application of linear mixed effects models to each CpG site separately can identify DMR in comparing DCIS and normal breast cancer samples.	(i) The relationship between time-to-event data and the extent of methylation at individual CpG sites is examined using Cox proportional hazards models.	(i) Significant 95,609 differentially methylated CpGs were identified. (ii) 641CpGs representing 397 genes were identified showing the relation between methylation extension and progression of the disease.	[122]
8	Linear model (limma) Kaplan-Meier plot	peripheral blood samples of diseased (breast cancer) and control.	(i) This method proves the differential methylation level and its relation with the occurrence of early-stage and late-stage breast cancer.	(i) Kaplan-Meier plotter analyses genes' prognostic significance in breast cancer relapse-free survival. (ii) Pearson correlation analysis tests DNA methylation changes and progression of breast cancer.	(i) 1902 DMPs (early-stage cancer) (ii) 30,312 DMPs (late-stage cancer).	[50]
9	Linear model T test Multivariable linear models	93 breast cancer cases and 43 control	(i) Minfi package was used for preprocessing methods including the processing of Raw data and application of the SWAN normalization method. (ii) Defined the beta value changes between the pre-and post-treatment blood samples for cases samples or in other cases, between the control samples at two subsequent time points, by the use of a linear model implemented in the limma method.	(i) Paired-t-test compares leukocyte composition changes in breast cancer patients and controls. (ii) Multivariable linear models were utilized for estimating the effects of various treatments and other factors.	(i) The biomarker genes in treated samples are VMP1/MIR21, SUMF2, CORO1B, and SDK1. (ii) cg16936953 (DMS), was also related to cognitive decline in patients with breast cancer.	[55]
DNA methylation profiles data analysis in different diseases.						
10	Linear model approach (limma) for DMPs bumphunter for DMR analysis	182 patients’ samples with knee osteoarthritis pain and 31 without pain.	(i) This method performs genomic segmentation and identifies DMRs using linear model approach for identifying the regions with a continuous trait.	(i) Pathway enrichment analysis for determining the canonical pathways and upstream regulators	(i) 13,951 hypermethylated CpG probes (high pain grade groups) (ii) 5759 hypomethylated CpG probes (low pain grade groups)	[123]
11	COHCAP package for screening DMGs. DEseq package for screening DEGs.	185 samples of patients suffering from Esophageal carcinoma	(i) COHCAP provides a comprehensive analysis of the methylation datasets, finding DMS/DMR. (ii) DEseq uses a negative binomial distribution to model the count data and calculates its variance-mean dependence as well.	(i) WGCNA Co-Expression Network analysis was used to find the correlation between the modules and the expressed genes.	(i) Obtained 2408 DEGs And 5134 DMPs. (ii) 10 hub genes after WGCNA analysis.	[105]
12	Limma and DMRcate package for screening of DMPs and DMRs	Samples of 9 healthy controls, 10 plaque-type skin psoriasis, and 7 PsA patients	(i) Minfi package was used for the preprocessing of the raw data. (ii) Usage of empirical Bayes moderated t-test method (*limma*) for estimating DMPs. (iii) Identification of DMRs using DMRcate package by combining CpGs with a cut-off β value, to extract DMRs.	(i) Significant annotated genes of DMPs showing a strong association of skin disease activity/PASI scores, are further used for calculating methylation scores.	(i) 397 DMPs ((healthy and (combined skin psoriasis and PsA) patients’ samples. (ii) 1861 DMPs (b/w psoriasis vs. PsA patient). (iii) 2372 DMPs concerned with initiation of treatment. (iv) Methylation score differentiates disease activity from remission achievement.	[114]
13	*Limma* for DEGs and DMPs	Cervical cancer samples – 39 and normal cervix samples - 44	(i) The annotated differential genes are derived using the empirical Bayes method (*limma*). (ii) Aberrantly methylated DEGs are found by overlapped oncogenes, tumor suppressors, and DE genes.	(i) Functional enrichment and validation by Gene Expression Profiling Interactive Analysis (*GEPIA*).	(i) 1313 DEGs and 1405 DMGs from limma. (ii) Aberrantly methylated DEGs: 32 hypomethylated $ up-regulated genes, including 2 oncogenes; 44 hypermethylated $ down-regulated genes, including 8 TSG.	[98]
14	*Limma* is used for DEGs and ChAMP for DMGs.	Methylation dataset: 78 samples with 32 healthy and 46 diseased. Gene expression dataset: 19 samples with 10 healthy and 9 diseased.	(i) The annotated differential genes are derived using the empirical Bayes method (*limma*). (ii) WGCNA analysis-derived gene modules enhance biomarker diagnostic accuracy. (iii) ssGSEA investigates the immune infiltration landscape for disease progression in samples.	(i) Gene co-expression network constructed. WGCNA package using DEGs. (ii) Single-Sample Gene Set Enrichment Analysis (ssGSEA)	(i) 268 co-expressed genes from WGCNA analysis. (ii) 77 common genes are derived from comparing the result of WGCNA analysis and DMPs. (iii) Benchmarked top ten biomarker genes (iv) ssGSEA reveals ITGA2 and BCL2 correlation with Th2 and Th17 cells.	[100]
15	Limma for DEGs and DMGs	Gene expression dataset: 306 samples of patients from TCGA portal, Methylation dataset: 215 samples from GEO portal	(i) The annotated differential genes are derived using the empirical Bayes method (*limma*). (ii) Estimate algorithm finds immune and stromal cell concentration using gene expression data, targeting TCGA portal.	(i) Application of estimate algorithm. (ii) Validation of key genes by survival analysis and TIMER database.	(i) 1401 genes in stromal score and 1781 genes in the immune score group. (ii) stroma-immune score groups derived 776 upregulated and 26 downregulated genes. (iii) 10 prognostic biomarkers are benchmarked as TME signatures.	[99]

a. LUAD- Lung squamous cell Adenocarcinoma.; b. GDM- Gestational Diabetes Mellitus; c. RF-Random Forest; d. OSCC-oral squamous cell carcinoma; e. OPMDs- oral potentially malignant disorders; f. AIN3- Anal intraepithelial neoplasia-3; g. CIN3- cervical intraepithelial neoplasia-3; h. ccRCC- clear cell renal cell carcinoma; i. DS-Down syndrome; j. CHD- congenital heart defect. k. DMPs: Differentially methylated positions, l. DEGs: Differentially expressed genes, m. DMGs: Differentially methylated genes, n. *GEPIA:* Gene Expression Profiling Interactive Analysis, o. ssGSEA: Single-Sample Gene Set Enrichment Analysis, p. TNBC: Triple-negative Breast Cancer. q. DCIS: Ductal carcinoma in situ, r. TNBC: Triple-negative Breast cancer, s. DCIS: Ductal carcinoma in situ. t. ILBC: Invasive lobular Breast Cancer.

**Table 2:** Comprehensive summary of the Machine learning and Deep Learning algorithms utilized in the reviewed studies for classification models, emphasizing their significance in the research.

S.no.	Sample Size and platform	Role of algorithms	Feature selection algorithm	Candidate marker	Prognostic /classification models	Pros. and cons.	Ref.
1.	1188 samples from Breast Invasive Carcinoma project from GDC Data Portal. Platform: Illumina 27 K and 450 K methylation data (TCGA database)	(i) Disease prediction using high-dimensional methylation data, handling missing data, and preventing overfitting. (ii) Trained on the reduced feature set which improves the accuracy of the model.	(i) Dimensionality reduction by ANOVA and Random Forest. (ii) Handling data imbalance: SMOTE	685 CpG markers for 27 K array and 1572 CpG markers for 450 K array	(i) Deep neural network (DNN) classifier with three hidden layers and dropout regularization to predict breast cancer using the selected methylation markers. (ii) Accuracy: 98.75%	Pros: Increased predictive accuracy while maintaining scalability. Cons: Increased risk of overfitting with too many features.	[132]
2.	653 samples from UCSC Cancer Browse and TCGA-LUAD portal. Platform: Illumina Infinium data from UCSC cancer browser.	(i) The model aids in biomarker identification, prognosis prediction, diagnosis, and management of LUAD subtypes. (ii) Identify patients who are at high risk of poor prognosis.	(i) Univariate Cox regression. (ii) Multivariate Cox model	205 independent prognosis-related CpG sites	(i) Consensus clustering method concluding seven molecular subgroups of DNA methylation. (ii) The AUC of test samples is 0.788.	Pros: Robust nature as compared to single-run clustering algorithms Cons: This can struggle to identify the true optimal cluster number for known data.	[139]
3.	268 samples of gene expression Data and 203 tumors samples of DNA methylation data with 94 normal samples. Platform: Illumina HumanMethylation27 BeadChip. (from GEO database)	(i) This model can train itself from the high dimensional methylation data. (ii) The classifier tests the accuracy of the classification.	(i) Mutual information (MI), FC, T-test, and FDR test. (ii) Overlapping genes are used for dimension reduction.	New expression matrix using Gene and methylation expression matrix.	(i) DNN classifier model giving as an output of overlapped eight genes that appear in each fold of cross-validation (ii) accuracy: 98.7%.	Pros: The comparative analysis of DNN with other ML classifiers, gives superior results. Cons: Increased risk of overfitting with too many features.	[133]
4.	342 samples of 27 K array and 890 samples of 450 K array. Platform: Downloaded from UCSC Cancer Browser	(i) The Cox regression model is a prognostic model for breast cancer patients using CpG sites and clinical factors; Reduces the dimensionality of the data.	(i) Univariate and Multivariate Cox regression algorithm	166 prognosis-related CpG sites.	(i) Prognostic model using Cox regression model. (ii)Consensus clustering for classification. (iii) AUC: 0.757	Pros: Well known for prognosis, tumor classification, and survival outcomes. Cons: Assuming that continuous variables linearly influence the log hazard.	[141]
5.	393 stage I–II LUAD samples. Platform: Illumina HumanMethylation450 BeadChip (TCGA database).	(i) The feature selection method evaluates core methylation sites for LUAD patients' overall survival. (ii) The prognostic hallmark of the disease is estimated by Multivariate Cox regression analysis.	(i) Univariate Cox regression and LASSO regression model. (ii) Multivariate Cox regression analysis	11 methylation signatures for OS stage I-II LUAD patients.	(i) Patients with stage I–II LUAD were categorized into “high-risk” or “low-risk” cohorts based on a high and low-risk score. (ii)Nomogram construction signifies the risk score-survival relationship. (iii) AUC at 1, 3, and 5 years was 0.747, 0.818, 0.870.	Pros: This method determines the association of overall survival with methylated signatures. Cons: Assuming that continuous variables linearly influence the log hazard.	[143]
6.	183 periodontitis patient samples and 64 healthy control samples. Platform: Illumina HumanMethylation450 BeadChip from GEO database.	(i) WGCNA constructs a co-expression network of CpGs and identifies modules of highly correlated CpGs. (ii) SVM handles non-linear relationships between input features and output variables.	(i)Limma for identification of DMPs. (ii) Weighted gene co-expression network analysis (WGCNA).	(i) Screening of 8029 differentially promoter regions and annotation of 4940 genes. (ii) 23 Co-DMPs	(i) The outcome of the SVM model was 5 CpG biomarkers identified for periodontitis. (ii) Classifier accuracy: 95.5%.	Pros: High model accuracy for diagnostic biomarkers. Cons: Limited to the binary class problem.	[93]
7.	195 samples of pancreatic cancer (PC) Platform: Illumina Human Methylation 450 downloaded from UCXC Xena platform.	(i) LASSO algorithm selects CpG sites from larger variables for cancer prognosis. (ii) Nomogram model graphically represents prognostic factors and risk scores, aiding clinicians in estimating patient survival probability.	(i) LASSO risk score model predicts CpG prognosis using 5 CpG markers and 111 CpG expression sites data of the training set samples.	Data from risk score model and clinical factors.	(i) Nomogram model formation which enhances the prognosis of PC patients. (ii) AUC at 1, 3, and 5 years was 0.70, 0.77, and 0.83.	Pros: Effective tool for prognosis of patients. Cons: not applied on the large sample size.	[86]]
8.	7339 patients of 18 pan-cancer origins. Platform: Illumina Human methylation 450k BeadChip downloaded from TCGA and GEO databases.	(i) Feature selection algorithm was used for selecting significant CpG sites. (ii) DNN model classifies cancer origin, and metastatic origin, and predicts cancer cell types.	(i) ANOVA and Tukey’s honest significance difference test was performed on the training data.	10360 CpG sites	(i) 3–5% metastatic cancers from samples with unknown primary origin were estimated with 80% having poor prognosis with OS of 6–10 months. Also, cancer cell type prediction was done using this DNN classifier. (ii) Accuracy: 95.03%	Pros: Highly robust nature Cons: This model is applicable for small sample sizes.	[131]
9.	68 umbilical cord blood samples and 64 controls Platform: Illumina Human methylation 450k BeadChip downloaded from GEO databases.	(i) SVM model predicts GDM occurrence using significant methylation CpG sites.	(i) Minfi package was used for assessing the quality of the CpG sites. (ii) CpGassoc R package was used for finding the significant CpG methylation sites.	89 high-quality CpG sites.	(i) The model utilized 6 CpG sites, allowing the diagnosis of the disease. (ii) AUC: training and testing set in the model were 0.8138 and 0.7576.	Pros: The SVM model is widely used for the diagnosis and prognosis of disease. Cons: Limited to binary class problem and needs further optimization.	[134]
10.	The TCGA platform was used for downloading level 3 DNA methylation data and clinical data.	(i) mRMR feature selection (wrapper method) uses relevance and mutual dissimilarity to select features. (iii)Wrapper method: optimizes features using genetic algorithm, focusing on optimal subsets for maximum prediction performance. (iv) RF: feature selection evaluation.	(i) Wrapper method: minimum redundancy maximum relevance (mRMR); method based on genetic algorithm. (ii) Filter method: DNA methylation analysis.	(i) 12 probes with 14 genes (LN+ and LN-) (ii) 20 probes with 39 genes (normal-tumor) (iii) 6 genes identified as lymph node metastasis-related genes.	(i) Model classifies samples into normal, LN metastasis negative, and LN+ , reliably predicting stomach cancer lymph node metastasis. (ii) accuracy: greater than 0.99	Pros: Reduces the overfitting risk of the prediction task. Cons: Feature selection is dependent on their relevance to the target variable which may result in the selection of redundant or irrelevant features.	[152]
11.	Experiment stage: 458 NSCLC samples. Validation: 150 normal samples. Platform: microarray data from GEO and ArrayExpress.	(i) Feature selection reduces dimensionality, and selects relevant signatures for sample classification. (ii) These models were used to evaluate the diagnostic efficiency of the biomarkers.	Support Vector Machine and Random Forest algorithm.	5 gene methylation signatures	(i) Logistic regression model, support vector machine (SVM), Random Forest, and Bayes tree model used for classifying NSCLC tumor and normal tissue samples. (ii) Bayes trees proved to be a powerful classifier with an AUC of 0.906.	Pros: This diagnostic panel could significantly enhance prediction performance for clinical utility. Cons: The computationally intensive algorithm used for model construction.	[163]
12.	143 anal carcinomas: 9 normal, 13 AIN3, and 121 tumor samples. 28 cervical carcinomas: 10 normal, 9 CIN3, and 9 tumor samples. Platform: Illumina HumanMethylation450 BeadChip downloaded from GEO	(i) Models contribute to the extraction of essential methylations and construct efficient classifiers and classification rules.	(i)Boruta feature filtering; Feature ranking algorithms. (ii)Monte Carlo feature selection (MCFS); Light gradient boosting machine (LightGBM) and LASSO.	571 methylation features for anal carcinoma and 26 features for the cervical carcinoma dataset	(i) Incremental feature selection (IFS) is used as the classification method using a Decision tree and Random Forest algorithm. (ii) The RF classifier shows better performance.	Pros: the model depicted methylation patterns on different stages for two carcinomas. Cons: The Boruta algorithm is effective for a small sample size with an adequate number of features.	[158]
13.	1473 sarcoma samples Platform: Illumina HumanMethylation450 BeadChip downloaded from GEO	(i) The ordered selected feature list was fed to classification models for attributing high performing models, essential methylation sites, and quantitative classification rules.	(i)Boruta feature filtering; Feature ranking algorithms. (iii)Monte Carlo feature selection (MCFS); Light gradient boosting machine (LightGBM) and LASSO.	(i) Final selection of 8954 features. (ii) common genes from LASSO, LightGBM, and MCFS feature lists.	(i)Incremental feature selection (IFS) is used as the classification method using a Decision tree and Random Forest algorithm. (ii) The RF classifier shows better performance (F1: 0.971-0.940-0.978) with LASSO-LightGBM-MCFS (110-30-310).	Pros: Identified essential methylation sites and their respective classification rules. Cons: The Boruta algorithm is effective for a small sample size with an adequate number of features.	[159]
14.	332 samples from the GEO database Platform: Illumina HumanMethylation450 BeadChip downloaded from GEO and TCGA	(i) The LASSO method reduces irrelevant feature coefficients to zero. (ii) RF measures feature importance by calculating the decrease in mean accuracy.	LASSO analysis and random forest analysis.	5 markers were found by the intersection of feature selection algorithms.	(i) Random forest classifier predicts high-risk and low-risk populations. (ii) AUC: greater than 0.8.	Pros: The model shows good diagnostic and predictive efficiency. Cons: Validation of the model is required with the use of external large sample-size datasets.	[155]
15.	150 samples from high blood pressure patients Platform: Illumina HumanMethylation450 BeadChip array data	(i) DL is a type of neural network that can learn complex patterns of methylation expression in data. (ii) RF enhances model accuracy and robustness through ensemble learning and SVM is a popular algorithm for classification tasks that finds the optimal hyperplane for separating data.	PCA for selecting principal components.	Not specified	(i) Random Forest, Deep learning, and Support Vector Machine (SVM) model for classification. DL shows high predictive accuracy (F1: 0.73) (ii)Developed for predicting high blood pressure using methylation samples of patients.	Pros: Best ML algorithms are used for disease prediction. Cons: A small sample size can limit the learning ability of the model.	[156]
16.	Methylation: 315 COAD samples and 38 tumor-adjacent samples, Transcriptomics:41 cases from tumor-adjacent tissues, 480 cases from COAD tissues. Platform: Illumina Human Methylation 450 Beadchip (450 K array) from TCGA portal.	(i) Methylmix r package integrates gene expression and DNA methylation data. (ii) Cox regression analysis identified MDGs significantly associated with OS in the training set. (iii) The LASSO algorithm handles largely correlated covariates inpatient cohort model construction.	(i) Limma, edger, and Methylmix r package for finding MDGS. (ii) LASSO and univariate Cox regression analysis for screening the MDGs.	Ten MDGs as candidate Biomarker for colon cancer	(i)Multivariate Cox regression was used for constructing a best‐fitting prognostic model, giving an output of a panel of six genes.	Pros: Exploring the potential use of DNA methylation as a prognostic biomarker. Cons: No external validation was performed whereas the internal validation can result in random noise of the methylome.	[142]
17.	95 samples of thymoma or thymic carcinoma patients Platform: Illumina 450 K array downloaded from UCSC browser.	(i) Univariate Cox regression identifies methylation sites related to recurrence-free survival (RFS) in TETs. (ii) Multivariable Cox regression gives prognostic factors for RFS.	(i) Univariate Cox regression on the functional methylation sites.	52 CpG sites	(i) Cox proportional hazards regression models predict risk subgroups using median RRS. (ii) The log-rank test and the Kaplan-Meier method were used for comparing RFS between the subgroups.	Pros: the model used for identifying methylation signature for predicting disease reoccurrence [HR = 2.718, 95%]. Cons: Assuming that continuous variables linearly influence the log hazard.	[140]
18.	86 NDBS samples from two groups: (i) 45 DS-CHD (27 females, 18 males). (ii) 41 DS non-CHD (27 females, 14 males) Platform: Illumina Infinium Methylation EPIC (EPIC) array data.	(i) The ipDMR method identifies DMRs by comparing DNA methylation levels and adjusting CpG correlations. (ii)Classification model used for distinguishing CHD and non-CHD.	None	Classification model using ML algorithm such as Random Forest and SVM	(i) 58, 341, and 3938 DMRs were derived from the Combined Sex group, Females Only, and Males Only groups, respectively. (ii) ML algorithms select 19 Males Only loci that differentiate CHD from non-CHD.	Pros: High model accuracy for diagnostic biomarkers. Cons: SVM is limited to binary class problems and needs further optimization	[65]

a. WGCNA: weighted gene co-expression network analysis, b. PC: pancreatic cancer, c. MCFS: Monte Carlo feature selection; d. LightGBM: Light gradient boosting machine, e. LASSO: Least absolute shrinkage and selection operator, f. SVM: Support Vector Machine, g. PCA: Principal component analysis, h. DL: Deep learning, i. RF: Random Forest, j. MDGs: Methylation-driven genes, k. NDBS: newborn dried blood spots, l. DNN- Deep Neural Network, m. LUAD- Lung squamous cell Adenocarcinoma, n. GDM- Gestational Diabetes Mellitus, o. RF-Random Forest, p. OSCC-oral squamous cell carcinoma, q. OPMDs- oral potentially malignant disorders, r. AIN3- Anal intraepithelial neoplasia-3, s. CIN3- cervical intraepithelial neoplasia-3, t. ccRCC- clear cell renal cell carcinoma, u. DS-Down syndrome, v. CHD- congenital heart defect.

**Fig. 2:**
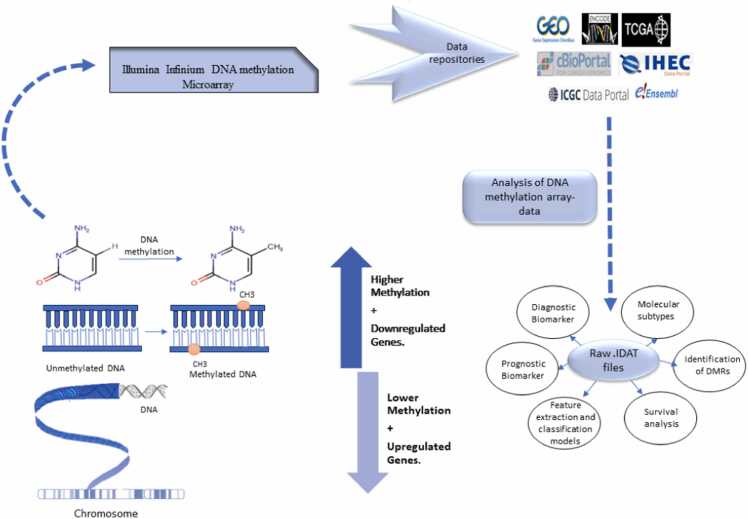
General concept showing the flow of DNA methylation profiling data from experimental methods to data repositories and providence of DMR analysis algorithms. [212].

Taking account of the multiple hypotheses and the methodology designed by the investigators, the review has been divided into six sections with their respective subsections. The first section focuses on how the investigator retrieves the methylation datasets supported by a variety of filters to meet the requirement of the research objective. The second portion delves into preprocessing requirements and algorithm selection criteria to improve the analysis accuracy. The third section addresses exploratory analysis showcasing the clustering algorithms undertaken by different studies for better characterizing and visualizing the patients’ samples. The proceeding sections detail the downstream analysis with separate subsections discussing the algorithms/methods and packages used specifically for identifying DMRs which can serve the purpose of diagnosis and prognosis of the disease. Also, we briefly address the algorithms used to identify genome segments with similar methylation patterns under a single condition. In the penultimate section, we explore the integration of feature selection algorithms and the development of ML/DL models, which aid in predicting the estimated risk of disease occurrence and progression, and also determine overall survival efficacy for derived significant methylation biomarkers. Lastly, the process used for evaluating the biological relevance and functional significance of methylated regions is discussed in detail. More clarity on the step-wise analysis of methods followed in the relevant articles along with their respective multiple-hypothesis taken into consideration for this review article as illustrated in Fig. 2.

## 2 Microarray dataset collection and repositories

To begin, the primary DNA methylation workflows make use of global repositories such as the GEO databases that are supported by the National Institute of Health (NIH). Additionally, the TCGA-GDC portal (http://cancergenome.nih.gov/) is a collaboration that is supported by the National Cancer Institute (NCI) and the National Human Genome Research Institute (NHGRI) for the collection of DNA methylation raw data[47], [48], [49], [50], [51], [52]. The terms "organism of interest," "study type or experiment type," "control and diseased datasets," and "raw data type occurring in. IDAT format/TXT format" are the filters that can be utilized during the collection of datasets from databases. The users have access to several global repositories that assist in the storage of comprehensive DNA methylation array data obtained from high-throughput sequencing technology and genome profiling data. Various noticeable repositories are depicted in Supplementary Table I, which gives an overview of the different diseased datasets, datatypes of the available datasets, their description, the input provided by the user, and the output format of the datasets. This table also includes a separate section of the epigenomic repositories solely dedicated to cancer datasets. The Supplementary Table II contains a detailed description of the datasets—covering their source, sample size, representativeness, and selection methods—referenced in the majority of research papers cited in this review article. In all reported studies, the algorithms used for the processing of the methylation array dataset are chosen wisely with the strong recommendation of the Methylation profiling data via the Illumina InfiniumHumanMethylation BeadChip Assay platform.

## 3 DNA methylation microarray data analysis

The analysis procedure is somewhat analogous to the analysis patterns that are being followed in various other analyses of sequencing data. The complete procedure could be broken down into the following four broad sections, each of which can be further subdivided.

### 3.1 Pre-processing analysis of raw dataset

The major algorithms, which are among the most popularly used ones in published research for pre-processing of the raw data include SWAN-subset-quantile within array normalisation [53], [54], [55], FunNorm- Functional normalization [54], [56], pQuantile-stratified quantile normalization [57], noob-normal-exponential using out-of-band probes [54], [58], RCP-Regression on Correlated Probes [59], BMIQ-Beta-Mixture Quantile Method [50], [60], [61], [62], [63] and the combination of noob and BMIQ, that was shown to give better performance than others[58]. Further detailing of the normalization algorithms and packages can be found in Supplementary Table III. Such pre-processing methods also target several common computational manipulations on raw data. These include background signal subtraction, color bias adjustment, and probe type adjustments to reduce the effects of experimental variation early in the pipeline. Also, it is necessary to check for missing values in the genomic regions of the patient samples which can lead to technical biasness. This can be overcome by employing imputation functions based on k-nearest neighbors using an Euclidean metric method for simple data. This is followed by inter and intra-sample normalization as well as batch effect correction, which are typically taken into account later in the pipeline. Moreover, in epigenome-wide association studies (EWAS), cellular composition can confound the association between primary phenotype and methylation levels. To address this, the researcher can use functions such as estimateCellCounts offered by *Minfi* and FlowSorted.Blood.EPIC Bioconductor R package, a reference-based deconvolution method [64], [65], and the methylDeConv R package (capable of analyzing both Illumina 450k and EPIC arrays), to estimate and account for this confounding effect [55], [66], [67]. Hence, considering the required steps for selecting an appropriate pre-processing pipeline is a must to improve statistical efficacy in single dataset analysis and ensure result reliability. Supplementary Table IV details the preprocessing methods utilized by the packages/functions frequently mentioned in the majority of research articles.

Despite the diversity of the studies discussed in this review, some common preprocessing steps can be identified in their methodologies. These steps are outlined below:

a) Filtering of probes:

• Remove probes with detection p-value greater than 0.01 or 0.05, low bead count, SNPs, or cross-hybridization potential.

• Remove CpG sites with no or small differences in beta values among tissues and remove probes on the sex chromosomes.
• Reject samples with too many poorly performing or missing probes.
• Some methodologies only selected CpG sites present in the promoter regions.

b) Quality control:

• Apply background subtraction using methylumi or other packages.
• Filter out PCA outliers using various methods.

c) Imputation of missing values:

• Remove or impute missing values using Impute or ENmix packages.
• Use different methods such as KNN, mean, or iterative imputation.
• Use Bayesian Ridge regression for probabilistic estimation of missing values.

d) Batch correction and FDR correction:

• Use the ComBat algorithm to correct for batch effects.
• Use the Benjamini-Hochberg method to control for multiple testing.

e) Normalization of samples:

• Use different methods such as betaqn, BMIQ, SWAN, color bias correction, SVD, normalizeBetweenArrays, functional, or funNorm.
• Regressed out variability explained by control probes.

### 3.2 Exploratory analysis

Numerous studies have shown that utilizing visual inspection and graphical representation of normalized and quality-controlled DNA methylation data aids in detecting global changes in methylation patterns. This preliminary analysis precedes more intricate investigations related to differential DNA methylation. These methods also facilitate the detection of methylation gains or losses, and exploration of methylation levels in specific genomic regions across diverse samples, enabling comparative analysis of methylation patterns.

#### 3.2.1 Clustering samples

The process of clustering is the procedure that is utilized to divide the data items into many groups or subgroups within a set of given samples. For instance, the application of the clustering-based approach can differentiate the DNA methylation data into clusters of normal and abnormal DNA profiles data; hypermethylated and hypomethylated probes in the DNA methylation array data; and clusters with and without CIMP (CpG island methylator phenotype).

##### 3.2.1.1 Clustering methods

PCA (principal component analysis) is a classic clustering method used by Tirosh et al., 2022 for visualizing the association/comparison of DNA methylome signatures between the groups of Neuroendocrine tumors and normal samples, for better characterization [68]. Despite its widespread use as a dimension reduction procedure, PCA’s main drawback is the difficulty in interpreting the independent variables that form the principal components, along with the necessity for a large sample size to ensure reliable outcomes. Another significant algorithm is Hierarchical clustering, which creates a binary tree by progressively combining samples or probes that are alike, using a specific similarity measure [69]. Significantly, this clustering analysis identified a distinct DNA methylation pattern that effectively distinguished CTC-MCC-41 cells from HT29 cancer cells, in colorectal cancer patients [70]. However, the unsupervised nature of hierarchal clustering does not allow the algorithm to use data beyond methylation for making clusters and eventually can fail to predict a phenotype required by the user [71]. Therefore, Kok-Sin et al., 2015 used an advanced method for binary sample distribution analysis, which includes both supervised hierarchal clustering and PCA for subgrouping the differentially methylated loci and methylation ratio matrix of genes, into hypermethylated and hypomethylated loci for finding a significant locus/gene, respectively [72]. For complex data distribution, the tSNE (t-distributed stochastic neighbor embedding) and NMF (non-negative matrix factorization) algorithms are used for the dimensionality reduction step, along with their contribution towards disease stratification [73]. t-SNE and NMF demonstrate robust performance in managing outliers and in the unsupervised modeling of cancer diagnosis, respectively, outperforming PCA in terms of efficacy [74], [75]. t-SNE's need for precise hyperparameter adjustment and high computational demands pose challenges, contrasting with NMF's limitations related to non-negative beta-valued methylation data assumptions and parameter sensitivity [75], [76]. In recent times, Amor et al., 2022 developed the deep-embedded refined clustering (DERC) method showing a better approach than PCA, tSNE, and NMF, by using autoencoders for performing unsupervised classification of breast cancer samples among normal samples. The accuracy of the method achieved is 0.99 [77].

For interpreting the tumor heterogeneity of cancer tissues or classifying the tumor samples, classic unsupervised/supervised methods like K mean clustering stratify samples based on CIMP status, affecting tumor differentiation of colorectal cancer [78]. K-means clustering is user-friendly and adaptable for large samples or diverse methylation patterns, but it may need multiple attempts to overcome its randomness and high computational demand, with the requirement of dimensionality reduction techniques for better efficiency [79], [80]. Similarly, Hosseini, M et al., 2023 used the hierarchal clustering algorithm for visualization, showing the separation between the significant hypermethylated probes related to promoter region into tumor and non-tumor classes. The researchers further utilized the Pearson correlation and recursive feature elimination with the 10-fold cross-validation (RFECV) methods for filtrating features to identify diagnostic biomarkers in stomach adenocarcinoma [81]. In this aspect, hierarchical clustering is often seen as a more user-friendly algorithm than K-means, because it provides easily interpretable dendrograms, deeper insight into sample relationships, and careful interpretation due to its sensitivity towards noise and outliers. Apart from these non-parametric approaches, the recursively partitioned mixture model (RPMM) has been applied to cluster DNA methylation and hydroxymethylation data for tumor classification using beta values [82]. Interestingly, Azizgolshani et al., 2021 employed this algorithm for perceiving the association between the 5hmC signals of CNS tumor samples with overall survival, suggesting that low 5hmC patterns have an increased risk of recurrence and poor overall survival (OS) rate [83]. One of the advantages of the RPMM approach is the robust computational efficiency over traditional finite mixture models and it can integrate data related to CpG sites on the genomic locations to create biological correlational structures that become the basis of further clustering study. Also, its notable limitation can be its inability to include established biological correlations of the measured features [71], [84].

Several clustering algorithms are utilized to subgroup differentially methylated sites (DMS), serving as prognostic models, capable of predicting risk scores or classifying patients into distinct molecular subtypes. These algorithms leverage information from the analysis of overall survival on samples to accomplish this task. For instance, Consensus clustering is employed by *Wang et al., 2021* for subgrouping prognostic methylated CpG site into four methylation clusters, reflecting the variation in molecular genetic features (determined by hypermethylated/hypomethylated loci) concerning the prognostic behaviour of the cluster group [85]. Similarly, Yin, X. et al., 2021 evaluate such molecular subgroups of pancreatic cancer samples, for poorer prognosis and its associated clinicopathological features [86]. In a related study, BRCA samples were subgrouped through consensus clustering, which utilized methylation data of methylated-driven genes (MDG). This involved subsampling the data matrix and categorizing each subset into 'k' clusters via K-means. The subgroups' overall survival was analyzed using Kaplan-Meier plots, and the significance of differences between clusters was evaluated using the log-rank test [87]. Basically, Consensus clustering is a more dependable method by repeatedly applying a selected clustering algorithm to different subsets of the data, leading to its robust nature as compared to single-run clustering algorithms. It is extensively utilized to identify clusters linked to various clinical outcomes [88].

### 3.3 Downstream analysis

This refers to the sequence of analytical procedures conducted following the collection and preprocessing of the raw methylation data. It involves multi-sample dataset analysis such as the identification of regions with significant methylation changes, annotation, enrichment, and classification.

#### 3.3.1 Identification of differentially methylated regions (DMR)

The DMRs are composed of closely related DMS/DMCs (Differentially methylated sites) referring to individual CpG sites or small genomic regions that exhibit differential DNA methylation levels. DMRs differ in the methylation level of genomic features, including gene promoters, enhancers, CpG islands, and intergenic regions, particularly across distinct biological conditions (e.g., normal vs. disease) [89]. Most studied algorithms collectively focus on the DMR/DMC analysis in the methylation profiling data of the disease for screening the hypermethylated and hypomethylated genes, based on the significant user-selected margin of FDR, log fold change (FC), and p-value. The collective description of the packages along with their major role in computational analysis workflows such as preprocessing, DMRs/DMPs identification, annotation, and visualization are detailed in tabulated format (Supplementary Table V). Theoretically, aberrant DNA methylation affects gene expression in diseased pathways, causing suppression. Addressing this aim, multiple workflows have been developed to explore the correlation between methylation and gene expression data analysis. These methodologies are thoroughly examined in the following section.

First, a common strategy involves identifying hypermethylated-downregulated and hypomethylated-upregulated genes through separate analyses of gene expression and DNA methylation datasets. Subsequently, hub genes are retrieved by predicting protein-protein interactions (PPI network) among these identified genes. For instance, the identification of the aberrantly methylated differentially expressed genes was done by comparing the raw data grouped as tumor and normal samples of Oesophageal squamous cell carcinoma and bladder cancer, using GEO2R [90], [91]. Also, Cheng et al., 2022 used the DMRcate algorithm offered by the ChAMP pipeline to process 19 carotid atherosclerotic and 15 control aortic tissue samples for screening differentially methylated genes [92]. Further exploring the epigenetic regulation in the promoter region, Wang et al.,2021 derived 8029 differential CpG sites with 4940 genes annotated to the promoter region by using the empirical Bayes moderate T-test (*limma),* after comparing the 64 normal and the 183-periodontitis patient’s sample. This was followed by Weighted co-expression analysis for identifying immune-related co-expression patterns involving differential CpG sites [93]. Using the same method, Feng et al., 2021 analyzed methylation in blood samples of 39 ARDS and 30 control patients, suggesting hypomethylation may increase hub gene expression [94]. Also, this algorithm was utilized for preprocessing and differential expression of 371 HCC and 50 normal controls for designing a diagnostic signature model, giving five top methylated markers [95]. Likewise, Raman et al., 2018 extracted methylated genes and DEGs in pancreatic cancer patients, comparing survival- (<1 year) and survival+ (>2 years) groups using the *limma* package. The validation of the survival signature genes with high DNA methylation extend, is done by ROC and Kaplan-Meier survival analysis [96]. The same protocol was followed by Liang et al., 2019 and Zhang et al., 2019 for finding DEGs and DMGs with an additive assessment of Spearman’s correlation analysis for predicting the MeDEGs (methylated differentially expressed genes) in colon cancer and glioblastoma multiforme samples, respectively [91], [97]. Ma et al., 2020 used the Empirical Bayes t-test model for identifying aberrantly methylated DEGs/DMGs in comparing 39 diseased samples with 44 controls followed by identification of intersecting nodes between the list of 1313 DEGs; 1405 DMGs; oncogenes, and tumour suppressor genes [98]. Correspondingly, Xia et al., 2023 generated tumor signatures of cervical cancer by doing the expression analysis using limma on 306 cervical squamous cell carcinoma and endocervical adenocarcinoma (CESC) from TCGA and 215 CESC patients from the GEO portal. This was coupled with the application of an estimate algorithm, specialized in estimating the concentration of immune and stromal cells in the samples by using the gene expression data only (targeting the TCGA portal) [99]. Similarly, the gene co-expression network was constructed by importing DEGs to find the 268 co-expressed module genes in Alzheimer’s disease. These were subsequently compared with differentially methylated positions (DMPs) identified through ChAMP analysis, leading to the identification of 77 common genes [100]. Therefore, limma is frequently utilized for identifying genes, differentially methylated positions (DMPs), and differentially methylated regions (DMRs) due to its implementation of empirical Bayesian methods, ensuring robust outcomes even with limited sample sizes [101]. What's more, due to the comprehensive and specific approach of the ChAMP pipeline towards DNA methylation analysis, it is capable of conducting end-to-end analysis of DNA methylation microarray data (both EPIC and 450k data) and ensuring consistency with user-friendly interfaces [24]. To date, much of the literature has focused on the concurrent identification of DEGs and DMPs/DMRs to explore correlations between them. However, some algorithms specifically address the analysis of methylation data emphasizing solely on DMR investigation from array-based methylation data. Algorithms such as Comb-p, DMRcate, Bumphunter, and probe lasso suggest its identification in the promoter region with the decreasing order of their performance evaluation in terms of power, sensitivity, DMR size, DMR overlap, and the simulated time consumption [102], [103]. Therefore, a recent study done by Zhang W et al., 2023 implemented the use of *Comb-p* for identifying DMRs related to the CSF biomarker in Alzheimer’s patient's blood samples, to collectively identify the regions showcasing the adjacent low p-values [104], [85].

Second, in the quest for understanding the intricate regulatory mechanisms underlying gene activity, several studies admit correlation analysis between methylation patterns with gene expression levels revealing how epigenetic marks [105] A sophisticated study done by Yanzhao Xu et al., 2021 put forward the use of Fisher’s exact test offered by the COHCAP R package for estimating the differentially methylated sites on raw data of 170 samples of Oesophageal carcinoma (cases and control) and then undergoing WGCNA Co-Expression Network analysis for the identification of the hub genes [105]. As discussed earlier, *limma* is a popular choice among researchers due to its versatility in analyzing various omics data types. However, COHCAP is also recognized as an efficient algorithm specialized for methylation data analysis and its influence on gene expression. Additionally, the introduction of WGCNA Co-Expression Network analysis enhances the depth of biological insight into diseases by identifying gene modules (clusters) with similar expression patterns. These modules frequently correspond to biologically relevant pathways or processes, containing related hub genes within the module. Another study done by Rodriguez et al., 2022, used Hierarchical linear models (HLM) coupled with the Mann-Whitney U test to measure the accurate estimates of the methylation differences in the expression data between different tumour and non-tumour groups of breast cancer patients, to account for promoter hypermethylation of *WNT1* [106]. Over time, Hierarchical Linear Model (HLM) is preferred for its robust handling of large amounts of missing data and its ability to estimate individual changes over time with fewer assumptions [107]. Another statistical test commonly employed for correlation analysis is the estimation of Pearson’s correlation coefficient. It is utilized to explore the association between DNA methylation data and expression data related to exons or isoforms [108]. Notably, it’s important to be attentive towards potential non-linear relationships between variables, the existence of outliers, and limitations due to restricted data ranges [109]. Furthermore, mapping methylation signals to specific genomic coordinates based on microarray probes is a common approach in methylation data analysis, but it can encounter challenges, resulting in reduced sensitivity and increased false positives. To address this, regression algorithms, such as linear regression models, are employed to find the association between gene expression (dependent variable) and DNA methylation levels (independent variables). As a result, a novel combinatorial framework was introduced, integrating DNA methylation data with TCGA gene expression data. This framework combines linear regression, differential expression analysis, and deep learning techniques to enable accurate biological interpretation [110]. Hence, Linear regression and deep learning algorithms are good choices for identifying complex relationships between DNA methylation signatures and gene expression data.

Third, researchers have harnessed statistical methods to unravel the intricate relationship between DNA methylation patterns and various biological processes or pathways. These investigations focus on statistical approaches and some machine learning algorithms for exploring the epigenetic mechanisms. For instance, a study done by Yeung KS, et al., 2017 on Systemic Lupus Erythematosus Patients stated the use of a Wilcoxon rank-sum test for a specific region, for comparing the groups. The CpG sites with an adjusted p-value below 0.05 and a mean methylation change exceeding |0.1| were identified as differentially methylated. The resulting hypomethylated gene is related to the type I interferon pathway [111]. Here, the Wilcoxon rank-sum test, also referred to as the Mann-Whitney U test, is a widely-used nonparametric method for comparing two separate groups with a minimal assumption regarding data format, typically applied to medium to large datasets. Though widely used, this test is quite sensitive to outliners, the tied values (data points having the same values) and the small sample size [112]. Moreover, Mohammadnejad et al., 2021 used the generalized correlation coefficient (GCC) approach (Matie R package), linear mixed model (lme4 R package), and kinship model (kinship2 R package) together to identify 65 CpGs showing the association of DNA methylation with cognitive function ability of twin’s samples. The choice of these algorithms was guided by their appropriateness for analyzing DNA methylation data, accommodating both fixed and random effects, and capturing both linear and nonlinear correlations [102]. As discussed earlier, parametric linear regression models can be used to investigate the association between DNA methylation and symptom severity. A recent study done by Tang Y et al., 2024, used PANSS scores (positive, negative, general subscale, and total) and covariates (sex, age, education level, and cell type composition) to build linear regression models after using DMPfinder, then testing association methylation probes link to treatment response, considering antipsychotic details and baseline PANSS total score [113]. This algorithm has a few limitations: it assumes that observations are independent, is sensitive to outliers, and tends to underfit. A similar kind of study involves the derivation of DMRs/DMGs by using *DMRcate* (offered by ChAMP) and *Limma* to calculate methylation scores, distinguishing active Psoriasis from remission samples. It linked DMPs to disease activity and aided in the computation of methylation score [114].

Lastly, validating the methylation extent of derived hub genes obtained from gene expression analysis is essential for establishing their potential as epigenetic biomarkers in certain studies. The majority of the articles utilize web-based tools for validation. For instance, Luo D et al., 2022 used the MEXPRESS web-based tool to visualize the relationship between gene expression and methylation extend in hub genes related to Colorectal cancer [115]. Tong Lin et al., 2021 employed DNMIVD, SurvivalMeth, and MethServ tools for estimating the correlation between the expression level and the methylation density of DEGs, estimating the global methylation, and finding an association between the methylation level of CpG sites of the DEGs and overall survival of hepatocellular carcinoma patients, respectively [116]. Another study used the MethHC database to explore the candidate hub gene derived from lung adenocarcinoma patient samples, whose methylation level is negatively correlated with gene expression, affecting the normal functioning of the diseased pathway [117]. Furthermore, Shijian et al., 2022 found an association of the hub gene expression with the abnormal methylated data of the disease by using DiseaseMeth 2.0 database [118]. Further validation can involve the application of Single-Sample Gene Set Enrichment Analysis (ssGSEA) which can specify the enrichment score of the hub genes. This analysis is pursued to investigate the immune infiltration landscape of the sample which can act as a measure for disease progression [100]. The survival analysis validates hub genes by assessing their impact on patient survival and the application of the TIMER database analyzes the role and correlation of immune cells with the progression of the disease [99]. Table 1 encompasses a comprehensive analysis of recent publications in DNA methylation-based studies over the past five years focusing majorly on the identification of DMRs/DMPs, giving a clear takeaway of result analysis done in this section.

#### 3.3.2 Algorithmic approaches for identifying methylation states in single-condition

The genome is categorized into distinct methylation patterns subjected to a single condition, including Unmethylated Regions (UMRs), Low Methylated Regions (LMRs), Fully Methylated Regions (FMRs), and DNA methylation valleys (DMVs). In recent times, several R packages aiming the statistical and AI-based approaches have been used for methylation segmentation, extending beyond array data analysis. The use of HMM segmentation is the most popular method for locating regions with CpGs in comparable methylation states. The development of methPipe by Smith Lab uses a two-state HMM to identify hypo- and hypermethylated regions and to detect allele-specific methylated regions with consecutive methylation values around 50% [124], [125]. Malonzo et al., 2023 developed LuxHMM, a probabilistic method and software that uses a hidden Markov model and a Bayesian regression model to segment and infer differential methylation of regions in bisulfite sequencing data [126]. In the same year, the MethyLasso approach was developed for whole genome datasets enabling the independent analysis of the data belonging to different conditions of patients and integrating replicates to identify LMRs, UMRs, DMVs, and PMDs by segmenting DNA methylation levels and variation [127]. In recent studies related to cancer, genetic alternation such as Copy-number variations (CNVs) may impact tumor classification and therapeutic decisions. Therefore, the R package conumee 2.0, designed for such studies, combines tangent normalization, genomic binning heuristic, and weighted circular binary segmentation to analyze Copy-number variations (CNVs) using DNA methylation arrays [128]. The AI algorithms application can increase the efficiency and the accuracy of the methylation-based classification/segmentation methods. Towards this goal, Liu Y. et al., 2023, propose methylClass, an R package that offers an eSVM (ensemble-based support vector machine) model for methylation data classification, improving accuracy and overcoming time-consuming traditional SVM methods. The package also includes novel feature selection methods and multi-omics integration methods such as the Single-Cell Manifold Preserving Feature Selection (SCMER) method, the JV method performing joint tSNE and UMAP embedding, and Multi-Omics Graph cOnvolutional NETworks (MOGONET) [129].

#### 3.3.3 Application of ML/DL for methylation array data analysis

Progressively, cutting-edge ML/DL techniques have broadened the scope for identifying DMRs/DMCs, offering increased flexibility in detecting patterns within complex, high-dimensional data, and yielding numerous potential biomarkers and drug targets [130]. This aims to identify and characterize genomic regions as methylation features that are later subjected to the construction of prognostic or classification models for finding the prognostic behaviour and predictive accuracy of the disease. Numerous methods for feature selection have been utilized to discover a variety of methylation features that exhibit strong associations with survival outcomes and also can be used as dimensionality reduction step. This simplifies the development of the prognostic model, which estimates the risk of disease onset and progression, facilitates the stratification of the population into high and low-risk groups, and assesses the overall survival efficacy of the identified key methylation biomarker. Thus, the construction of classifiers is preferred over the use of the statistical algorithm for studying the diagnostic and prognostic behaviour of disease biomarkers. Therefore, this section deals with the review of the research methodologies followed for processing methylation microarray data using machine learning/deep learning approaches.

For instance, Zheng et al., 2020, demonstrated the Deep neural network (DNN) model's ability to classify cancer origin and predict cancer cell types using 10360 CpG sites from 7339 patients with 18 cancer origins. These 10360 CpG sites were filtered from ANOVA and Tukey’s honest significance difference tests [131]. Deep learning algorithms coupled with feature selection methods can reduce data complexity and increase the prediction accuracy of the model. Therefore, a study done by Gomes et al., 2022, introduced an approach using a Deep neural network (DNN) classifier model with the feature selection method as a Wilcoxon rank-sum test to identify the top 685 CpG markers in 27 K array and Random Forest algorithm to identify the top 1572 CpG markers in 450 K array. Later, the selected CpG markers are used as an input in the DNN model deducing 7 prognostic overlapping genes between 27 K and 450 K array [132]. The use of the Wilcoxon rank-sum test as a feature selection method and deep learning models can identify key methylation features, enhance predictive accuracy, maintain scalability, and handle non-normal data effectively [75]. Another study conducted by Zhang G. et al., 2021, considered the use of both DNA Methylation and Gene Expression Datasets for selecting the differentially expressed genes by applying mutual information (MI), along with fold change (FC), T-test, and false discover rate (FDR) test, as feature selection steps. The selected features are imported into the DNN classifier model to measure its classification ability and to identify biomarkers for gastric cancer [133]. The interpretation of using a broader range of statistical methods can potentially improve model robustness but at the same time can increase the risk of overfitting with too many features.

Despite extensive research, the clinical implications of DNA methylation in disease prognosis, tumor classification, and survival outcomes remain unclear. There are various methodologies followed by researchers showing the systematic assessments of DNA methylation’s impact on overall survival outcomes of patients, with or without the use of feature selection methods. One such way is to develop a prognostic prediction model that integrates various differential methylation sites derived from high-throughput microarray assay data. For instance, Liu Y et al., 2021 reported an approach for identifying specific CpG sites as methylation features by combining data from Epigenome-Wide Association Study (EWAS) using CpGassoc R package and methylation BeadChip assays data processing using *minfi*. The selected features are subsequently fed into a Support Vector Machine (SVM) classifier for model training. This model used the β-values of CpG sites derived from EWAS, as the predictor variable for predicting the diagnosis of Gestational Diabetes Mellitus [134]. While SVMs offer advantages such as handling nonlinear relationships, robustness in high-dimensional spaces, and effectiveness with small sample sizes, they do have limitations [135]. These include the inability of the *Minfi* package to capture all relevant biological variability and the challenge of interpreting SVM models [136]. Progressively, a study done by Shu C et. al., 2021, analyzed HIV-positive veterans by using an ensemble model (including Random Forest (RF), GLMNET, SVM, and k-nearest neighbours (k-NN)) based on 393 CpG sites derived from feature selection mechanism, to predict mortality risk [137]. The use of ensemble methods can lower the variance of the models by bagging and subsampling, and increase the model robustness to enhance the diagnosis and prediction performance [138]. Additionally, various studies employ the Cox proportional hazard model to examine how various external factors may affect patient survival outcomes. For instance, Xu et al., 2022, demonstrated that the combination of univariate and multivariate Cox models effectively identifies prognosis-related CpG sites. These sites were then categorized into subgroups through consensus clustering to construct prognostic models for lung adenocarcinoma [139]. Also, Guan W et al., 2022 employed the Wilcoxon rank-sum test to assess differences in methylation β-values between thymoma and thymic carcinoma and proposed an approach for identifying candidate methylation sites with potential prognostic impact. For validation purposes, they used univariate Cox regression to find methylation sites closely related to recurrence-free survival (RFS) in thymic epithelial tumors (TETs). However, multivariable Cox regression, incorporating forwarding selection for covariates, revealed that only a few characteristic features remained independent prognostic factors for RFS in TETs [140]. Using a similar Cox regression model approach, Wu et al., 2021 identified 166 independent prognosis-related CpG sites which were subjected to the consensus clustering method for finding a cluster showing the highest methylation sites associated with the risk scores. Overall, this model can subdivide the cohort into high-risk or low-risk cancer groups suggesting the poor prognosis of the hypermethylated group [141]. Meanwhile, Peng et al., 2021 employed a beta-mixture model (via the Methylmix package) for the identification of MDGs. Subsequently, they constructed a prognostic gene panel by combining Cox regression with the least absolute shrinkage and selection operator (LASSO) regularization methods [142]. Additionally, Wang et al., 2021 acquired Cox proportional hazard models to select the 11-methylation marker related to the Overall survival (OS) of patients from 485577 methylation sites (samples), being subjected to nomogram construction. This nomogram significantly enhances the predictive capability of the existing predictor for the OS of patients having stage I-II lung adenocarcinoma [143]. Yin et al., 2021 also mentioned the formation of a nomogram model with clinical features and prognostic risk model output, that involves factors to provide accurate prognostic estimates for patients' long-term survival outcomes. Such prognostic risk model construction was based on the 111 differentially methylated CpG sites derived from the preliminary steps of clustering into molecular subgroups and DNA methylation analysis on pancreatic samples [86]. Cox regression models offer detailed insights into DNA methylation's impact on patient survival outcomes, yet they struggle with high-dimensional data overfitting. Pre-applying feature selection enhances their performance, making it comparable to the approaches showing the integration of machine learning algorithms with feature selection [144].

Other sample classification strategies for risk stratification involve the use of multivariate filter-based methods such as PLS-DA (Partial Least-Squares Discriminant Analysis), LDA (linear discriminant analysis), CFS (Correlation-based Feature Selection), and multiple regression analysis such as OPLS-DA (Orthogonal Projections to Latent Structures Discriminant Analysis), Sparse Partial Least Squares Discriminant Analysis (sPLSDA). These techniques aid in creating a prognostic classification panel, comprehensively exploring biomarkers and risk factors for disease recurrence [145]. PLS-DA is a supervised version of Principal Component Analysis and a multivariate dimensionality-reduction method that achieves feature selection and classification model building for identifying biomarkers and further stratification of the samples into different risk groups based on their methylation patterns [146]. An advanced iteration of PLS-DA, termed OPLS-DA is gaining popularity for its ability to create decipherable models by dividing variance into predictive and noise-based parts, making it easier to create models compared to its previous version. For instance, Agarwal P et al.,2022 employed this classification method to identify the metabolites and DMRs with utmost importance which was followed by the application of the cross-validation method to avoid overfitting [147]. Later, the PLS/OPLS-DA model calculates VIP (variable influence of projection) for metabolites based on predictive components, with VIP> 1 or 1.5 thresholds used for further analysis using linear regression models [146], [147]. While effective for DNA methylation data analysis, this computationally intensive method operates on assumptions like linearity and homoscedasticity, potentially leading to biased outcomes if not met in practice. Another supervised method used by Marie-Claire et al., 2020 is the sPLS-DA algorithm that merges the features selection ability of PLS-DA with the predictive strength of logistic regression, to distinguish between lithium excellent-responders (LiERs) and non-responders (LiNRs) in patients with bipolar disorder type 1. Using sPLS-DA, they identified DMRs by combining Partial Least Squares and Lasso penalization, determining optimal DMRs via ROC curve analysis, and assessing feature selection stability with bootstrap samples. LOOCV evaluated model performance, enabling treatment response prediction based on methylation profiles [148]. While sPLS-DA is effective for binary classification, its direct applicability to multi-class problems or other data types may be limited. Conversely, the CFS algorithm selects attributes based on gene usefulness for prediction, minimizing inter-correlation among features to avoid redundancy. It evaluates subsets considering predictive ability and correlation, offering heuristic merit for feature subsets, unlike methods focusing solely on individual features. This approach allows the designing of heuristic functions to minimize costs towards the goal [149]. However, in a 2023 study, researchers demonstrated that mRMR and F-score do better feature selection for Alzheimer’s disease prediction using gene expression data, surpassing the performance of Chi-Square and CFS filters [150]. In the same year, Sharif Rahmani E et al. introduced MBMethPred, an AI-based computational framework utilizing a linear model (LDA) for subgroup classification with 763 medulloblastoma samples. This framework uses LDA for feature selection and ANN for capturing intricate nonlinear relationships between variables, achieving classification accuracy exceeding 96% and utilizing 399 CpGs as prediction biomarkers [151].

To enhance the predictive power and classification accuracy of the developed model for the patients’ samples, many studies have employed complex feature selection algorithms rather than traditional methods. This can provide an extensive understanding of the methylation features and their role in the disease. For instance, Wu J et al., 2017, introduced a three-step feature selection method including minimum redundancy maximum relevance (mRMR-wrapper method) relying on mutual information theory, differential methylation analysis (filter method), and another wrapper method based on a genetic algorithm. This was integrated with the classification model formation using RF based on the selected candidate probe, classifying the samples into normal Lymph node (LN), negative LN metastasis (LN-), and positive LN metastasis (LN+) used for obtaining a biomarker for predicting lymph node metastasis of stomach cancer. Therefore, the mRMR feature selection method effectively reduced the risk of overfitting in the prediction task but may require fine-tuning of the parameters that can be considered as a limitation [152]. As previously discussed, Wang et al., 2021 collectively used precursive information of the differentially methylated sites between normal and periodontitis samples and co-expression modules of CpGs (derived from WGCNA analysis) for the construction of a Support Vector Machine (SVM) classification model with a prediction accuracy of 95.5%. The classifier’s high performance on both the training and external datasets indicated that the derived genes had a strong ability to classify periodontitis and provide biological context to features [93]. Instead of integrating the feature selection methods, Adeoye et al., 2022 compared the ANOVA, mRMR, and LASSO (Least Absolute Shrinkage and Selection Operator) mechanism as feature selection techniques, for finding DMCs/DMRs as predictive features for machine learning models (SVM, Random Forest, and ExtraTrees) proving that the 11 DMRs selected through LASSO for the linear SVM model had the ideal AUC, recall, specificity, and calibration for OSCC detection [153]. From a technical viewpoint, Zhuang, J. et al., 2012 already stated that the construction of a classification model using the Elastic Net and Support Vector Machine (SVM) outperforms competing methods like LASSO and supervised principal components analysis (SPCA) [75]. Still, recent research has shown the wide exploitation of LASSO as a feature selection mechanism followed by the construction of SVM, random forest (RF), and Deep learning (DL) classification models showing high accuracy for identifying DMCs/DMRs associated with specific diseases, serving as both predictive and diagnostic features. Despite the preferable usage of SVM and Naïve Bayes, random forest (RF) outperformed most algorithms. It handled complex feature interactions and provided high accuracy, stability, and predictive power [144], [154]. Considering this robust classification done by RF models, Tu et al., 2022 effectively used the LASSO as a feature selection mechanism and RF model construction for the selection of the significant methylation features, allowing the accurate prediction of the samples for the occurrence of cervical cancer and supporting the stratification of the patients’ samples into low-risk and high-risk groups [155]. Also, Principal Component Analysis (PCA) is often chosen as the feature selection algorithm for enhancing the model performance. A study done by Nguyen et al., 2022 approved the use of the PCA method for finding principal components and employing ML algorithms such as Deep learning (DL), Support Vector Machine (SVM), and Random Forest (RF) for model construction, resulting in the identification of biomarker showing the high biological processes prediction of samples and the disease-oriented with it. This work states the better performance of the Deep learning model as compared to other models [156]. Due to its limitations such as reliability on original variable data, linear relationship constraints, and oversight of data's multivariate aspects, PCA is unsuitable for complex data analysis. In contrast, methods like t-SNE and UMAP are preferable for their ability to handle non-linear interactions and complexities.

Instead of feature selection methods, numerous studies use feature ranking methods which can reduce the dimensionality of the data, remove irrelevant or redundant features, and enhance the interpretability and generalization of the model [157]. For instance, some recent studies were done by Jian et al., 2022 and Ren et al., 2022, filtered the methylation probes by applying the Boruta algorithm, ranked the features using MCFS, LightGBM, and LASSO, and incremental feature selection (IFS) with decision tree and random forest algorithms for creating six classification models. This helped to extract essential methylation features; construct efficient classifiers and classification subtyping rules for anal carcinoma, cervical carcinoma, and sarcoma patients’ samples [158], [159]. A similar approach of feature ranking methods has been used to create high-performance classification models that can identify methylation sites and decision rules for COVID-19, lymphoblastic leukemia, and non-Hodgkin’s lymphoma. Therefore, we can state that it can be a better approach to adopt for finding relevant methylation signatures and sample classification rules created by ML classifiers. Some of its limitations include the stringent selection criteria for features, leading to the exclusion of relevant features [160], [161], [162]. A comprehensive overview of all the reviewed articles unveils the classification models using DMRs with a clear tabulated analysis input, the algorithm followed for model formation, and the output of the study is given in Table 2.

#### 3.3.4 Annotation and Visualization of DMRs

Annotation is a crucial step in evaluating the biological relevance and functional significance of DMRs. This process involves enriching functional annotations within genomic regions that display distinct DNA methylation patterns. Various tools and databases are commonly used for functional enrichment analysis, including GSVA, DAVID, GO, and KEGG pathway analysis. GSVA (Gene Set Variation Analysis) is an unsupervised computational analysis that identifies diseased molecular pathways associated with DMS [118]. DAVID database is a reputable choice for researchers seeking integrative and systematic gene annotation. It provides information on biological pathways, protein networks, and gene ontology terms. GO and KEGG pathway analyses aid in identifying signature disease-related genes. GO annotations classify enriched pathways into cellular components, biological processes, and molecular function categories, while KEGG analysis uncovers relevant molecular and metabolic pathways and interacting networks in the context of the disease [87], [90], [91], [92], [94], [97], [164]. To annotate genes closely associated with methylated sites, the study utilized the GRCh38 annotation file from the GENCODE project [86]. The STRING database was used to explore functional proteins and protein-protein interactions (PPI) related to hub genes, contributing to an improved understanding of disease biomarkers [87], [91], [92]. Subsequently, Cytoscape was employed to visualize the intricate network, and its integrated application, Cytohubba, was utilized to identify the most significant hub genes within the PPI network [105], [165]. Various studies utilize a range of tools and online databases to analyze hub genes, establishing them as potential biomarkers for disease. Databases such as GENEMANIA and miRWalk are utilized for pinpointing genes associated with a predefined list of genes and for mapping out gene-miRNA interaction networks, respectively [97,[116], [164].

Conventional approaches to gene set testing may generate biased P-values owing to variations in gene lengths. For Illumina array-profiled DNA methylation data, methods adjusting for the number of CpGs, rather than gene length, are imperative. MethylGSA resolves this concern by facilitating gene set testing with adjustments for length biases. This enables the discovery of enriched pathways extracted from prominent databases like Gene Ontology, KEGG, and Reactome [166]. Notably, the number of CpGs linked to each gene on the 450 K array varies widely, potentially biasing gene set analysis. This calls for the application of the gometh function offered by the missMethyl Bioconductor package by adjusting for the number of CpGs associated with each gene. The input taken is a vector of significant CpGs followed by a hypergeometric test, considering the CpG site density per gene on the 450 K/EPIC arrays [167]. Moreover, the same package offers GSAmeth function which is designed to assess if there’s a statistically significant concentration of differentially methylated CpG sites within gene sets predefined by the researcher. This method systematically evaluates the presence of methylation changes across these gene sets to understand their potential biological impact [168]. Outperforming the ways focussing on identifying individual genes that exhibit differences between two states of interest, the introduction of Gene set enrichment analysis (GSEA) analyzes the expression data at the level of gene sets. This offers several advantages such as enhanced interpretation by identifying pathways and processes, greater reproducibility and interpretability, an enhanced signal-to-noise ratio, and detects subtle changes in genes within highly correlated sets [169]. Additionally, an advanced iteration of GSEA, known as ebGSEA, was introduced to address the issue of differential probe representation on Illumina Infinium DNA methylation bead chips. This method prioritizes genes over CpGs, ranking them based on overall differential methylation levels using all corresponding probes. It offers improved sensitivity and specificity compared to existing methods for EWAS data analysis [170]. Furthermore, GSEA can be adapted for cross-species studies through domain adaptation. This approach known as CROSS-species gene set enrichment problem (XGSEP) allows for the analysis of gene expression measured under the same phenotype of different species, which is particularly useful when direct experiments on humans are risky and are instead substituted by model organisms like mice. The XGSEP method is structured into three stages: GSEA, domain adaptation, and regression [171]. Furthermore, GSEA software has been updated to support RNA-seq datasets and single-sample analysis (ssGSEA), expanding its applicability in various biological states and phenotypes [172]. These adaptations enhance the utility of GSEA in modern biological research, allowing for more comprehensive and versatile analyses.

Visualization of DMS aids in detecting inaccuracy in results, identifying the features and exploring patterns that are not detectable in the tabular outputs, and comprehensible investigation of the biological processes related to the genomic data allowing the researcher to hypothesize the research outcome [173]. Heatmaps visually represent color variations to display different variables, including hypermethylated and hypomethylated CpG sites while scatter plots illustrate the association between variables such as methylation level and gene expression or methylation level at specific CpG sites [174], [175], [176]. Volcano plots are a form of scatter plot that visualize and identify DMRs between studied groups [111], [177]. Furthermore, box plot visual representation allows the user to correlate the relationship between sample tissue and methylation value [178], [179], [180]. A violin plot is an amalgamation of a box plot and kernel density distribution, to display CHH/CG/CGH methylation levels in specific DMRs [181], [182], [183]. What’s more*, UCSC Genome Browser Home* is a genome browser available for visualizing particular genome annotations, along with analyzing and comparing the genomic datasets [184]. The *Ensemble* database offers reliable genome annotations and tracks gene evolution across species. It also allows for the incorporation of related biological data mapped onto features derived from the genome [185]. Also, some of the web-based applications such as MethSurv, are designed with user-friendly efficiency providing the visualization of the CpG sites, functional analysis, graphical parameters, and survival correlations using the Cox proportional-hazards models [186], [187], [188].

## 4 Discussion

DNA methylation is one of the earliest and most significant heritable events among the epigenetic marks of the genome associated with gene regulation, as well as developmental and progressive events of underlying disease [157]. The genome-wide methylation profiling analysis has attained widespread popularity for the identification of epigenetic biomarkers (episignatures) acting as the predictive tool for clinical studies. Also, this analysis makes way for the classification of diseases based on molecular subtyping, guiding treatment choices, and ultimately managing overall patients’ life expectancy [181]. There are a variety of computational tools and algorithms available for the processing and analysis of DNA methylation profiling data, detailed in several review articles [15], [71], [89]. Consequently, this review provides a comprehensive and consolidated overview of diverse aspects of array-based methylation data analysis within a single resource, highlighting the trending methodologies or workflows followed by the researchers for finding the methylated dysregulated sites. In this study, we have outlined existing tools and workflows, evaluated their primary strengths and limitations, and proposed a selection of algorithms that we believe currently offer the most effective approach for analyzing DNA methylation microarray data.

In terms of databases, public repository data has been identified as the preferred choice among academics and practitioners for analyzing DNA methylation array data. However, despite the frequent limitations of current datasets, such as imbalanced data and missing data, many researchers also rely on additional data acquired from hospitals and clinics. To assist future researchers and practitioners interested in analyzing DNA methylation array data, we’ve curated a list of commonly referenced datasets, detailing their origins, sample sizes, representativeness, and selection criteria in Supplementary Table II. Additionally, Supplementary Table I contains links to and descriptions of public repositories.

The choice of pre-processing method is of utmost importance as it can drastically affect the between-sample variability and the results of the analysis [189]. Most of the workflows recommend the use of the *Minfi* package for pre-processing the array data, and other frequently used algorithms with details are listed in Supplementary Tables III and IV. The identification of global changes can be facilitated by visually inspecting methylation data, which can be achieved through various clustering methods outlined in referenced studies, such as PCA, hierarchical clustering, K-means clustering, and consensus clustering. We highly recommend employing consensus clustering (model-based clustering method) and recursively partitioned algorithms, as they are effective for processing high-dimensional data. These methods form distinct methylation subtypes that help classify diseases, which are then analyzed by clinical and molecular traits. Additionally, the utilization of consensus clustering allows for representing consensus across multiple clustering algorithm runs and evaluating cluster stability with random restarts [88], [101].

The downstream analysis involves identifying differentially methylated regions across different biological conditions using tools like the *Limma* package and the ChAMP pipeline, which are known for their efficacy in array-based methylation data analysis. Renowned for its efficacy in gene discovery, *Limma* excels in differential expression analysis for methylation arrays, microarrays, and RNA-seq data. Also, ChAMP provides a comprehensive analysis, including batch effect correction, differential methylation, copy number variation adjustments, cell type heterogeneity management, network analysis, and an interactive GUI [24], [101]. We also suggest considering linear regression models coupled with deep learning algorithms to elucidate the intricate associations between DNA methylation signatures and gene expression data, offering insights into clinical variables [190]. Additionally, statistical Cox regression models are highly recommended for effectively identifying prognostic CpG sites associated with disease, offering superior estimations of survival probabilities and cumulative hazards compared to the Kaplan-Meier function [191]. Furthermore, we advocate for the use of analytical tools like MEXPRESS, DNMIVD, SurvivalMeth, MethHC, DiseaseMeth 2.0 database, ssGSEA, and TIMER to validate biomarker gene methylation and explore the correlation between the gene expression and methylation levels, which are the crucial components of a comprehensive methylation analysis workflow.

Despite the complexities of disease mechanisms and symptoms, our review highlights the utility of ML/DL algorithms in enhancing the efficiency of disease diagnosis and prognosis. We support the utilization of Deep Neural Network (DNN) models, complemented by robust feature selection techniques for the identification of DNA methylation profiles into distinct regions based on observed methylation patterns. This approach is advantageous for capturing complex and non-linear patterns within high-dimensional datasets, thereby enhancing predictive accuracy. Additionally, the review commonly examines algorithms such as Support Vector Machines (SVM), which are particularly prominent, along with K-Nearest Neighbors (KNN), Random Forests (RF), Deep Learning (DL), and Decision Trees (DT), all of which are extensively employed in disease diagnosis research [192]. Generally, the selection of the feature selection methods observed in reference articles, comprised of Principal Component Analysis (PCA), Least Absolute Shrinkage and Selection Operator (LASSO), and sometimes more intricate approaches like Minimum Redundancy Maximum Relevance (mRMR) and feature ranking methods such as Monte Carlo Feature Selection (MCFS), Light Gradient Boosting Machine (LightGBM) and Incremental feature selection (IFS). The selection of these methods is influenced by sample size, the biological context of the study, computational limits, and the aim to enhance predictive accuracy.

### 4.1 The effective role of algorithms in the diagnosis of some closely related human diseases

**Fig. 3:**
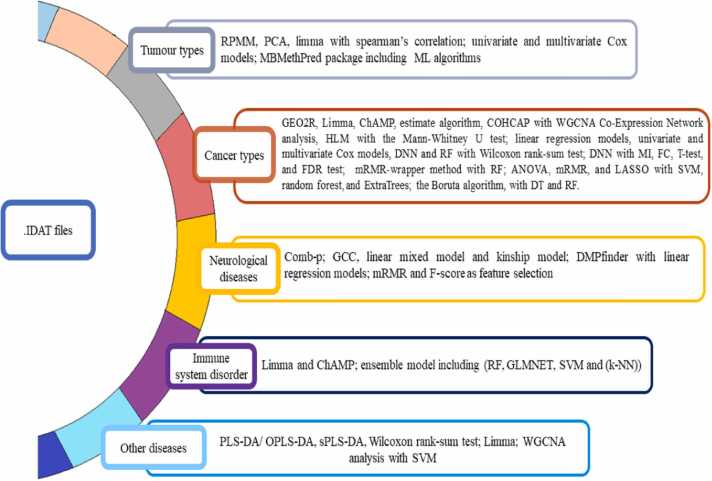
Figurative approach of diseases diagnosed by different effective algorithms of some related human diseases.

This paper provides a survey of different R packages, statistical algorithms, and machine learning techniques for the diagnosis of different diseases such as numerous cancer types, varying tumor types, atherosclerosis, dementia, diabetes, high blood pressure, periodontitis, Acute respiratory syndrome, Alzheimer’s, schizophrenia, Coronary artery disease, HIV, bipolar disease type I, and knee osteoarthritis. Analytical and computational pipelines used to analyze two similar diseases may differ according to the available data, disease characteristics, and the specific objectives of the analysis. The predominant focus of the reviewed literature pertains to various cancer types, highlighting the utilization of the *limma* or ChAMP pipeline in identifying diagnostic CpG sites. Additionally, these studies also employ WGCNA, HLM, and the Mann-Whitney U test to explore the biological function and measure the accurate estimates of the methylation differences. Moreover, in cancer research, univariate and multivariate Cox regression analyses are effectively used to identify prognostic factors for developing nanogram to predict patient survival [193]. For the diagnosis of cancer-related diseases, other recommended machine learning (ML) and deep learning (DL) algorithms include DNN, CNN, ANN, RF, and SVM classification models. These models are often enhanced by feature selection algorithms like MCFS, LightGBM, IFS, ANOVA, mRMR, and LASSO to improve diagnostic accuracy [194]. Similarly for tumor diagnosis, the MBMethPred package, integrating ML and neural network models, is suitable for subgroup classification whereas univariate and multivariate Cox models have demonstrated satisfactory prognostic accuracy. Clustering methods like RPMM, PCA, tSNE, consensus clustering, NMF, and hierarchical clustering are recommended for cancer diagnosis, with *consensus clustering* offering robust predictions and *K-means* excelling in high variance scenarios with identical centroids [195], [196]. For neurological disorders, algorithms such as *Comb-p* and DMPfinder are effective in identifying DMRs as diagnostic biomarkers, often used in conjunction with linear regression models and feature selection methods like mRMR and F-score. In the realm of immunological diseases, an ensemble approach combining RF, GLMNET, SVM, and k-NN is recommended for robust diagnostic modeling, complementing traditional methods. The figurative approach of diseases diagnosed by the methodological framework (including all packages/algorithms/ ML models) is shown in Fig. 3.

### 4.2 The effective role of optimal algorithms in the diagnosis of specific diseases

ML predictive tools enable proactive disease diagnosis and risk evaluation, often before symptoms emerge. Breast cancer, being among the most frequently diagnosed malignancies, has witnessed a surge in research efforts. This has particularly been directed towards the application of ML algorithms to facilitate early detection. So, the utilization of ML algorithms such as DNN, LR, RF, SVM, KNN, and DT is extensively recognized in breast cancer research. Some of the traditional methods including Cox regression models, HLM, differential analysis, Mann Whitney U test, t-test, and ANOVA, continue to be pivotal for analyzing the diagnostic patterns of breast cancer [197], [198]. Analyzing microarray datasets with ML/DL algorithms can help pinpoint key protein biomarkers for early pancreatic cancer detection. Notably, Cox regression models, SVM classifiers utilizing Recursive Feature Elimination (RFE), and Artificial Neural Network (ANN) methodologies are among the most effective for this diagnosis [199]. In the realm of oncology, XGboost, and deep learning models, particularly CNN and DNN models, have demonstrated high accuracy in the early diagnosis of Esophageal cancer [200]. Moreover, studies support the effective incorporation of WGCNA for gaining biological insight into the disease. In cervical cancer research, integration of traditional and machine learning algorithms such as Differential Methylation Analysis (using *limma*), LASSO, and Boruta feature selection, coupled with DT and RF classifier models, is a prevalent way for identifying diagnostic biomarkers [201]. DL models, Logistic Regression, and Cox Regression models have been thoroughly explored for integration into Computer-Aided Detection (CAD) systems for automated detection and classification of lung cancer [202]. In the context of Alzheimer’s disease, the Comb-p algorithm for DMR analysis, while Singular Value Decomposition (SVD), PCA, mRMR, and F-score have been utilized for feature selection, with a CNN serving as a classifier to enhance disease prediction [203].

### 4.3 Limitations and future prospects

Inevitably, we also acknowledge certain innate limitations associated with the review which cannot be covered due to time and scope of exploration constraints. Firstly, several algorithms give importance only to differentially methylated CpG islands, overlooking the significance of distal regulatory elements regions in the genome which are believed to provide crucial support for biomarker investigation. Second, correlation analysis of the risk score obtained in the diagnostic model with that of the clinical characteristics, generation of copy number data, single-cell methylation analysis, and tumor microenvironment are the unexplored sections in the field of epigenetic research that are not held accountable in this review. Additionally, the discussion of computational pipelines and algorithms specific to sequence-based methylation data falls outside the scope of this review. Looking ahead, there is ample opportunity to broaden this study to include multi-omics approaches, which would integrate DNA methylation studies with other epigenomic data, thereby enriching the avenues for disease treatment, diagnosis, and prognosis.

As we delve deeper, beyond the scope of methodologies covered within the review, there emerges a range of additional statistical algorithms deserving of attention. Each of these algorithms carries the potential to reshape and elevate epigenetic research by offering fresh perspectives and insight. Such as Graph theory-based analysis, capable of elucidating biological pathways through the integration of DNA methylation, gene expression datasets, and other omics data, has been incorporated in classification studies and has proven to be superior than other state-of-the-art algorithms. This graph-based approach can also deal with the complexity of the microarray data and its large number of features (genes) while annotating with samples [204]. Additionally, Bayesian approaches, hold the potential to address inter-cellular methylation heterogeneity and counteract the sparsity often observed in methylation data. Furthermore, the interdependency of the methylation functionality and its changes over the change in the environmental conditions can be depicted with the application of the longitudinal analysis of methylation data for predicting the CpG sites with different stabilities, states, and functionality [205]. Building upon the foundation of these innovative algorithms and the methodologies outlined in this review, we are equipped with a robust resource that can guide the selection of effective protocols for DNA methylation array data processing and analysis. Certainly, DNA methylation is a stable and reliable biomarker for disease diagnosis, as it does not change as easily as RNA or protein levels. Despite this, the widespread clinical integration of most candidate genes into molecular diagnostics remains distant. The general limitation faced by the computational algorithms is concerned with complex data, availability of variability in datasets compared to samples, the non-linear association of data, and the validation method of the algorithms for overcoming the error rate. Such limitations can influence the accuracy of the analysis and subsequently impact the identification of effective epigenetic biomarkers. To overcome these limitations, a promising approach should involve the integration of existing workflows into flexible algorithms/pipelines which can improve the detection and annotation of methylated sites, handle sample variability, and identify subtle genomic changes.

In the future, we believe that the field of epigenetic research can be uplifted by the utilization of deep learning techniques that can significantly enhance predictive modeling by effectively capturing intricate features linked to aging, cell types, and disease progression. As a result, they hold the potential to offer valuable diagnostic and prognostic clinical outcomes. Furthermore, the development of flexible algorithms that can segment the genomic regions with different methylation patterns, such as silencing certain disease-related genes (e.g., tumor suppressor genes in cancer) in some stretches of length, is necessary. Due to the advantageous nature of computational methods to harness heterogeneous data across several dimensions of biological variation, several developments have been made so far for predicting better clinical outcomes and a comprehensive view of the regulatory landscapes. One such development includes simultaneous profiling of histone modification and DNA methylation from a single DNA molecule using the nanopore sequencing technique [206]. The derived ONT (Oxford Nanopore Technology) data from the nanopore sequencing technique requires the evolution of robust and user-friendly bioinformatics software, providing cloud storage and real-time analysis [207]. Further, the investigation of the epigenetic markers for finding reliable correlations with the living phenotype leads to the enhancement of epigenetic editing methods (including established tools like CRISPR-Cas9) which simultaneously brings the computational framework into consideration for developing machine-learning-based model that can incorporate multi-dimensional data features, genetic variation data, and chromatin accessibility data for predicting off-target as well as on-target effects [208], [209]. Additionally, existing research has integrated linear regression algorithms with methylation profiling to explore correlations between environmental factors or exposome and changes in DNA methylation data at least at ‘metastable epialleles’ denoting the developmental environment [210]. This explores the ways of dealing with limitations of existing computational algorithms such as produced bias due to accounted relatedness of samples and unreliable certainty of the statistical tests if there is a significant difference in the number of cases and controls. Moreover, the requirement for robust bioinformatics pipeline/algorithms development may increase due to the growing production of whole-genome sequencing (NGS) data in the position of supporting the analysis of methylation profiling data for biomarker and SNP estimation. Because it deals with the functional ramifications of uncommon DNA modifications, the presence of uncommon cell types, and intercellular heterogeneity, the integration of single-cell epigenomics techniques with transcriptome and epigenomic sequencing data will also necessitate a significant evolution of computational pipelines and strategies [211]. By disclosing specific molecular and genetic variations at the cellular as well as genomic level, the future outlooks discussed so far can improve research efforts in the creation of novel algorithms and workflows for enhancing the efficacy of targeted illness therapy. We strive to create novel and enhanced computational approaches that will advance personalized healthcare and precision epigenetic therapy.

## 5 Conclusion

The diversity of measurable epigenetic markers enables the use of epigenetic events as early indicators of human disorders and provides mechanistic clues to disease etiology. The quest to identify epigenetic biomarkers is propelling advancements in the medical field, leading toward a paradigm where personalized treatment strategies significantly enhance patient care, diagnostic ability, and prognostic accuracy. This necessitates a complexity of methods for array processing and analysis where optimized computational algorithms adeptly address crucial aspects like disease detection accuracy and effective treatment. It's essential to establish a protocol of best practices for the algorithms and packages mentioned earlier in the review. This ensures that research outcomes are of the highest quality and stay relevant to diverse research hypotheses. The recommended methodologies and algorithms are decided based on the computationally intensive nature of the resource’s availability, the user-friendly nature of algorithms, and better validation of results. The study's goal is to examine the range and efficacy of various algorithms and workflows used in recent research, with a focus on their accuracy in disease prediction through the identification of biomarkers, classification, clustering, and survival analysis. While designing a pipeline for DNA methylation array analysis, it's crucial to incorporate all the major processing and analysis steps previously outlined. An efficient computational workflow demonstrates the utilization of traditional statistical methods to categorize groups based on methylation profiles, followed by the application of machine learning (ML) techniques to enhance analysis and ensure prediction accuracy. For optimal results in DNA methylation data preprocessing and differential analysis, it is advisable to employ the *Minfi* package, complemented by traditional statistical methods such as the empirical Bayes approach *(limma)*, Bumphunter, and DMRcate for their proven effectiveness. Moreover, Consensus and recursive partitioning clustering algorithms excel in detecting methylation patterns and characterizing samples. The application of ML algorithms showing the highest accuracy for disease diagnosis, are DNN, RF, and SVM classification models. Furthermore, the synergy of Cox regression with logistic regression models in machine learning offers superior predictions for disease prognosis and patient survival rates. Despite the differences in frequency and performance metrics, this article depicts the promising potential of discussed algorithms and workflows in disease prediction. Through the proper use of the processing and analyzing methodologies outlined above, we hope that potential users will best harness the suitable possible outcomes from array-based data, leading to rapid advancement in human health and disease research.

## Reviewer disclosure

Peer reviewers on this manuscript have no relevant financial relationships or otherwise to disclose.

## Author statement

**K. Sahoo:** Data curation, Formal analysis, Investigation, Methodology, Validation, Writing – original draft. **V. Sundararajan:** Conceptualization, Project administration, Supervision, Validation, Writing – review & editing*.* All authors confirm that they played an important role in this work and are accountable for the content. All authors have read and agreed with the journal's guidelines for authorship, and reviewed, and approved the final version of the manuscript.

## CRediT authorship contribution statement

**Vino Sundararajan:** Conceptualization, Project administration, Supervision, Validation, Writing – review & editing. **Karishma Sahoo:** Data curation, Formal analysis, Investigation, Methodology, Validation, Writing – original draft.

## Declaration of Competing Interest

The authors declare that they have no known competing financial interests or personal relationships that could have appeared to influence the work reported in this paper.

## References

[1] Sant K.E., Nahar M.S., Dolinoy D.C. (2012). DNA methylation screening and analysis. Methods Mol Biol.

[2] Galbraith K., Snuderl M. (2022). DNA methylation as a diagnostic tool. Acta Neuropathol Commun.

[3] Mattei A.L., Bailly N., Meissner A. (2022). DNA methylation: a historical perspective. Trends Genet.

[4] Kandi V., Vadakedath S. (2015). Effect of DNA methylation in various diseases and the probable protective role of nutrition: a mini-review. Cureus.

[5] Moore L.D., Le T., Fan G. (2013). DNA methylation and its basic function. Neuropsychopharmacology.

[6] DNA methylation and silencing of gene expression n.d. 10.1016/s1043-2760(00)00248-4 10754536

[7] Navarro A., Yin P., Monsivais D., Lin S.M., Du P., Wei J.J. (2012). Genome-wide DNA methylation indicates silencing of tumor suppressor genes in uterine leiomyoma. PLoS One.

[8] Kulis M., Esteller M. (2010). Chapter 2 - DNA methylation and cancer. Epigenetics Cancer Part A.

[9] O’Sullivan E., Goggins M. (2013). DNA methylation analysis in human cancer. Methods Mol Biol.

[10] Yamato G., Kawai T., Shiba N., Ikeda J., Hara Y., Ohki K. (2022). Genome-wide DNA methylation analysis in pediatric acute myeloid leukemia. Blood Adv.

[11] Mensah I.K., Norvil A.B., Alabdi L., McGovern S., Petell C.J., He M. (2021). Misregulation of the expression and activity of DNA methyltransferases in cancer. NAR Cancer.

[12] Rauluseviciute I., Drabløs F., Rye M.B. (2019). DNA methylation data by sequencing: Experimental approaches and recommendations for tools and pipelines for data analysis. Clin Epigenetics.

[13] Liu Q., Fang L., Yu G., Wang D., Xiao C.Le, Wang K. (2019). Detection of DNA base modifications by deep recurrent neural network on Oxford Nanopore sequencing data. Nat Commun.

[14] Carrizosa-Molina T., Casillas-Díaz N., Pérez-Nadador I., Vales-Villamarín C., López-Martínez M.Á., Riveiro-Álvarez R. (2023). Methylation analysis by targeted bisulfite sequencing in large for gestational age (LGA) newborns: the LARGAN cohort. Clin Epigenetics.

[15] Rauluseviciute I., Drabløs F., Rye M.B. (2019). DNA methylation data by sequencing: Experimental approaches and recommendations for tools and pipelines for data analysis. Clin Epigenetics.

[16] Gao Y., Zhao H., An K., Liu Z., Hai L., Li R. (2022). Whole‐genome bisulfite sequencing analysis of circulating tumour DNA for the detection and molecular classification of cancer. Clin Transl Med.

[17] Gorrie-Stone T.J., Smart M.C., Saffari A., Malki K., Hannon E., Burrage J. (2019). Bigmelon: Tools for analysing large DNA methylation datasets. Bioinformatics.

[18] Danese A., Richter M.L., Chaichoompu K., Fischer D.S., Theis F.J., Colomé-Tatché M. (2021). EpiScanpy: integrated single-cell epigenomic analysis. Nat Commun.

[19] Su S.Y., Lu I.H., Cheng W.C., Chung W.C., Chen P.Y., Ho J.M. (2020). EpiMOLAS: An intuitive web-based framework for genome-wide DNA methylation analysis. BMC Genom.

[20] Hu X., Tang L., Wang L., Wu F.X., Li M. (2020). MADA: a web service for analysing DNA methylation array data. BMC Bioinforma.

[21] Schönung M., Hess J., Bawidamann P., Stäble S., Hey J., Langstein J. (2021). AmpliconDesign–an interactive web server for the design of high-throughput targeted DNA methylation assays. Epigenetics.

[22] Warden C.D., Lee H., Tompkins J.D., Li X., Wang C., Riggs A.D. (2013). COHCAP: An integrative genomic pipeline for single-nucleotide resolution DNA methylation analysis. Nucleic Acids Res.

[23] Gra∼ Na O, Ló Pez-Ferná Ndez H, Fdez-Riverola F, Gonzá Lez Pisano D, Glez-Pe∼ Na D. Bicycle: a bioinformatics pipeline to analyze bisulfite sequencing data n.d. https://doi.org/10.1093/bioinformatics/btx778. 10.1093/bioinformatics/btx778 29211825

[24] Tian Y., Morris T.J., Webster A.P., Yang Z., Beck S., Feber A. (2017). ChAMP: Updated methylation analysis pipeline for Illumina BeadChips. Bioinformatics.

[25] Rakyan V.K., Down T.A., Balding D.J., Beck S. (2011). Epigenome-wide association studies for common human diseases. Nat Rev Genet.

[26] Rahmani E., Schweiger R., Rhead B., Criswell L.A., Barcellos L.F., Eskin E. (2019). Cell-type-specific resolution epigenetics without the need for cell sorting or single-cell biology. Nat Commun.

[27] Sun W., Bunn P., Jin C., Little P., Zhabotynsky V., Perou C.M. (2018). The association between copy number aberration, DNA methylation and gene expression in tumor samples. Nucleic Acids Res.

[28] Mariani M.P., Chen J.A., Zhang Z., Pike S.C., Salas L.A. (2022). MethylMasteR: a comparison and customization of methylation-based copy number variation calling software in cancers harboring large scale chromosomal deletions. Front Bioinforma.

[29] Zhou W., Triche T.J., Laird P.W., Shen H. (2018). SeSAMe: Reducing artifactual detection of DNA methylation by Infinium BeadChips in genomic deletions. Nucleic Acids Res.

[30] Cho S., Kim H.S., Zeiger M.A., Umbricht C.B., Cope L.M. (2019). Measuring DNA copy number variation using high-density methylation microarrays. J Comput Biol.

[31] Knoll M., Debus J., Abdollahi A. (2017). CnAnalysis450k: An R package for comparative analysis of 450k/EPIC Illumina methylation array derived copy number data. Bioinformatics.

[32] Yosifov D.Y., Bloehdorn J., Döhner H., Lichter P., Stilgenbauer S., Mertens D. (2020). DNA methylation of chronic lymphocytic leukemia with differential response to chemotherapy. Sci Data.

[33] Clough E., Barrett T. (2016). The gene expression omnibus database. Methods Mol Biol, Vol 1418, Hum Press Inc.

[34] Karolchik D., Hinrichs A.S., Kent W.J. (2009). The UCSC genome browser. Curr Protoc Bioinforma.

[35] Davis C.A., Hitz B.C., Sloan C.A., Chan E.T., Davidson J.M., Gabdank I. (2018). The encyclopedia of DNA elements (ENCODE): data portal update. Nucleic Acids Res.

[36] Yang R., Pfütze K., Zucknick M., Sutter C., Wappenschmidt B., Marme F. (2015). DNA methylation array analyses identified breast cancer-associated HYAL2 methylation in peripheral blood. Int J Cancer.

[37] Kurdyukov S., Bullock M. (2016). DNA methylation analysis: choosing the right method. Biology.

[38] Khodadadi E., Fahmideh L., Khodadadi E., Dao S., Yousefi M., Taghizadeh S. (2021). Current advances in DNA methylation analysis methods. Biomed Res Int.

[39] Leti F., Llaci L., Malenica I., DiStefano J.K. (2018). Methods for CPG methylation array profiling via bisulfite conversion. Methods Mol Biol, Vol 1706, Hum Press Inc.

[40] Arora I., Tollefsbol T.O. (2021). Computational methods and next-generation sequencing approaches to analyze epigenetics data: profiling of methods and applications. Methods.

[41] Sun Z., Baheti S., Middha S., Kanwar R., Zhang Y., Li X. (2012). SAAP-RRBS: Streamlined analysis and annotation pipeline for reduced representation bisulfite sequencing. Bioinformatics.

[42] Gong T., Borgard H., Zhang Z., Chen S., Gao Z., Deng Y. (2022). Analysis and performance assessment of the whole genome bisulfite sequencing data workflow: currently available tools and a practical guide to advance DNA methylation studies. Small Methods.

[43] Ben Maamar M., Sadler-Riggleman I., Beck D., Skinner M.K. (2021). Genome-wide mapping of DNA methylation 5mC by methylated DNA immunoprecipitation (MeDIP)-sequencing. Methods Mol Biol, Vol 2198, Hum Press Inc.

[44] Rodriguez B.A.T., Frankhouser D., Murphy M., Trimarchi M., Tam H.H., Curfman J. (2012). Methods for high-throughput MethylCap-Seq data analysis. BMC Genom.

[45] Ahn J., Heo S., Lee J., Bang D. (2021). Introduction to single-cell dna methylation profiling methods. Biomolecules.

[46] Wright M.L., Dozmorov M.G., Wolen A.R., Jackson-Cook C., Starkweather A.R., Lyon D.E. (2016). Establishing an analytic pipeline for genome-wide DNA methylation. Clin Epigenetics.

[47] Suman M., Dugué P.A., Wong E.M., Joo J.H.E., Hopper J.L., Nguyen-Dumont T. (2021). Association of variably methylated tumour DNA regions with overall survival for invasive lobular breast cancer. Clin Epigenetics.

[48] Hernandez Puente C.V., Hsu P.C., Rogers L.J., Jousheghany F., Siegel E., Kadlubar S.A. (2020). Association of DNA-methylation profiles with immune responses elicited in breast cancer patients immunized with a carbohydrate-mimicking peptide: a pilot study. Front Oncol.

[49] Fackler M.J., Cho S., Cope L., Gabrielson E., Visvanathan K., Wilsbach K. (2020). DNA methylation markers predict recurrence-free interval in triple-negative breast cancer. NPJ Breast Cancer.

[50] Parashar S., Cheishvili D., Mahmood N., Arakelian A., Tanvir I., Khan H.A. (2018). DNA methylation signatures of breast cancer in peripheral T-cells. BMC Cancer.

[51] Coyle K.M., Patrick Murphy J., Vidovic D., Vaghar-Kashani A., Dean C.A., Sultan M., et al. Breast cancer subtype dictates DNA methylation and ALDH1A3-mediated expression of tumor suppressor RARRES1. vol. 7. n.d. 10.18632/oncotarget.9858 PMC5190082 27286452

[52] Singhal S.K., Usmani N., Michiels S., Metzger-Filho O., Saini K.S., Kovalchuk O., et al. Towards understanding the breast cancer epigenome: a comparison of genome-wide DNA methylation and gene expression data. vol. 7. n.d. 10.18632/oncotarget.6503 PMC4823086 26657508

[53] Maksimovic J., Gordon L., Oshlack A. (2012). SWAN: subset-quantile within array normalization for illumina infinium HumanMethylation450 BeadChips. Genome Biol.

[54] Fortin J.P., Triche T.J., Hansen K.D. (2017). Preprocessing, normalization and integration of the Illumina HumanMethylationEPIC array with minfi. Bioinformatics.

[55] Yao S., Hu Q., Kerns S., Yan L., Onitilo A.A., Misleh J. (2019). Impact of chemotherapy for breast cancer on leukocyte DNA methylation landscape and cognitive function: a prospective study. Clin Epigenetics.

[56] Fortin J.-P., Labbe A., Lemire M., Zanke B.W., Hudson T.J., Fertig E.J. (2014). Functional normalization of 450k methylation array data improves replication in large cancer studies. Method Open Access.

[57] Aryee M.J., Jaffe A.E., Corrada-Bravo H., Ladd-Acosta C., Feinberg A.P., Hansen K.D. (2014). Minfi: a flexible and comprehensive bioconductor package for the analysis of Infinium DNA methylation microarrays. Bioinformatics.

[58] Liu J., Siegmund K.D. (2016). An evaluation of processing methods for HumanMethylation450 BeadChip data. BMC Genom.

[59] Niu L., Xu Z., Taylor J.A. (2016). RCP: a novel probe design bias correction method for Illumina Methylation BeadChip. Bioinforma, Vol 32, Oxf Univ Press.

[60] Teschendorff A.E., Marabita F., Lechner M., Bartlett T., Tegner J., Gomez-Cabrero D. (2013). A beta-mixture quantile normalization method for correcting probe design bias in Illumina Infinium 450 k DNA methylation data. Bioinformatics.

[61] Ambatipudi S., Horvath S., Perrier F., Cuenin C., Hernandez-Vargas H., Le Calvez-Kelm F. (2017). DNA methylome analysis identifies accelerated epigenetic ageing associated with postmenopausal breast cancer susceptibility. Eur J Cancer.

[62] Mehdi A., Cheishvili D., Arakelian A., Bismar T.A., Szyf M., Rabbani S.A. (2020). DNA methylation signatures of prostate cancer in peripheral T-cells. BMC Cancer.

[63] Xu C., Sun D., Wei C., Chang H. (2022). Bioinformatic analysis and experimental validation identified DNA methylation–related biomarkers and immune-cell infiltration of atherosclerosis. Front Genet.

[64] Houseman E.A., Accomando W.P., Koestler D.C., Christensen B.C., Marsit C.J., Nelson H.H., et al. DNA methylation arrays as surrogate measures of cell mixture distribution. 2012. 10.1186/1471-2105-13-86 PMC3532182 22568884

[65] Mouat J.S., Li S., Myint S.S., Laufer B.I., Lupo P.J., Schraw J.M., et al. Epigenomic signature of major congenital heart defects in newborns with Down syndrome n.d. 10.1101/2023.05.02.23289417. PMC10559462 37803336

[66] Song J., Kuan P.F. (2022). A systematic assessment of cell type deconvolution algorithms for DNA methylation data. Brief Bioinform.

[67] Package “minfi.” 2024.

[68] Distinct DNA Methylation Signatures in Neuroendocrine Tumors Specific for Primary Site and Inherited Predisposition n.d. 10.1210/clinem/dgaa477 PMC7456345 32706863

[69] Siegmund K.D. (2011). Statistical approaches for the analysis of DNA methylation microarray data. Hum Genet.

[70] Bao-Caamano A., Costa-Fraga N., Cayrefourcq L., Jácome M.A., Rodriguez-Casanova A., Muinelo-Romay L. (2023). Epigenomic analysis reveals a unique DNA methylation program of metastasis-competent circulating tumor cells in colorectal cancer. Sci Rep.

[71] Wilhelm-Benartzi C.S., Koestler D.C., Karagas M.R., Flanagan J.M., Christensen B.C., Kelsey K.T. (2013). Review of processing and analysis methods for DNA methylation array data. Br J Cancer.

[72] Kok-Sin T., Mokhtar N.M., Hassan N.Z.A., Sagap I., Rose I.M., Harun R. (2015). Identification of diagnostic markers in colorectal cancer via integrative epigenomics and genomics data. Oncol Rep.

[73] Lalchungnunga H., Hao W., Maris J.M., Asgharzadeh S., Henrich K.O., Westermann F. (2022). Genome wide DNA methylation analysis identifies novel molecular subgroups and predicts survival in neuroblastoma. Br J Cancer.

[74] Li W., Cerise J.E., Yang Y., Han H. (2017). Application of t-SNE to human genetic data. J Bioinform Comput Biol.

[75] Zhuang J., Widschwendter M., Teschendorff A.E. (2012). A comparison of feature selection and classification methods in DNA methylation studies using the Illumina Infinium platform. BMC Bioinforma.

[76] Takasawa K., Asada K., Kaneko S., Shiraishi K., Machino H., Takahashi S. (2024). Advances in cancer DNA methylation analysis with methPLIER: use of non-negative matrix factorization and knowledge-based constraints to enhance biological interpretability. Exp Mol Med.

[77] Amor R. del, Colomer A., Monteagudo C., Naranjo V. (2022). A deep embedded refined clustering approach for breast cancer distinction based on DNA methylation. Neural Comput Appl.

[78] Dallol A., Al-Maghrabi J., Buhmeida A., Gari M.A., Chaudhary A.G., Schulten H.J. (2012). Methylation of the polycomb group target genes is a possible biomarker for favorable prognosis in colorectal cancer. Cancer Epidemiol Biomark Prev.

[79] Marjoram P., Chang J., Laird P.W., Siegmund K.D. (2006). Cluster analysis for DNA methylation profiles having a detection threshold. BMC Bioinforma.

[80] Xu D., Tian Y. (2015). A comprehensive survey of clustering algorithms. Ann Data Sci.

[81] Hosseini M., Lotfi-Shahreza M., Nikpour P. (2023). Integrative analysis of DNA methylation and gene expression through machine learning identifies stomach cancer diagnostic and prognostic biomarkers. J Cell Mol Med.

[82] Houseman E.A., Christensen B.C., Yeh R.F., Marsit C.J., Karagas M.R., Wrensch M. (2008). Model-based clustering of DNA methylation array data: a recursive-partitioning algorithm for high-dimensional data arising as a mixture of beta distributions. BMC Bioinforma.

[83] Azizgolshani N., Petersen C.L., Chen Y., Levy J.J., Salas L.A., Perreard L. (2021). DNA 5-hydroxymethylcytosine in pediatric central nervous system tumors may impact tumor classification and is a positive prognostic marker. Clin Epigenetics.

[84] Koestler D.C., Christensen B.C., Marsit C.J., Kelsey K.T., Houseman E.A. (2013). Recursively partitioned mixture model clustering of DNA methylation data using biologically informed correlation structures. Stat Appl Genet Mol Biol.

[85] Wang J., Li J., Chen R., Yue H., Li W., Wu B. (2021). DNA methylation-based profiling reveals distinct clusters with survival heterogeneity in high-grade serous ovarian cancer. Clin Epigenetics.

[86] Yin X., Kong L., Liu P. (2021). Identification of prognosis-related molecular subgroups based on DNA methylation in pancreatic cancer. Clin Epigenetics.

[87] Zhong X., Zhong G. (2021). Prognostic biomarker identification and tumor classification in breast cancer patients by methylation and transcriptome analysis. FEBS Open Bio.

[88] Monti S., Tamayo P., Mesirov J., Golub T., Sebastiani P., Kohane I.S., et al. Consensus Clustering: A Resampling-Based Method for Class Discovery and Visualization of Gene Expression Microarray Data. vol. 52. 2003.

[89] Di Lena P., Nardini C., Pellegrini M. (2022). Editorial: computational methods for analysis of DNA methylation data. Front Bioinforma.

[90] Yang J., Xu J., Gao Q., Wu F., Han W., Yu C. (2022). Identification of adenylate cyclase 2 methylation in bladder cancer with implications for prognosis and immunosuppressive microenvironment. Front Oncol.

[91] Zhang M., Lv X., Jiang Y., Li G., Qiao Q. (2019). Identification of aberrantly methylated differentially expressed genes in glioblastoma multiforme and their association with patient survival. Exp Ther Med.

[92] Cheng J., Hou Y., Wang C., Guo L. (2022). Bioinformatics identification of aberrantly methylated differentially expressed genes associated with arteriosclerosis by integrative analysis of gene expression and DNA methylation datasets. Genes.

[93] Pengcheng Wang, Bingbing Wang, Zheng Zhang, Zuomin Wang Identification of inflammation-related DNA methylation biomarkers in periodontitis patients based on weighted co-expression analysis n.d. 10.18632/aging.203378 PMC8386560 34347624

[94] Feng J., Pang J., He D., Wu Z., Li Q., Ji P., et al. Identification of Genes with Altered Methylation and Its Role in Early Diagnosis of Sepsis-Induced Acute Respiratory Distress Syndrome 2021. 10.2147/IJGM.S287960. PMC7847772 33536775

[95] Li R., Shui L., Jia J., Wu C. (2020). Construction and validation of novel diagnostic and prognostic DNA methylation signatures for hepatocellular carcinoma. Front Genet.

[96] Raman P., Maddipati R., Lim K.H., Tozeren A. (2018). Pancreatic cancer survival analysis defines a signature that predicts outcome. PLoS One.

[97] Liang Y., Zhang C., Dai D.Q. (2019). Identification of differentially expressed genes regulated by methylation in colon cancer based on bioinformatics analysis. World J Gastroenterol.

[98] Ma X., Liu J., Wang H., Jiang Y., Wan Y., Xia Y. (2020). Identification of crucial aberrantly methylated and differentially expressed genes related to cervical cancer using an integrated bioinformatics analysis. Biosci Rep.

[99] Xia W.T., Qiu W.R., Yu W.K., Xu Z.C., Zhang S.H. (2023). Identifying TME signatures for cervical cancer prognosis based on GEO and TCGA databases. Heliyon.

[100] Chen F., Wang N., He X. (2022). Identification of differential genes of DNA methylation associated with Alzheimer’s disease based on integrated bioinformatics and its diagnostic significance. Front Aging Neurosci.

[101] Ritchie M.E., Phipson B., Wu D., Hu Y., Law C.W., Shi W. (2015). Limma powers differential expression analyses for RNA-sequencing and microarray studies. Nucleic Acids Res.

[102] Mohammadnejad A., Soerensen M., Baumbach J., Mengel-From J., Li W., Lund J. (2021). Novel DNA methylation marker discovery by assumption-free genome-wide association analysis of cognitive function in twins. Aging Cell.

[103] Mallik S., Odom G.J., Gao Z., Gomez L., Chen X., Wang L. (2019). An evaluation of supervised methods for identifying differentially methylated regions in Illumina methylation arrays. Brief Bioinform.

[104] Zhang W., Young J.I., Macdonald J.T., Gomez L., Schmidt M.A., Lukacsovich D., et al. Distinct CSF biomarker-associated DNA methylation in Alzheimer’s disease and cognitively normal subjects 2023. 10.21203/rs.3.rs-2391364/v1. PMC10088180 37038196

[105] Xu Y., Wang N., Liu R., Lv H., Li Z., Zhang F. (2021). Epigenetic study of esophageal carcinoma based on methylation, gene integration and weighted correlation network analysis. Onco Targets Ther.

[106] Rodriguez-Casanova A., Costa-Fraga N., Castro-Carballeira C., González-Conde M., Abuin C., Bao-Caamano A. (2022). A genome-wide cell-free DNA methylation analysis identifies an episignature associated with metastatic luminal B breast cancer. Front Cell Dev Biol.

[107] Lininger M., Spybrook J., Cheatham C.C. (2015). Hierarchical linear model: Thinking outside the traditional repeated-measures analysis-of-variance box. J Athl Train.

[108] Lin S., Yi S., Qiu P. (2022). Comprehensive analysis of TCGA data reveals correlation between DNA methylation and alternative splicing. BMC Genom.

[109] Armstrong R.A. (2019). Should Pearson’s correlation coefficient be avoided?. Ophthalmic Physiol Opt.

[110] Mallik S., Seth S., Bhadra T., Zhao Z. (2020). A linear regression and deep learning approach for detecting reliable genetic alterations in cancer using dna methylation and gene expression data. Genes.

[111] Yeung K.S., Chung B.H.Y., Choufani S., Mok M.Y., Wong W.L., Mak C.C.Y. (2017). Genome-wide DNA methylation analysis of Chinese patients with systemic lupus erythematosus identified hypomethylation in genes related to the type i interferon pathway. PLoS One.

[112] Whitley E., Ball J. (2002). Statistics review 6: nonparametric methods. Crit Care.

[113] Tang Y., Tan Y., Palaniyappan L., Yao Y., Luo Q., Li Y. (2024). Epigenetic profile of the immune system associated with symptom severity and treatment response in schizophrenia. J Psychiatry Neurosci.

[114] Charras A., Garau J., Hofmann S.R., Carlsson E., Cereda C., Russ S. (2021). DNA methylation patterns in CD8+ T cells discern psoriasis from psoriatic arthritis and correlate with cutaneous disease activity. Front Cell Dev Biol.

[115] Luo D., Yang J., Liu J., Yong X., Wang Z. (2022). Identification of four novel hub genes as monitoring biomarkers for colorectal cancer. Hereditas.

[116] Tong Lin, Zhimei Lin, Peipei Mai, E. Zhang, Lisheng Peng Identification of prognostic biomarkers associated with the occurrence of portal vein tumor thrombus in hepatocellular carcinoma n.d. 10.18632/aging.202876 PMC8109071 33878734

[117] Li C., Long Q., Zhang D., Li J., Zhang X. (2020). Identification of a four-gene panel predicting overall survival for lung adenocarcinoma. BMC Cancer.

[118] Zhao S., Wu Y., Wei Y., Xu X., Zheng J. (2022). Identification of biomarkers associated with CD8+ T cells in coronary artery disease and their pan-cancer analysis. Front Immunol.

[119] Zhu C., Zhang S., Liu D., Wang Q., Yang N., Zheng Z. (2021). A novel gene prognostic signature based on differential DNA methylation in breast cancer. Front Genet.

[120] Bodelon C., Ambatipudi S., Dugué P.A., Johansson A., Sampson J.N., Hicks B. (2019). Blood DNA methylation and breast cancer risk: A meta-analysis of four prospective cohort studies. Breast Cancer Res.

[121] Manoochehri M., Borhani N., Gerhäuser C., Assenov Y., Schönung M., Hielscher T. (2023). DNA methylation biomarkers for noninvasive detection of triple-negative breast cancer using liquid biopsy. Int J Cancer.

[122] Johnson K.C., Koestler D.C., Fleischer T., Chen P., Jenson E.G., Marotti J.D. (2015). DNA methylation in ductal carcinoma in situ related with future development of invasive breast cancer. Clin Epigenetics.

[123] Montesino-Goicolea S., Meng L., Rani A., Huo Z., Foster T.C., Fillingim R.B. (2022). Enrichment of genomic pathways based on differential DNA methylation profiles associated with knee osteoarthritis pain. Neurobiol Pain.

[124] Song Q., Decato B., Kessler M., Fang F., Qu J., Garvin T., et al. The Smithlab DNA Methylation Data Analysis Pipeline (MethPipe). 2013.

[125] Merkel A., Heath S.C. (2018).

[126] Malonzo M.H., Lähdesmäki H. (2023). LuxHMM: DNA methylation analysis with genome segmentation via hidden Markov model. BMC Bioinforma.

[127] Balaramane D., Spill Y.G., 1@ W., Bardet A.F. MethyLasso: a segmentation approach to analyze DNA methylation patterns and identify differentially methylation regions from whole-genome datasets AUTHORS AND AFFILIATIONS n.d. https://doi.org/10.1101/2023.07.27.550791. 10.1093/nar/gkae880 PMC11602171 39420630

[128] Daenekas B., Pérez E., Boniolo F., Stefan S., Benfatto S., Sill M. (2024). Conumee 2.0: enhanced copy-number variation analysis from DNA methylation arrays for humans and mice. Bioinformatics.

[129] Liu Y. (2024). methylClass: an R package to construct DNA methylation-based classification models. Brief Bioinform.

[130] Rauschert S., Raubenheimer K., Melton P.E., Huang R.C. (2020). Machine learning and clinical epigenetics: a review of challenges for diagnosis and classification. Clin Epigenetics.

[131] Zheng C., Xu R. (2020). Predicting cancer origins with a DNA methylation-based deep neural network model. PLoS One.

[132] Gomes R., Paul N., He N., Huber A.F., Jansen R.J. (2022). Application of feature selection and deep learning for cancer prediction using DNA methylation markers. Genes.

[133] Zhang G., Xue Z., Yan C., Wang J., Luo H. (2021). A novel biomarker identification approach for gastric cancer using gene expression and DNA methylation dataset. Front Genet.

[134] Liu Y., Geng H., Duan B., Yang X., Ma A., Ding X. (2021). Identification of diagnostic CpG signatures in patients with gestational diabetes mellitus via epigenome-wide association study integrated with machine learning. Biomed Res Int.

[135] Tran Q.T., Alom M.Z., Orr B.A. (2022). Comprehensive study of semi-supervised learning for DNA methylation-based supervised classification of central nervous system tumors. BMC Bioinforma.

[136] Navia-Vázquez A., Parrado-Hernández E. (2006). Support vector machine interpretation. Neurocomputing.

[137] Nguyen D.-K., Lan C.-H., Chan C.-L. (2021). Deep ensemble learning approaches in healthcare to enhance the prediction and diagnosing performance: the workflows, deployments, and surveys on the statistical, image-based, and sequential datasets. Public Health.

[138] DNA methylation biomarker selected by an ensemble machine learning approach predicts mortality risk in an HIV veteran population n.d. 10.1080/15592294.2020.1824097 PMC8216205 33092459

[139] Xu D., Li C., Zhang Y., Zhang J. (2022). DNA methylation molecular subtypes for prognosis prediction in lung adenocarcinoma. BMC Pulm Med.

[140] Guan W., Li S., Zhang Z., Xiao H., He J., Li J. (2022). Promotor methylation status of MAPK4 is a novel epigenetic biomarker for prognosis of recurrence in patients with thymic epithelial tumors. Thorac Cancer.

[141] Wu Z.H., Tang Y., Zhou Y. (2021). DNA methylation based molecular subtypes predict prognosis in breast cancer patients. Cancer Control.

[142] Peng Y., Zhao J., Yin F., Sharen G., Wu Q., Chen Q. (2021). A methylation-driven gene panel predicts survival in patients with colon cancer. FEBS Open Bio.

[143] Wang H., Wei C., Pan P., Yuan F., Cheng J. (2021). Identification of a methylomics-associated nomogram for predicting overall survival of stage I–II lung adenocarcinoma. Sci Rep.

[144] Spooner A., Chen E., Sowmya A., Sachdev P., Kochan N.A., Trollor J. (2020). A comparison of machine learning methods for survival analysis of high-dimensional clinical data for dementia prediction. Sci Rep.

[145] Rinewalt D., Shersher D.D., Daly S., Fhied C., Basu S., Mahon B. (2012). Development of a serum biomarker panel predicting recurrence in stage i non-small cell lung cancer patients. J Thorac Cardiovasc Surg.

[146] Ruiz-Perez D., Guan H., Madhivanan P., Mathee K., Narasimhan G. (2020). So you think you can PLS-DA?. BMC Bioinforma.

[147] Agarwal P., Wicklow B.A., Dart A.B., Hizon N.A., Sellers E.A.C., McGavock J.M. (2022). Integrative analysis reveals novel associations between DNA methylation and the serum metabolome of adolescents with type 2 diabetes: a cross-sectional study. Front Endocrinol (Lausanne).

[148] Marie-Claire C., Lejeune F.X., Mundwiller E., Ulveling D., Moszer I., Bellivier F. (2020). A DNA methylation signature discriminates between excellent and non-response to lithium in patients with bipolar disorder type 1. Sci Rep.

[149] Alshamlan H., Omar S., Aljurayyad R., Alabduljabbar R. (2023). Identifying effective feature selection methods for Alzheimer’s disease biomarker gene detection using machine learning. Diagnostics.

[150] Hira Z.M., Gillies D.F. (2015). A review of feature selection and feature extraction methods applied on microarray data. Adv Bioinforma.

[151] Sharif Rahmani E., Lawarde A., Lingasamy P., Moreno S.V., Salumets A., Modhukur V. (2023). MBMethPred: a computational framework for the accurate classification of childhood medulloblastoma subgroups using data integration and AI-based approaches. Front Genet.

[152] Wu J., Xiao Y., Xia C., Yang F., Li H., Shao Z. (2017). Identification of biomarkers for predicting lymph node metastasis of stomach cancer using clinical DNA methylation data. Dis Markers.

[153] Adeoye J., Wan C.C.J., Zheng L.W., Thomson P., Choi S.W., Su Y.X. (2022). Machine learning-based genome-wide salivary DNA methylation analysis for identification of noninvasive biomarkers in oral cancer diagnosis. Cancers.

[154] Yassi M., Chatterjee A., Parry M. (2023). Application of deep learning in cancer epigenetics through DNA methylation analysis. Brief Bioinform.

[155] Tu J., Chen S., Wu S., Wu T., Fan R., Kuang Z. (2022). Tumor DNA methylation profiles enable diagnosis, prognosis prediction, and screening for cervical cancer. Int J Gen Med.

[156] Nguyen T.M., Le H.L., Hwang K.B., Hong Y.C., Kim J.H. (2022). Predicting high blood pressure using DNA methylome-based machine learning models. Biomedicines.

[157] Wojtas M.A., Chen K. Feature Importance Ranking for Deep Learning. n.d.

[158] Jian F., Huang F.M., Zhang Y.H., Huang T., Cai Y.D. (2022). Identifying anal and cervical tumorigenesis-associated methylation signaling with machine learning methods. Front Oncol.

[159] Ren J., Zhou X., Guo W., Feng K., Huang T., Cai Y.D. (2022). Identification of Methylation Signatures and Rules for Sarcoma Subtypes by Machine Learning Methods. Genet Res (Camb.

[160] Song J., Huang F.M., Chen L., Feng K.Y., Jian F., Huang T. (2022). Identification of methylation signatures associated with CAR T cell in B-cell acute lymphoblastic leukemia and non-hodgkin’s lymphoma. Front Oncol.

[161] Yuan F., Ren J.X., Liao H.P., Guo W., Chen L., Feng K.Y. (2023). Identification of whole-blood DNA methylation signatures and rules associated with COVID-19 severity. Front Biosci - Landmark.

[162] Kursa M.B., Rudnicki W.R. (2010). Feature selection with the Boruta package.

[163] Guo S., Yan F., Xu J., Bao Y., Zhu J., Wang X. (2015). Identification and validation of the methylation biomarkers of non-small cell lung cancer (nsclc). Clin Epigenetics.

[164] Zeng W.J., Yang Y.L., Wen Z.P., Chen P., Chen X.P., Gong Z.C. (2020). Identification of gene expression and DNA methylation of SERPINA5 and TIMP1 as novel prognostic markers in lower-grade gliomas. PeerJ.

[165] Chin C.H., Chen S.H., Wu H.H., Ho C.W., Ko M.T., Lin C.Y. (2014). cytoHubba: Identifying hub objects and sub-networks from complex interactome. BMC Syst Biol.

[166] Ren X., Kuan P.F. (2019). methylGSA: a bioconductor package and Shiny app for DNA methylation data length bias adjustment in gene set testing. Bioinformatics.

[167] Phipson B., Maksimovic J., Oshlack A. (2016). MissMethyl: An R package for analyzing data from Illumina’s HumanMethylation450 platform. Bioinformatics.

[168] Phipson B., Maintainer J.M. (2024). Package “missMethyl”. Type Package Title Anal Illumina Hum BeadChip Data.

[169] Subramanian A., Tamayo P., Mootha V.K., Mukherjee S., Ebert B.L., Gillette M.A., et al. Gene set enrichment analysis: A knowledge-based approach for interpreting genome-wide expression profiles. 2005. 10.1073/pnas.0506580102 PMC1239896 16199517

[170] Dong D., Tian Y., Zheng S.C., Teschendorff A.E. (2019). EbGSEA: an improved gene set enrichment analysis method for epigenome-wide-association studies. Bioinformatics.

[171] Cai M., Hao Nguyen C., Mamitsuka H., Li L. (2021). XGSEA: CROSS-species gene set enrichment analysis via domain adaptation. Brief Bioinform.

[172] GSEA software n.d.

[173] Qu Z., Lau C.W., Nguyen Q.V., Zhou Y., Catchpoole D.R. (2019). Visual analytics of genomic and cancer data: a systematic review. Cancer Inf.

[174] Uehiro N., Sato F., Pu F., Tanaka S., Kawashima M., Kawaguchi K. (2016). Circulating cell-free DNA-based epigenetic assay can detect early breast cancer. Breast Cancer Res.

[175] Triche T.J., Weisenberger D.J., Van Den Berg D., Laird P.W., Siegmund K.D. (2013). Low-level processing of Illumina Infinium DNA Methylation BeadArrays. Nucleic Acids Res.

[176] Lin N., Liu J., Castle J., Wan J., Shendre A., Liu Y. (2021). Genome-wide DNA methylation profiling in human breast tissue by Illumina TruSeq methyl capture EPIC sequencing and infinium methylationEPIC beadchip microarray. Epigenetics.

[177] Ollikainen M., Ismail K., Gervin K., Kyllönen A., Hakkarainen A., Lundbom J. (2015). Genome-wide blood DNA methylation alterations at regulatory elements and heterochromatic regions in monozygotic twins discordant for obesity and liver fat. Clin Epigenetics.

[178] Bjerre M.T., Strand S.H., Nørgaard M., Kristensen H., Rasmussen A.K., Mortensen M.M. (2019). Aberrant DOCK2, GRASP, HIF3A and PKFP hypermethylation has potential as a prognostic biomarker for prostate cancer. Int J Mol Sci.

[179] Wu H.C., Delgado-Cruzata L., Flom J.D., Kappil M., Ferris J.S., Liao Y. (2011). Global methylation profles in DNA from different blood cell types.. Epigenetics.

[180] Stochastic epigenetic mutations (DNA methylation) increase exponentially in human aging and correlate with X chromosome inactivation skewing in females n.d. 10.18632/aging.100792 PMC4586102 26342808

[181] Zhang Y., Li F., Feng X., Yang H., Zhu A., Pang J. (2017). Genome-wide analysis of DNA Methylation profiles on sheep ovaries associated with prolificacy using whole-genome Bisulfite sequencing. BMC Genom.

[182] Anastasiadi D., Esteve-Codina A., Piferrer F. (2018). Consistent inverse correlation between DNA methylation of the first intron and gene expression across tissues and species. Epigenetics Chromatin.

[183] Yang R., Zheng Z., Chen Q., Yang L., Huang H., Miki D. (2017). The developmental regulator PKL is required to maintain correct DNA methylation patterns at RNA-directed DNA methylation loci. Genome Biol.

[184] Lee B.T., Barber G.P., Benet-Pagès A., Casper J., Clawson H., DIekhans M. (2022). The UCSC genome browser database: 2022 update. Nucleic Acids Res.

[185] Birney E., Andrews T.D., Bevan P., Caccamo M., Chen Y., Clarke L. (2004). An overview of ensembl. Genome Res.

[186] Li Y., Ge D., Lu C. (2019). The SMART App: an interactive web application for comprehensive DNA methylation analysis and visualization. Epigenetics Chromatin.

[187] Díez-Villanueva A., Mallona I., Peinado M.A. (2015). Wanderer, an interactive viewer to explore DNA methylation and gene expression data in human cancer. Epigenetics Chromatin.

[188] Modhukur V., Iljasenko T., Metsalu T., Lokk K., Laisk-Podar T., Vilo J. (2018). MethSurv: A web tool to perform multivariable survival analysis using DNA methylation data. Epigenomics.

[189] Sala C., Di Lena P., Durso D.F., Prodi A., Castellani G., Nardini C. (2020). Evaluation of pre-processing on the meta-analysis of DNA methylation data from the Illumina HumanMethylation450 BeadChip platform. PLoS One.

[190] Liem Y., Judge A., Kirwan J., Ourradi K., Li Y., Sharif M. (2020). Multivariable logistic and linear regression models for identification of clinically useful biomarkers for osteoarthritis. Sci Rep.

[191] Abd Elhafeez S., D’Arrigo G., Leonardis D., Fusaro M., Tripepi G., Roumeliotis S. (2021). Methods to Analyze Time-to-Event Data: The Cox Regression Analysis. Oxid Med Cell Longev.

[192] Ghaffar Nia N., Kaplanoglu E., Nasab A. (2023). Evaluation of artificial intelligence techniques in disease diagnosis and prediction. Discov Artif Intell.

[193] Shi B., Li C., Xia W., Chen Y., Chen H., Xu L. (2023). Construction a new nomogram prognostic model for predicting overall survival after radical resection of esophageal squamous cancer. Front Oncol.

[194] Ahsan M.M., Luna S.A., Siddique Z. (2022). Machine-learning-based disease diagnosis: a comprehensive review. Healthc (Switz).

[195] Onan A. (2019). Consensus clustering-based undersampling approach to imbalanced learning. Sci Program.

[196] de Souto M.C.P., Costa I.G., de Araujo D.S.A., Ludermir T.B., Schliep A. (2008). Clustering cancer gene expression data: a comparative study. BMC Bioinforma.

[197] Ozcan I., Aydin H., Cetinkaya A. (2022). Comparison of classification success rates of different machine learning algorithms in the diagnosis of breast cancer. Asian Pac J Cancer Prev.

[198] Jafari A. (2024). Machine-learning methods in detecting breast cancer and related therapeutic issues: a review. Comput Methods Biomech Biomed Eng Imaging Vis.

[199] Hameed B.S., Krishnan U.M. (2022). Artificial intelligence-driven diagnosis of pancreatic cancer. Cancers.

[200] Islam M.M., Poly T.N., Walther B.A., Yeh C.Y., Seyed-Abdul S., Li Y.C. (2022). Deep learning for the diagnosis of esophageal cancer in endoscopic images: a systematic review and meta-analysis. Cancers.

[201] Xue P., Wang J., Qin D., Yan H., Qu Y., Seery S. (2022). Deep learning in image-based breast and cervical cancer detection: a systematic review and meta-analysis. NPJ Digit Med.

[202] Forte G.C., Altmayer S., Silva R.F., Stefani M.T., Libermann L.L., Cavion C.C. (2022). Deep learning algorithms for diagnosis of lung cancer: a systematic review and meta-analysis. Cancers.

[203] Abdelwahab M.M., Al-Karawi K.A., Semary H.E. (2023). Deep learning-based prediction of Alzheimer’s disease using microarray gene expression data. Biomedicines.

[204] Torshizi A.D., Petzold L.R. (2018). Graph-based semi-supervised learning with genomic data integration using condition-responsive genes applied to phenotype classification. J Am Med Inform Assoc.

[205] Komaki S., Ohmomo H., Hachiya T., Sutoh Y., Ono K., Furukawa R. (2021). Longitudinal DNA methylation dynamics as a practical indicator in clinical epigenetics. Clin Epigenetics.

[206] Yue X., Xie Z., Li M., Wang K., Li X., Zhang X. (2022). Simultaneous profiling of histone modifications and DNA methylation via nanopore sequencing. Nat Commun.

[207] Wang Y., Zhao Y., Bollas A., Wang Y., Au K.F. (2021). Nanopore sequencing technology, bioinformatics and applications. Nat Biotechnol.

[208] Sgro A., Blancafort P. (2020). Epigenome engineering: New technologies for precision medicine. Nucleic Acids Res.

[209] Pan G., Jiang L., Tang J., Guo F. (2018). A novel computational method for detecting DNA methylation sites with DNA sequence information and physicochemical properties. Int J Mol Sci.

[210] Van Dongen J., Nivard M.G., Willemsen G., Hottenga J.J., Helmer Q., Dolan C.V. (2016). Genetic and environmental influences interact with age and sex in shaping the human methylome. Nat Commun.

[211] Mazan-Mamczarz K., Ha J., De S., Sen P. (2022). Single-cell analysis of the transcriptome and epigenome. Methods Mol Biol, Vol 2399, Hum Press Inc.

[212] Biorender n.d. https://www.biorender.com/ (Accessed December 14, 2023).

