# Supplementary material for: Methods in DNA methylation array dataset analysis: A review
Source: Comput Struct Biotechnol J. 2024 May 17;23:2304–25. doi: 10.1016/j.csbj.2024.05.015 (PMC11153885; doi:10.1016/j.csbj.2024.05.015)
Supplement: Supplementary file 1 — Supplementary material [file mmc1.docx]

| S.no. | Name | URL | Other available data types. | Description | Input for datasets search in repositories | | The output format of datasets | Reference |
| --- | --- | --- | --- | --- | --- | --- | --- | --- |
| 1. | GEO | http://www.ncbi.nlm.nih.gov/geo/info/citations.html | High throughput gene expression array.  Functional genomics datasets | Database supported by NCBI. Repository of raw and processed data of the biological experiments. | GEO accession number, Disease name, and type | RAW tar, .IDAT files | | [1] |
| 2. | ENCODE | https://www.encodeproject.org/ | Developed by the Encyclopedia of DNA Elements (ENCODE) Data Coordinating Center which acts as the primary source of ENCODE data. | The integrative-level annotation (cis-regularity elements) and ground-level annotations (experimental work) are the levels of organized data present in the portal. | Disease name, Experiment, Screening of DNA Methylation array | Metadata. tsv containing file accession, file format (FASTQ /BAM/ bigWig), Biosample term details, and accession number. | | [2] |
| 3. | EWASdb | http://www.bioapp.org/ewasdb/ | EWAS for a single marker, KEGG pathway, and GO category. | Stores the epigenetic association being the results of DNA methylation from an epigenome-wide association study. | Disease name, EWAS ID gene list, CpG site, and chromosome number | EWAS-ID, GSE-ID, Title, sample size, a summary of the experiment, and the PubMed ID | | [3] |
| 4. | ROADMAP Genomics | https://egg2.wustl.edu/roadmap/web_portal/ | The NIH Roadmap Epigenomics Mapping Consortium offers maps of histone modifications, chromatin accessibility, DNA methylation, and mRNA expression. | It is a public resource of human epigenomic data and develops tools and methods for analysis, manipulation, and estimating the epigenomic contribution towards different diseases. | Gene symbol and disease of interest. | The wig, bed, and cel files format. | | [4] |
| 5. | IHEC data portal | https://epigenomesportal.ca/ihec/index.html?as=1 | The portal provides epigenomic datasets, which have been contributed by seven international consortia: ENCODE, NIH Roadmap, CEEHRC, Blueprint, DEEP, AMED-CREST, and KNIH. | The International Human Epigenome Consortium (IHEC) coordinates the production of reference epigenome maps through the characterization of the regulome, methylome, and transcriptome. This portal also facilitates the visualization and analysis of the data. | Assembly (hg38), filter: disease = “disease of interest”. | Epigenome reference maps in the form of tab-separated text files, binary files, XML, and JSON file formats.  Selected samples can be downloaded in JSON and CSV format. | | [5] |
| 6. | iMETHYL | http://imethyl.iwate-megabank.org/ | Multiomics database containing the whole-DNA methylation and whole transcriptomics for specific cell types such as T-lymphocytes, monocytes, neutrophils, and CD4+ cells. | iMETHYL is an integrative database containing the methylome, transcriptome, and genome featuring individual DNA methylation variations. | Enter the gene number and genomic region of interest. | Details of CpG track FPKM track and SNV track. | | [6] |
| 7. | Ensembl | http://www.ensembl.org/ | Human genome annotation | It is a comprehensive source of automatic annotation of the human genome sequence, showing confirmed prediction of genes. This is also associated with the sequence analysis of data storage and visualization. | The input of the disease of interest and the filtration under the option of the restricted category (gene, transcript, variants, phenotype, somatic mutation, and protein domain) | The exported data is in a table format, or FASTA sequence. | | [7] |
| 8. | EWAS Atlas | https://bigd.big.ac.cn/ewas | Epigenomics | Defines a curated knowledge base of Epigenome-Wide Association Study (EWAS) majorly targeting DNA methylation as the epigenetic mark. | The input of the “disease of interest” in the BROWSE tab of the portal. | The data can be exported in the CSV and TXT format. | | [8] |
| 9. | BLUEPRINT Epigenome | http://blueprint-data.bsc.es | RNA-seq, ChIP-Seq, DNase1-Seq, WGS Bisulphite Seq | Reference epigenomes of normal and malignant  hematopoietic cells from humans with blood-based diseases such as common leukemia and autoimmune disease | The input search can be gene symbol, Ensembl gene ID, chromosome region, or Reactome pathway. | - | | [9] |
| 10. | The German Epigenome Programme (DEEP) | http://deep.dkfz.de | Bisulphite-seq, total RNA-seq, mRNA-seq, ChIP-seq | Reference epigenomes of human cells and tissues in  normal and complex disease states especially concerning metabolic diseases such as steatosis and adiposities’ as well as inflammatory diseases of the joints and the intestine. | Filters with cell lines, tissue, gender, and experiment type are available | BigBed/BigWig files | | [10] |
| 11. | Allelic Epigenome Project | http://genboree.org/genboreeKB/projects/allelic-epigenome | Nil | Allelic DNA methylome, histone modifications, and  transcriptome in human cells and tissues | Data can be downloaded with the Genboree KBs plugin, | JSON/TSV format | | [11] |
| 12. | Fetal brain meQTLs | https://epigenetics.essex.ac.uk/mQTL/ | Characterization of DNA methylation quantitative trait loci | Epigenome-wide significant meQTLs observed in fetal  Brain. | Input can be probe ID, rsID, and chromosome position. | CSV/txt/xslx | | [12] |
| 13. | WashU Epigenome Browser | https://epigenomegateway.wustl.edu/browser/ | Only epigenomic datasets | A web browser showing collaborative results from ENCODE and Roadmap  Epigenomics projects | Input can be gene symbol, SNP ID, chr. region search. | - | | [13] |
| 14. | dbGaP | http://www.ncbi.nlm.nih.gov/gap | Genome-wide association studies (GWAS), phenotypes, and molecular diagnostics studies. | Database supported by National Institutes of Health-sponsored repository.  This is charged to organize, preserve, and distribute information produced by studies investigating the interaction between genotype and phenotype. | Study Identifier (Accession Number).  Study types such as GWAS, TWAS, etc. | Open and controlled phenotype datasets files, study documents, variable reports, and data dictionaries. | | [14] |
| The global repositories targeting cancer as a disease of interest | | | | | | | | |
| 15. | MethCNA | <http://cgma.scu.edu.cn/MethCNA>. | Integrates the genomic and epigenomics data of human cancer | This database can help researchers explore DNA methylation and copy number alteration patterns for identifying the oncogenes and the development of targeted therapies. | Input the sample ID specific to the disease of interest (GEO- sample ID) | The visualization of DNA methylation & copy number alterations plots is visible. | | [15] |
| 16. | cBioportal | <http://cbioportal.org> | DNA, RNA, Protein. Also complements TCGA and ICGC data portals the UCSC Cancer Genomics Browser and IntOGen. | cBio Cancer Genomics Portal (cBioportal) is an open-access resource for the interactive exploration of multidimensional cancer genomics data sets. | Disease name and type | Tab-separated text files, MAF files, and JSON files, containing each row representing a sample and each column representing a genomic feature or clinical attributes such as gene expression values, and mutation status. | | [16] |
| 17. | DNMIVD | http://www.unimd.org/dnmivd/ | DNMIVD collected the RNA-Seq, methylation, and clinical data from TCGA as well as meQTL, e- mQTL and pathway- meQTL information. | This database provides enriched information from the TCGA cancer subtypes and visualizes different categories of DNA methylation resources, including models to screen diagnostic and prognostic markers, DVMC, emQTL, pathway-meQTL, FEM and survival analysis. | The download section gives the list of links containing datasets derived from TCGA (expression-methylation, pathway, and cancer-associated pathway-meQTL. | The selection of the disease of interest gives a txt.gz downloadable format. | | [17] |
| 18. | TCGA | https://portal.gdc.cancer.gov/ | RNA seq data, miRNAseq, DNAseq, SNP-based platform, Array-based DNA methylation sequencing data, and Reverse-phase protein array (RPPA) | TCGA involves several cooperating centers responsible for the collection and sample processing, followed by high-throughput sequencing and sophisticated bioinformatics data analysis. | A filter of data categories such as DNA methylation data, data type; experiment strategy, and platform. | The data format type is. TXT format | | [18] |
| 19. | ICGC | https://dcc.icgc.org/ | Genomics, Transcriptomics | This project aims to discover available genetic faults in large numbers of individual cancers and the cancer sample studied is freely available on platforms. | A filter of a repository, data type, and experiment strategy (as Bisulphide-seq). | FASTQ format. | | [18] |
| 20. | COSMIC | https://cancer.sanger.ac.uk | Genomics, epigenomics, transcriptomics | This database is expert-curated and overlapped with other portals. Includes the details for coding mutation, the genetic mechanism by somatic mutation promoting cancer, non-coding mutation copy number variants, and drug-resistance mutations. | The keyword entered is the disease of interest. | BAM files and .cel files | | [19] |
| 21. | Pancan-meQTL | http://gong_lab.hzau.edu.cn/Pancan-meQTL/ | cis-meQTLs and trans-meQTLs across different cancer types. | Database of cis- and trans- meQTLs across 23 cancer  types from The Cancer Genome Atlas. | SNP or methylated gene symbol | CSV format | | [20] |

**Supplementary Table I:** The list of global repositories storing the comprehensive DNA methylation array data for different diseases.

a. ENCODE: Encyclopedia of DNA Elements, b. GEO: Gene Expression Omnibus, c. EWAS: epigenome-wide association database, d. cBioportal: epigenome-wide association database, e. IHEC: International Human Epigenome Consortium, f. DNMIVD: DNA methylation interactive visualization database, g. TCGA: The cancer genome atlas. h. ICGC: International Cancer Genome Consortium, i. COSMIC: The Catalogue of Somatic Mutations in Cancer; j. CEEHRC: Canadian Epigenetics, Environment and Health Research Consortium Network.
