# Supplementary material for: Methods in DNA methylation array dataset analysis: A review
Source: Comput Struct Biotechnol J. 2024 May 17;23:2304–25. doi: 10.1016/j.csbj.2024.05.015 (PMC11153885; doi:10.1016/j.csbj.2024.05.015)
Supplement: Supplementary file 2 — Supplementary material [file mmc2.docx]

| S.no | Title | Datasets selection method | Source | Sample size | Representativeness | References |
| --- | --- | --- | --- | --- | --- | --- |
| 1. | DNA methylation array analyses identified breast cancer-associated HYAL2 methylation in peripheral blood | The epigenome-wide screening was done using Human Methylation27 BeadChip (Illumina). | Peripheral blood samples | 72 familial BC cases and 24 controls | Peripheral blood samples from BC cases and healthy controls were obtained from centers in Southwest Germany. All the individuals were Caucasian. | PMID: **25213452** |
| 2. | Association of variably methylated tumor DNA regions with overall survival for invasive lobular breast cancer | Melbourne Collaborative Cohort Study (MCCS):  The tumor samples of ILBC (invasive lobular breast cancer) were mainly ER-positive, PR-positive, and HER2-negative.  TCGA database:  Tumour DNA methylation was measured using the HumanMethylation 450K (HM450K) BeadChip array. | Tumor-enriched DNA was prepared from 130 ILBCs in the Melbourne Collaborative Cohort Study participants, using FFPE tissue and genome-wide DNA methylation data from the TCGA database. | 130 ILBCs were diagnosed in the participants of the Melbourne Collaborative Cohort Study (MCCS).  The data was retrieved from The Cancer Genome Atlas (TCGA) for 168 ILBC cases. | Melbourne Collaborative Cohort Study was a study conducted in Australia.  Samples were estimated to have been diagnosed with invasive breast cancer in the United States. | PMID: 33461604 |
| 3. | Association of DNA-Methylation Profiles With Immune Responses Elicited in Breast Cancer Patients Immunized With a Carbohydrate-Mimicking Peptide: | Genomic DNA from patients was bisulfite-treated and purified using the EZ DNA Methylation-Gold kit, and genome-wide DNA methylation was assessed using the Infinium Methylation EPIC BeadChip array. | Isolation of DNA from Peripheral Blood Mononuclear Cells (PBMCs) | - | Patients were recruited from the WPRCI Breast Cancer Clinic at the UAMS campus and the Highlands Oncology Group (HOG) Oncology Clinic in Northwest Arkansas. | PMID: 32582547 |
| 4. | DNA methylation markers predict recurrence-free interval in triple-negative breast cancer | Bisulfite conversion was performed on FFPE tumor tissue sections or one tumor-targeted core of IBCSG samples, and genomic DNA was analyzed using the Illumina Infinium HumanMethylation450K BeadChip Kit. | Tumor samples given with locoregional treatment were collected from institutional cohorts and subjected to methylation array analysis. | 110 triple-negative breast cancer tumor samples. | The International Breast Cancer Study Group (IBCSG), established in 1978, comprises member institutions from various countries, including Switzerland, Australia, New Zealand, Sweden, Italy, Slovenia, South Africa, Spain, Canada, Hong Kong, and others. | PMID: 32025567 |
| 5. | DNA methylation signatures of breast cancer in peripheral T-cells | DNA extracted from isolated T cells was subjected to Illumina 450 K DNA methylation array analysis. | Isolation of T cells from peripheral blood samples of patients | Nine ladies with stages 3 and 4, nine age-matched healthy women, and 19 females with stages 1 and 2 were enrolled. | Peripheral blood samples from healthy controls and breast cancer patients were obtained from the oncology clinic of MUHC | PMID: 29776342 |
| 6. | Breast cancer subtype dictates DNA methylation and ALDH1A3-mediated expression of tumor suppressor RARRES1 | Methylation analyses using the HM450 array (Illumina).  cBioportal was used to analyze TCGA data or the data was taken directly from the TCGA Data Portal. | Isolation of DNA from untreated and DAC-treated cells | RARRES1 expression was decoded for each of the 26 breast cell lines and 220 breast cancer samples. | NIL | PMID: 27286452 |
| 7. | Towards understanding the breast cancer epigenome: a comparison of genome-wide DNA methylation and gene expression data | DNA methylation and gene expression data were obtained from the publicly available Gene Expression Omnibus database.  Methylation analysis was performed using Infinium Methylation 27 K arrays | Isolation of DNA from diseased and normal samples | The breast cancer data set consists of 248 samples, which is split into Series 1 (total =123, includes 119 breast and 4 normal) and Series 2 (total =125, includes 117 breast and 8 normal) | Toronto, Canada | PMID: 26657508 |
| 8. | Impact of chemotherapy for breast cancer on leukocyte DNA methylation landscape and cognitive function: a prospective study | DNA samples were subjected to bisulfite conversion and were subjected to DNA methylation microarray assay using Illumina Infinium HumanMethylation450 Beadchip. | Diseased and controlled breast cancer whole blood samples. | 93 cases of breast cancer with linked pre- and post-chemotherapy samples were included in this investigation. | All blood samples were collected at NCORP sites and shipped to CCPL at the University of Rochester Cancer Center NCORP Research Base. | PMID: 30867049 |
| 9. | DNA methylome analysis identifies accelerated epigenetic aging associated with postmenopausal breast cancer susceptibility | DNA methylation profiling embedded in the European Prospective Investigation into Cancer and Nutrition (EPIC) cohort using the Illumina HumanMethylation 450K BeadChip arrays | Case-control patients’ blood samples | This study involved 960 case-controlled samples with 480 postmenopausal breast cancer | The EPIC cohort, a large study across ten European countries, examines the link between diet, lifestyle, metabolism, and cancer risk.  This study was conducted using 23 centers across 10 European countries (Denmark, France, Germany, Greece, Italy, Norway, Spain, Sweden, The Netherlands, and the United Kingdom). |  |
| 10. | DNA methylation signatures of Prostate Cancer in peripheral T-cells | The T cells' DNA was analyzed using the Illumina Infinium methylation EPIC array. | The study compared the peripheral blood T cells of men with positive prostate cancer biopsy to those with negative biopsy. | The study had 12 controls and 20 PCa samples | The APCaRI Registry and Biorepository connects biospecimens and data from over 4 million Albertan patients. | PMID: 32576165 |
| 11 | Bioinformatic analysis and experimental validation identified DNA methylation-related biomarkers and immune-cell infiltration of atherosclerosis | The methylation levels were evaluated based on the Illumina Infinium Human Methylation 450 Beadchip platform and quantified as a β-value. | Sequencing of bisulfite-converted DNA and array-based analysis of atherosclerotic lesions and normal carotid tissue. | The study used 15 atherosclerotic and paired healthy tissues | The study was conducted in Spain | PMID: 36159969 |
| 12. | Distinct DNA Methylation Signatures in Neuroendocrine Tumors Specific for Primary Site and Inherited Predisposition | The genome-wide DNA methylation assays were performed using the Infinium MethylationEPIC BeadChip Array | The tumor portion was microdissected from NET patients. | Genome-wide DNA methylation of 96 neuroendocrine tumors (NET) samples. | Clinical trials conducted in the United States | PMID: 32706863 |
| 13. | Multifactorial analysis of the stochastic epigenetic variability in cord blood confirmed an impact of common behavioral and environmental factors but not of in vitro conception | Genomic DNA was extracted from cord blood and subjected to bisulphide conversion which was then used for hybridization in Infinium HumanMethylation 450 BeadChip. | Samples of cord blood from both ART-conceived pregnancies and naturally conceived pregnant women. | 23 ART-conceived and 41 naturally-conceived pregnancies | The samples were obtained from the midwives of the San Raffaele Hospital, Italy, at the time of delivery. | PMID: 29930742 |
| 14. | Identification of diagnostic markers in colorectal cancer via integrative epigenomics and genomics data | Methylation profiling was performed using the Illumina InfiniumHumanMethylation27 BeadChip | Case-control epithelial cells | 55 paired cancer and adjacent normal epithelial cells | Patients from the University Kebangsaan Malaysia Medical Centre in Kuala Lumpur, Malaysia, provided the samples during surgery. | PMID: 25997610 |
| 15. | Genome-wide DNA methylation analysis identifies novel molecular subgroups and predicts survival in neuroblastoma | Methylation data were derived from HumanMethylation450 BeadChip arrays | DNA methylation for primary neuroblastoma samples | The TARGET study yielded data on 223 samples' genome-wide DNA methylation, 130 samples' gene expression, 213 samples' genetic and clinical information, and 130 samples' whole-exome sequencing information. | Two pilot investigations characterized the transcriptomes and genomes of "high-risk" subtypes of neuroblastoma (NBL) and acute lymphoblastic leukemia (ALL). | PMID: 36175618 |
| 16. | A deep embedded refined clustering approach for breast cancer distinction based on DNA methylation | The Illumina Infinium 27k Human DNA methylation Beadchip v1.2 was employed at about 27,000 CpGs from women with and without breast cancer to collect the methylation data. | DNA methylation profiles obtained from breast cancer tumors | There are 23 non-neoplastic cases and 114 breast cancer cases in the first dataset.  There are 39 cases of breast cancer and 9 normal controls in the second dataset. | United Kingdom | [cite the paper using Mendeley) |
| 17. | Integrative analysis of DNA methylation and gene expression through machine learning identifies stomach cancer diagnostic and prognostic biomarkers | The Cancer Genome Atlas (TCGA) stomach adenocarcinoma (STAD) data samples were used with the use of Illumina HumanMethylation27 and 450 BeadChip platform. | Diseased-control tumor samples | dataset with 25 non-tumor and 48 STAD tumor samples (27 K array)  450 K array data containing 395 tumor and 2 non-tumor STAD samples. | TCGA-STAD samples were downloaded TCGA-GDC portal | PMID: 36779430 |
| 18. | DNA 5-hydroxymethylcytosine in pediatric central nervous system tumors may impact tumor classification and is a positive prognostic marker | DNA methylation profiling of tumor and non-tumor samples using Illumina Human Methylation EPIC Beadchips | Diseased-control tumor samples. | A total of 27 distinct people had their fresh-frozen initial CNS tumor specimens identified; they included 13 gliomas, 8 ependymomas, and 6 embryonal tumors. | All patients were treated at Dartmouth Hitchcock Medical Center. | PMID: 34538273 |
| 19. | DNA methylation-based profiling reveals distinct clusters with survival heterogeneity in high-grade serous ovarian cancer | Using DNA methylation data from The Cancer Genome Atlas database, the current study discovered four distinct HGSOC methylation subgroups. | Only tumour or diseased samples | 479 high-grade serous ovarian cancer patients’ samples are included. | Assessment of DNA methylation of HGSOC subtypes of samples obtained from TCGA. | PMID: 34645493 |
| 20. | Prognostic biomarker identification and tumor classification in breast cancer patients by methylation and transcriptome analysis | RNA-sequencing (RNA-Seq) transcriptome data and DNA methylation data of the TCGA-BRCA dataset were obtained from The Cancer Genome Atlas. | Diseased and normal tumor samples | 96 normal and 796 tumor samples were taken from the TCGA database | Assessment of DNA methylation of BRAC samples obtained from TCGA repositories. | PMID: 34056873 |
| 21. | Identification of adenylate cyclase 2 methylation in bladder cancer with implications for prognosis and immunosuppressive microenvironment | DNA methylation profiling data from the platform (Illumina HumanMethylation27 BeadChip | Diseased and controlled bladder cancer samples | In total, five normal bladder mucosae and 18 primary BCa samples were included | Data collection was done from public repositories (GEO database) belonging to South – Korea. | PMID: 36313639 |
| 22. | Identification of aberrantly methylated differentially expressed genes in glioblastoma multiforme and their association with patient survival | DNA methylation profiling data from the platform (Illumina HumanMethylation27 BeadChip | Diseased and control glioblastoma multiforme (GBM) samples | 54 GBM and 24 control brain tissue. | Data collection is done from public repositories (GEO databases) belonging to the USA. | PMID: 31452706 |
| 23. | Bioinformatics Identification of Aberrantly Methylated Differentially Expressed Genes Associated with Arteriosclerosis by Integrative Analysis of Gene Expression and DNA Methylation Datasets | DNA methylation profiling data from the platform (Illumina HumanMethylation 450 BeadChip | Diseased and controlled atherosclerotic samples | 19 carotid atherosclerotic tissue samples and 15 control aortic tissue. | Data collection was done from public repositories (GEO databases) belonging to Spain. | PMID: 36292702 |
| 24. | Identification of inflammation-related DNA methylation biomarkers in periodontitis patients based on weighted co-expression analysis | DNA methylation profiling data from the platform (Illumina HumanMethylation 450 BeadChip | periodontitis and healthy control tissue samples | 23 samples with 11 normal and 12 diseased samples | Data collection is done from public repositories (GEO databases) belonging to Canada. | PMID: 34347624 |
| 25. | Identification of Genes with Altered Methylation and Its Role in Early Diagnosis of Sepsis-Induced Acute Respiratory Distress Syndrome | DNA methylation data analysis based on platform ((Illumina HumanMethylation 450 BeadChip) | Diseased and controlled acute respiratory distress syndrome | blood samples from 39 patients with ARDS, 75 non-ARDS ICU controls, and 30 healthy controls | Data collection is done from public repositories (GEO databases) belonging to the USA. | PMID: 33536775 |
| 26 | Construction and Validation of Novel Diagnostic and Prognostic DNA Methylation Signatures for Hepatocellular Carcinoma | Whole-genome DNA methylation profiles based on the Illumina HumanMethylation450 BeadChip Assay | Diseased and control samples of Hepatocellular Carcinoma | Tissue samples from 836 HCC patients and 303 normal controls were gathered for analysis. | Assessment of DNA methylation of HCC samples obtained from TCGA repositories | PMID: 32922438 |
| 27. | Pancreatic cancer survival analysis defines a signature that predicts the outcome | Methylation data for the TCGA PAAD dataset was downloaded from the cBioPortal database | Positive survival groups and negative survival groups | 28 samples of survival+ groups and 19 samples of survival- groups. | Assessment of DNA methylation of Pancreatic cancer samples obtained from TCGA repositories |  |
| 28. | Identification of differentially expressed genes regulated by methylation in colon cancer based on bioinformatics analysis | Illumina HumanMethylation450 BeadChip Assay platform is used for DNA methylation profiling | Diseased and control samples of colon cancer | A total of 314 tumor tissues and 37 normal tissues patients’ samples | Assessment of DNA methylation of colon cancer samples obtained from TCGA repositories | PMID: 31341364 |
| 29. | Identification of crucial aberrantly methylated and differentially expressed genes related to cervical cancer using an integrated bioinformatics analysis | Illumina HumanMethylation450 BeadChip Assay platform is used for DNA methylation profiling.  Gene expression profiling based on the platform Affymetrix Human Genome U133 Plus 2.0 Array | Diseased and control samples of cervical cancer | 20 normal samples and 6 samples with cervical cancer were included in the methylation-profile microarray data.  The microarray data used for gene expression profiling included 24 normal and 33 samples of cervical cancer. | DNA methylation dataset: Sweden  Gene expression datasets: USA | PMID: 32368784 |
| 30 | Identifying TME signatures for cervical cancer prognosis based on GEO and TCGA databases | Illumina HumanMethylation27 BeadChip Assay platform is used for DNA methylation profiling.  From the TCGA portal, RNA gene expression data and related clinical data for CESC patients were gathered. | All diseased CESC patients’ samples | RNA gene expression data: 306 CESC patients were enrolled in the study.  DNA methylation:  215 CESC patients were enrolled in the study. | For CESC patients, RNA gene expression data and related clinical information were obtained via the TCGA data portal.  Similarly, DNA methylation data was obtained from the GEO data portal. | PMID: 37095983 |
| 31 | Epigenetic Study of Esophageal Carcinoma Based on Methylation, Gene Integration, and Weighted Correlation Network Analysis | Illumina HumanMethylation450 BeadChip Assay platform is used for DNA methylation profiling.  Gene expression profiling based on the platform IlluminaHiSeq_RNASeqV | The data were from primary solid tumor tissue samples of patients with Esophageal carcinoma and normal tissue samples | For the examination of DNA methylation data, 185 samples were employed. Among them, 170 common samples showed similar DNA methylation and mRNA expression (161 cases and 9 normal controls). | Assessment of DNA methylation and gene expression data of Esophageal Carcinoma samples obtained from TCGA repositories | PMID: 34012270 |
| 32. | Identification of Differential Genes of DNA Methylation Associated With Alzheimer's Disease Based on Integrated Bioinformatics and Its Diagnostic Significance | Illumina HumanMethylation450 BeadChip Assay platform is used for DNA methylation profiling.  Gene expression profiling based on the platform IlluminaHiSeq_RNASeqV | Diseased and controlled samples of Alzheimer's disease from the Middle temporal gyrus (MTG) of the brain | DNA methylation data:  Obtained from 78 samples derived from the MTG of the brain (32 normal and 46 AD patients).  Gene expression: 19 blood samples with 10 healthy and 9 from AD patients. | Methylation data: Germany  Gene expression: USA | PMID: 35615586 |
| 33. | A Linear Regression and Deep Learning Approach for Detecting Reliable Genetic Alterations in Cancer Using DNA Methylation and Gene Expression Data | Illumina HumanMethylation450 BeadChip Assay platform is used for DNA methylation profiling. | Diseased and controlled cervical cancer tumor samples | This dataset included 63 uterine cervical tumor samples and 152 matched normal samples. | United Kingdom | PMID: 32806782 |
| 34 | Novel DNA methylation marker discovery by assumption-free genome-wide association analysis of cognitive function in twins | DNA methylation profiles in whole blood samples were analyzed using the Infinium HumanMethylation450 BeadChips (Illumina) containing 485,512 CpG sites across the human genome. | The whole blood samples were collected during 2008–11 in a follow-up assessment. | 400 monozygotic (MZ) twins, ages 56 to 80, were enrolled in the middle-aged Danish twin (MADT) project through the Danish Twin Registry. Of them, 220 male and 180 female pairs (Table S1) were recruited. | The study sample was recruited by the Danish Twin Registry | PMID: 33528912 |
| 35. | A genome-wide cell-free DNA methylation analysis identifies a signature associated with metastatic luminal B breast cancer | The Infinium Human MethylationEpic BeadChips were used to assess the DNA methylation patterns of plasma cfDNA. | Blood and tissue samples were collected from metastatic LBBC patients and nontumor controls. | Plasma cfDNA was examined in a group of 14 women, which included nontumor controls and metastatic LBBC patients. | The study samples were collected in the medical oncology department of the University Clinical Hospital of Santiago de Compostela (Spain) between 2016 and 2018. | PMID: 36393855 |
| 36. | Distinct CSF biomarker-associated DNA methylation in Alzheimer's disease and cognitively normal subjects | The Infinium Human MethylationEpic BeadChips were used to assess the DNA methylation patterns of plasma cfDNA. | The blood sample dataset included 202 DNA methylation samples cognitively normal (CN) and Alzheimer's disease (AD) samples | 202 DNA methylation samples (123 CN and 79 AD). | The goal of the ADNI study is to characterize the progression of AD31. The dataset was produced with individual samples. | PMID: 36865230 |
| 37. | DNA Methylation Patterns in CD8+ T Cells Discern Psoriasis From Psoriatic Arthritis and Correlate With Cutaneous Disease Activity | The Infinium Human MethylationEpic BeadChips were used to assess the DNA methylation patterns of blood samples | Peripheral blood CD8+ T cells from healthy and diseased samples | 26 individuals with nine healthy controls, 10 psoriasis, and seven PsA patients | The study cohort contains patients with skin psoriasis and PsA with ethnicity Caucasian | PMID: 34746142 |
| 38 | A Novel Gene Prognostic Signature Based on Differential DNA Methylation in Breast Cancer | RNA-seq data in FPKM format and clinical information on BC were downloaded from the Cancer Genome Atlas database.  Data from the TCGA database were acquired from UCSC Xena for the Illumina Human Methylation450 BeadChip array (450k array) and the Illumina Human Methylation27 BeadChip array | Tumour samples of breast cancer patients | 557 samples of breast cancer patients with both methylation and gene expression | Evaluation of breast cancer samples' DNA methylation and gene expression data retrieved from TCGA repositories | PMID: 34956313 |
| 39. | Blood DNA methylation and breast cancer risk: a meta-analysis of four prospective cohort studies | The Infinium Human MethylationEpic BeadChips were used to assess the DNA methylation patterns of blood samples | Blood samples of breast cancer patients | The average age of patients varies from 52.2 and 62.2 years in the four groups of patients with breast cancer. | Four prospective studies were combined that are the MCCS EPIC-Italy, the IARC cohort of the European Prospective Investigation into Cancer and Nutrition (EPIC-IARC) | PMID: 31101124 |
| 40. | Enrichment of genomic pathways based on differential DNA methylation profiles associated with knee osteoarthritis pain | The Infinium Human MethylationEpic BeadChips were used to assess the DNA methylation patterns of blood samples | Blood samples of patients reported unilateral or bilateral knee pain and screened positive for clinical knee OA | This study evaluates DNA methylation profile associations with knee OA-related pain in a large sample of middle-aged adults | Adults living in the community who were enrolled in an observational study at the Universities of Florida (UF) and Alabama at Birmingham (UAB) were recruited for sample collection. | PMID: 36531611 |
| 41. | Application of Feature Selection and Deep Learning for Cancer Prediction Using DNA Methylation Markers | Illumina 27K and 450K datasets from the Breast Invasive Carcinoma project were used to assess the DNA methylation profiles of tumor tissues, which were accessed through the GDC Data Portal. | The tumor samples of disease types chosen were Ductal and Lobular Neoplasms. | A total of 1188 samples were retrieved. | Evaluation of the gene expression and DNA methylation of project samples for invasive breast cancer that were acquired from TCGA repositories. | PMID: 36140725 |
| 42. | Predicting cancer origins with a DNA methylation-based deep neural network model. | DNA methylation profiles of tumor samples were analyzed using Illumina 450K datasets of cancer patients. | Training and evaluation of the cancer origin classifier were conducted using TCGA data, which were stratified and randomly divided into three groups: training (n = 4,403), development (n = 1,468), and test (n = 1,468). | The final data include DNA methylation data and clinical information from 7,339 patients of 18 cancer origins. | Assessment of DNA methylation and gene expression data of Cancer samples obtained from TCGA repositories |  |
| 43. | A Novel Biomarker Identification Approach for Gastric Cancer Using Gene Expression and DNA Methylation Dataset | The gene expression data and DNA methylation data of gastric cancer are downloaded from GEO to construct our experiment dataset.  Platform for gene expression: Affymetrix Human Genome U133A Array.  Platform for DNA methylation array data:  Illumina HumanMethylation27 BeadChip | Tumour samples from gastric cancer patients | There are 268 samples of gene expression data including 134 tumor samples, 134 normal samples, and 13,515 features. DNA methylation data contains 203 tumor samples, 94 normal samples, and 14,476 features. | Assessment of DNA methylation and gene expression data of Gastric Cancer samples obtained from TCGA repositories | PMID: 33868380 |
| 44. | Identification of Biomarkers for Predicting Lymph Node Metastasis of Stomach Cancer Using Clinical DNA Methylation Data | The DNA methylation data of stomach cancer are downloaded from TCGA to construct our experiment dataset. | Lymph node metastasis and normal stomach cancer patients’ samples. | Lymph node (LN) negative, LN positive, and Unclassified groups contain 94, 189, and 12 samples.  27 normal samples | Assessment of DNA methylation of Stomach Cancer samples obtained from TCGA repositories. | PMID: 28951630 |
| 45. | Identification of Diagnostic CpG Signatures in Patients with Gestational Diabetes Mellitus via Epigenome-Wide Association Study Integrated with Machine Learning | The DNA methylation data of Gestational Diabetes Mellitus are downloaded from GEO and epigenome-wide association study (EWAS) to construct our experiment dataset.  Platform: Illumina HumanMethylation450 BeadChip assays. | umbilical cord blood samples and peripheral cord blood samples of Gestational Diabetes Mellitus patients | First dataset: there were 64 controls without GDM and 68 umbilical cord blood samples from babies whose moms had GDM.  Peripheral blood samples from 18 complete sibling pairs that experienced various forms of intrauterine hyperglycemia—pregnant with gestational diabetes mellitus or not— | Assessment of DNA methylation of Gestational Diabetes Mellitus samples obtained from GEO repository.  Country: Germany and South Korea | PMID: 34104645 |
| 46. | DNA methylation molecular subtypes for prognosis prediction in lung adenocarcinoma | RNA-Seq standardized FPKM data and clinical comparison data from TCGA-LUAD.  Methylation data from Illumina Infinium was obtained from the UCSC Cancer Browse. | Tumour samples of lung adenocarcinoma patients. | RNA-seq: 486 samples.  Methylation samples:  150 (27K) and 503 (450K) samples. | Assessment of DNA methylation and gene expression of lung adenocarcinoma samples obtained from TCGA repositories. | PMID: 35392867 |
| 47. | The promotor methylation status of MAPK4 is a novel epigenetic biomarker for the prognosis of recurrence in patients with thymic epithelial tumors | The methylation dataset analyzed with Human Methylation450 of TETs was downloaded from UCSC  A raw count matrix of gene-level RSEM values from 120 TET tumor samples was acquired from Firehose. | Tumour samples of TETs patients | The dataset included 113 cases of thymoma and 11 cases of thymic carcinoma. | Nil | PMID: 36073321 |
| 48. | DNA Methylation-Based Molecular Subtypes Predict Prognosis in Breast Cancer Patients | RNA-seq data:  Downloaded from 3 gene expression information (standardized FPKM) of the TCGA-BC cohort.  Methylation data:  Samples from the UCSC genome browser on Illumina Infinium Human Methylation 27 and 450 BeadChip arrays. | Tumour samples of breast cancer patients | RNA-seq dataset includes 1053 cases.  Methylation datasets include 342 and 890 patients. | Assessment of DNA methylation and gene expression of Breast cancer samples obtained from TCGA repositories. | PMID: 33504182 |
| 49. | A methylation‐driven gene panel predicts survival in patients with colon cancer | The TCGA data portal provided transcriptome profiles from colon adenocarcinoma (COAD) tissues and DNA methylation data obtained from the Illumina Human Methylation 450 Beadchip.  The dataset for validation was taken from GEO. | Tumour and adjacent normal samples | 353 DNA methylation samples with 315 COAD samples and 38 tumor‐adjacent samples.  521 transcriptome profiles with 41 cases from tumor‐adjacent tissues and 480 cases were COAD tissues. | Assessment of DNA methylation of colon cancer samples obtained from TCGA and GEO repositories. | PMID: 34184409 |
| 50. | Identification of a methylomics-associated nomogram for predicting overall survival of stage I–II lung adenocarcinoma | Patients' DNA methylation information from the TCGA database with stages I–II LUAD was analyzed with the Illumina HumanMethylation450 BeadChip array | Stage I and Stage II samples of lung adenocarcinoma | Data for a total of 393 stage I–II LUAD patients | Assessment of DNA methylation of stage I–II lung adenocarcinoma samples obtained from TCGA repositories. | PMID: 33976305 |
| 51. | Identification of prognosis-related molecular subgroups based on DNA methylation in pancreatic cancer | The UCSC Xena platform hosted the DNA methylation data of PC patients that were produced by the Illumina Human Methylation 450 platform. The Genomic Data Commons Data Portal of the TCGA database provided the RNA sequencing (HTSeq–FPKM type), somatic mutation (MuTect2 Annotation), and copy number variation (Masked Copy Number Segment type) data of PC patients. | Tumour and peripheral fluid samples | 178 pancreatic cancer patients’ samples | Assessment of DNA methylation pancreatic cancer samples obtained from TCGA repositories. | PMID: 33980289 |
| 52. | Epigenomic signature of major congenital heart defects in newborns with Down syndrome | Whole genome bisulfite sequencing done by  Illumina NovaSeq 6000 S4 flow cell.  DNA methylation information from Illumina Infinium Methylation EPIC (EPIC) DNA methylation arrays was obtainable from NDBS for each sample. | They focused on patient’s DNA sample suffering from major heart defects including Down syndrome and Congenital heart defects. | 45 DS-CHD (27 female, 18 male) and 2) 41 DS non-CHD (27 female, 14 male) | The Institutional Review Boards of the University of Southern California, the University of California, Davis, and the California Health and Human Services Agency authorized this study. | PMID: 37205408 |

**Supplementary Table II: The table summarizes information related to the retrieved data used in the majority of research articles, ensuring information about their source, sample size, representativeness, and selection methods.**

a. WPRCI: Winthrop P. Rockefeller Cancer Institute; IBCSG: International Breast Cancer Study Group; CCPL: Cancer Control and Psychoneuroimmunology Lab; EPIC: European Prospective Investigation into Cancer and Nutrition.
