# Supplementary material for: Methods in DNA methylation array dataset analysis: A review
Source: Comput Struct Biotechnol J. 2024 May 17;23:2304–25. doi: 10.1016/j.csbj.2024.05.015 (PMC11153885; doi:10.1016/j.csbj.2024.05.015)
Supplement: Supplementary file 3 — Supplementary material [file mmc3.docx]

| S.no | Method | Details/Explanation | Year | Workflows/packages | Drawbacks or assumptions | Comparative analysis | Citat-ion | References |
| --- | --- | --- | --- | --- | --- | --- | --- | --- |
| Within- array Normalization | | | | | | | | |
| 1. | Beta mixture quantile normalization (BMIQ) | • This method targets smoothening the different biological characteristics (methylated and unmethylated regions) belonging to probe-I and probe-II.  • Also ensures the transformation in the probe II design should reduce the technical bias and the stabilized distribution of the beta values occurs throughout. | 2013 | wateRmelon | • It can introduce the normalization bias if the biological variation in the sample is not taken into account.  • It works on the illumina platform generated data | Comparatively better than SQN and SWAN methods | 773 | PMID: 27334613 |
| 2. | Quantile normalization | • The intensity matrix is replaced by the mean of the features assigned from the same quantile of distribution in array.  • The distribution is adjusted according to the reference sample. | 2013 | minfi, Limma, affy, sva, preprocessCore, wateRmelon, IMA | • Assumption: The raw data distribution should be similar across all samples. | Several extended versions of quantile normalization, shows better performance. | 841 | PMID: 12538238, 21839163, 23990268, 27334613, 28035024. |
| 3. | Lumi smooth quantile normalization | • Lumi smooth quantile normalization is a variant of quantile normalization.  • It uses the smoothing function to adjust for differences in the distribution of the data between samples and does the colour biased adjustment. | 2007 | Lumi Bioconductor package | • Quite sensitive to the outliners resulting due to probe saturation, or biological factors such as extreme gene expression levels | Better than the methods of SWAN, BMIQ, and ComBat | 1267 | PMID: 21839163, 23990268, 27334613, 18467348. |
| 4. | Peak based correction | • Peak-based correction method uses the gaussian mixture model.  • This method shows the non-overlapping windows of equal size and computing the average methylation level of CpG sites within each window. | 2020 | MethylSeekR, methylKit, Bsmooth, MGMIN | • The dilation transformation is based on the M values instead of beta values. | Comparatively better than BMIQ | 298 | PMID: 33193611, 22126295, 29671397. |
| 5. | Noob (Normal-exponential convolution using out-of-band probe) | • This method is used for Background correction and dye- bias adjustment on raw intensities which eliminates the use of reference sample.  • It models the distribution of background signal using the intensity values of the unmethylated probes. | 2016 | Minfi, MADA pipeline, Methylumi. | • It is sensitive to the occurrence of batch effects and shows limitation in handling the non-linear relationship of the probe’s samples. | ssNoob perform better than functional normalization and quantile normalization | 502 | PMID: 27334613, 28035024, 29671397. |
| 6. | SQN: Subset-quantile normalization | • SQN normalization is the method using the SWAN normalization method making use of the quantile-normalized values from the unmethylated probe subset to estimate the relationship between the intensities of the methylated and unmethylated probes for each CpG site.  • This relationship estimates the DNA methylation beta values for each CpG site. | 2010 | Minfi, WateRmelon, ChAMP, Enmix | • The dependency on the reference samples is not always representable for the analysis of the samples. | Comparatively, it is better than quantile and leoss normalization algorithm | 26 | PMID: 20976876, 28035024. |
| 7. | regRCPqn: (regional RCP with quantile normalization | • This algorithm is used for the data harmonization as it reduces the variability among the sample and increases the similarity datasets probes. | 2020 | regRCPqn package | • It cannot be used for the studies showing global differences such as cancer control studies.  • It cannot handle the gender bias variations in the samples. | Comparatively, it is better than Quantile normalization | 5 | PMID: 27334613 |
| 8. | Illumina: (genome studio) | • It calculates the average beta values based upon the fluorescent signals of the sample.  • These values determine the difference in the expression between the samples.  • It does quality control, Background correction and Normalization using bead array, quantile and LOESS methods. | 2000 | Software present in default illumina platform | • It is only compatible with the data generated by using illumina platform | Useful option for data generated from illumina platforms | 8 | PMID: 27605185 |
| Between array normalization | | | | | | | | |
| 9. | Stratified Quantile normalization | • This method can also be used for between-array normalization by normalizing the distribution of probe intensities across different arrays to a common distribution. | 2008 | Lumi, Minfi, Limma, Beadarray, preprocessCore | • It cannot handle the non-linear relationship between the expression probes. | Comparatively, its shows robust behaviour from Quantile Normalization and Loess Normalization. | 1728 | PMID: 24478339 |
| 10. | Dasen (Diagonal Adjustment for Significance Enhancement) | • The dasen method adjust the raw intensities of the sample, doing the background correction and applies standard quantile normalization oriented with methylated and unmethylated intensities of separate probes. | 2013 | Similarity shown with danes, danet, danen, daten1, daten2 preprocessing method | • This method is sensitive to outliners and batch effects.  • Suitable for the less sample processing and assumes no technical variations | Better performance than SWAN, SQN, BMIQ, ComBat, RUV | 504 | PMID: 28605401, 27334613, 12538238, 23631413 |
| 11. | Functional normalization (funNorm) | • This method removes the unwanted variation in the samples using the quantile normalization based on the reference probes and even reduces the false differential rate of the methylation expression.  • It reverts the unrequired variation possessed by the control probes. | 2014 | minfi | • The removal of the variation can result in the loss of biological signals.  • Mostly applicable for the gene expression data. | Functional normalization shows better robust behaviour as compared to ComBat, SVA, and RUV, on our assessment data sets. | 403 | PMID: 25599564, 28035024 |
| 12. | adjustedDasen | • It is majorly used for nullifying the variance arising in the sex chromosomes linked to CpG sites.  • It is a modified version of the Dasen preprocessing method. | 2022 | minfi and wateRmelon package | • Mostly effective for methylation analysis. | Better performance than the funNorm or dasen, and quantile normalization method | 2 | PMID: 35771651, 28035024 |
| 13. | SWAN: Subset-quantile normalization | • The methylated and unmethylated intensities of the probe is processed with the normal quantile normalization.  • The estimated relationship is then used to adjust the intensities of the unmethylated probes in the distributed subset probes. | 2012 | Minfi, WateRmelon, Rnbeads | • Not applicable to the small sized samples and can introduce batch effects for downstream analysis. | Better performance than the classic Quantile normalization | 554 | PMID: 22703947, 28035024 |
| 14. | Supervised normalization of microarrays | • It addresses the biological variation, remove the effects of adjustment variables and technical bias of the samples. | 2010 | SNM package | • The application requires all biological variations and study-specific adjustment variables of interest. | Better performance than the invariant set normalization (ISN) and quantile normalization | 68 | PMID: 20363728 |
| 15. | Lowess algorithm | • LOWESS helps in deriving the association between binary responses and continuous predictor factors using logistic regression.  • Prior to logistic regression, the systematic trends of the independent variables are removed. | 2004 | Stats R package, statsmodels (python package) and "LOESS" procedure in SAS | • It can produce bias results  • It can be sensitive to parameters | Better performance than the quantile normalization and beta-mixture quantile normalization | 96 | PMID: 12704611, 22771920, 15588297. |

Supplementary Table III: The comprehensive table giving details of normalization methods used for DNA methylation datasets and its respective details.
