# Supplementary material for: Methods in DNA methylation array dataset analysis: A review
Source: Comput Struct Biotechnol J. 2024 May 17;23:2304–25. doi: 10.1016/j.csbj.2024.05.015 (PMC11153885; doi:10.1016/j.csbj.2024.05.015)
Supplement: Supplementary file 4 — Supplementary material [file mmc4.docx]

| S.no. | Package/algorithm | Role/functions of the package | Pros | Cons | Reference |
| --- | --- | --- | --- | --- | --- |
| 1. | Minfi | PreprocessRaw:   - The method converts RGChannelSet (raw data) into MethylSet, containing methylation and unmethylation signals for each probe on the array. | It is appropriate for examination of normal tissue comparisons and epigenome-wide association studies that do not anticipate significant methylation alterations. | No normalization is performed.  This does not provide any quality control or filtering function. | [1] |
|  |  | Preprocessillumina:   - Convert an RGChannelSet to a MethylSet by implementing the preprocessing choices of background subtraction and control normalization. | The preprocessing carried out is similar to standard software (Genome Studio). | The need to manually select a reference array for control normalization could introduce variability if not chosen carefully. | [1,2] |
|  |  | PreprocessSWAN:   - It is a within-array normalization (SWAN), which adjusts for the differences between two types of probes on the array, which have different methylation levels. Applies quantile normalization in the probes with similar CpG content. | It reduces the technical variation and improves the correlation of methylation values across samples.  It can reduce the false positive values and increase the statistical power. | The alternation of the beta values distribution can lead to distortion in biological variance.  Can act slowly for large datasets. | [1] |
|  |  | PreprocessQuantile:   - The normalization of the methylated and unmethylated probes happens separately.  The method also considers the DNA variation by grouping the probes based on their region and applying quantile normalization to equalize the distribution of type I and type II signals. | Widely used for reducing technical variation. | It can introduce unwanted variation, and distortion in the biological variations and removes samples with low signal quality. | [1] |
|  |  | PreprocessNoob:   - This function performs noob background subtraction and dye-bias normalization. - Estimates and removes background noise from out-of-band probes. Also estimates the - dye bias from control probes and corrects it. | It can be applied to both 450k and EPIC arrays, as well as to combine data from different arrays. | The amount of background subtraction can vary depending on the offset parameter selection. | [1],[3] |
|  |  | PreprocessFunNorm:   - Uses control probes to remove between-array technical variation. - Applies preprocessNoob for background subtraction and infers the unwanted variation using the two principal control probe components. | Suitable for studies with large-scale differences, such as cancer/normal or between-tissue. | The requirement of a large number of control probes to estimate principal components, may or may not be available.  Variations derived from biological heterogeneity, sample quality, or batch effects are not taken into consideration. | [1], [4] |
| 2. | ANOVA | - Filtering out CpG sites with no significant difference in beta values among tissues using one-way ANOVA (p > 0.01). | They quantified how well these normalization methods minimized the impact of batch effects.  This was done by using ANOVA to examine the influence of batch on the first principal component. | ANOVA is sensitive to outliers.  This test assumes a normal distribution of the residuals and similar variation in each group. | [5],[6] |
| 3. | Tukey’s HSD test | - Filtering out CpG sites with small differences in mean beta values among tissues using Tukey’s HSD test (max difference < 0.15). - The TukeyHSD() function is available in base R | This is a multiple comparison method that controls family-wise error rate. | This test is generally conducted after ANOVA and also assumes the normal distribution of the data.  This test can be computationally intensive for the comparison of multiple groups. | [7] |
| 4. | ENmix | Imputation:   - Remaining missing values were imputed using the ENmix R package.   Filtering:   - The removal of unwanted experimental noise can enhance the accuracy of methylation measures.   Quality Control with ENmix:   - ENmix was used to discard outliers and replace missing values using the k nearest neighbor (KNN) algorithm. | ENmix outperformed other background correction methods in terms of reproducibility and accuracy and also minimizes data variation.  This preprocessing pipeline outperformed already existing pipelines ((ChAMP, Illumina, SWAN, FunNorm, Noob, wateRmelon, and RnBeads). | The limitation is again the requirement of the normalized data. | [8] |
| 5. | Impute package | KNN-imputation:   - The package uses the k-nearest neighbors (knn) method, which finds the most similar probes and uses their values to estimate the missing ones. | It can handle missing data in numerical datasets with different missing scenarios and rates, including data missing not at random (MNAR) and data missing completely at random (MCAR). | It may create artifacts and yield poor imputation results in the presence of multimodal or complex distributions. | [9][10] |
|  |  | Mean imputation:   - This method allocates the value of the missing CpG marker with that of the simple average of CpG markers. | This method does not require any complex modeling or assumption of the data. | Mean imputation may alter the inherent distribution and inter-variable relationships within the dataset, as it does not consider the variables’ intrinsic architecture. |  |
|  |  | Iterative imputation:   - Imputation of missing CpG markers is conducted through a model that iteratively predicts the missing values based on the values of other markers. | It can reduce the bias and increase the efficiency of the statistical analyses that use the imputed data. | It can be sensitive to the choice of the imputation models, the number of iterations, and the convergence criteria. |  |
| 9. | SVA | - Batch correction is performed using the ComBat function. - This is performed using the sva R package. - Removes the slide variables. | It can improve the accuracy and reproducibility of differential methylation analysis by reducing dependence and stabilizing error rate estimates. | Can be slow for large-scale data and can be sensitive to the choice of the number of surrogate variables, the method of estimation, and the model of association. | [11] |
| 10. | SVD | - The singular value decomposition (SVD) function is used for quality control. - This approach gives the data a linear representation in terms of a limited number of components that highlight the most notable variation patterns. | can be implemented the singular values and singular vectors obtained from SVD can provide insight into the structure of the data and the relationship between features. | Assumes a non-linear relationship between the variables and is quite sensitive to the scale of features. | [12] |
| 12. | Rnbeads | - Filtering of the probes before and after the normalization steps can be done by the Rnbeads pipeline. - This can filter the probe sites and samples according to a variety of user-set criteria. | With the availability of a sufficient number of close data points, KNN imputation gains satisfactory estimates of the missing values.  It has been used extensively for gene expression microarray data, and DNA methylation data. | The application of the KNN imputation fails due to the high number of missing values in the samples. Instead, the implementation of mean and median imputation approaches is considered effective. | [13] |
| 13. | IMA package | - This package can eliminate the problematic probes and samples with poor detection p values. | This package can Remove loci with missing β value, from the X chromosome, or with median detection P>0.05.  Also, remove loci with probes containing SNP(s) at/near the targeted CpG site. | The default setting of IMA is that no normalization will be performed, but can provide quantile normalization as an option. This cannot overcome the unwanted technical variation across samples. | [14] |
| 15. | Greedycut algorithm | - Can be implemented with the Rnbeads pipeline (majorly used for preprocessing and processing pipeline for DNA methylation array data). | This algorithm evaluates and eliminates the probes and samples with high impurity.  This can be done by  in the detection p-value table that contains the largest fraction of unreliable measurements; p < 0.05; for each sample. | It may not account for other sources of variation or noise in the data, such as probe design, probe type, or probe sequence. | [13,15] |
| 16. | Multiple Testing Correction: | - The most widely and simple method studied for multiple testing correction is the Benjamini-Hochberg False Discovery Rate (FDR) correction.      - Multiple testing adjustment corrects the possibility of finding false positives simply due to the number of tests performed | This procedure provides a strong control for FDR/pFDR at level α (for independent and positively correlated test statistics). | We corrected for multiple testing using Benjamini-Hochberg False Discovery Rate (FDR) correction, with the set significant threshold (adjusted p-value q < 0.05). | [16]; [17] |

**Supplementary Table IV: This table represents the data giving the advantages and limitations of the most selected preprocessing algorithms/steps and their respective packages/function in use.**
