# Supplementary material for: Methods in DNA methylation array dataset analysis: A review
Source: Comput Struct Biotechnol J. 2024 May 17;23:2304–25. doi: 10.1016/j.csbj.2024.05.015 (PMC11153885; doi:10.1016/j.csbj.2024.05.015)
Supplement: Supplementary file 5 — Supplementary material [file mmc5.docx]

| S.no. | R/Python package | Language | Available Platform | Usage in preprocessing | Usage in differential analysis/ annotation/visualization | Reference |
| --- | --- | --- | --- | --- | --- | --- |
|  | IMA | R language | <http://www.rforge.net/IMA> | - Package used for pre-processing of the data. - IMA default settings involve filtering loci with missing β values, X chromosome origin, median detection p-value > 0.05, and SNP-containing probes. Availability of sample quality control and logit transformation options followed by quantile normalization. | - Also, perform differential methylation analysis and annotation. | PMID: 22253290 |
|  | Lumi | R language | <https://www.bioconductor.org/packages/release/bioc/html/lumi.html> | - Used for preprocessing steps such as data input, quality control, variance stabilization, and normalization methods. | - Includes the processing methods previously designed for Affymetrix data. - Involved in gene annotation and performs variance stabilization of data. | PMID: 18467348 |
|  | Minfi | R language | <https://bioconductor.org/packages/release/bioc/html/minfi.html> | - Used for pre-processing, quality assessment, and detection of differentially methylated regions. - The function cpgCollapse () identifies the block of differential methylation. | - Can also be used for annotation of CpG sites with genomic features and visualization of data. | PMID: 24478339 |
|  | wateRmelon | R language | <https://bioconductor.org/packages/release/bioc/html/wateRmelon.html> | - Used for within array between array normalization methods such as type I/II bias corrections (BMIQ and SWAN) and Nasen respectively. | - The iDMR function is used for identifying differentially methylated regions (DMRs). | PMID: 23631413 |
|  | Methylumi | R language | <https://www.bioconductor.org/packages/release/bioc/html/methylumi.html> | - This package can be used for normalization, background correction, and quality control features for data derived from GoldenGate, Infinium, and Infinium HD platform arrays. | - Contains function for processing IDATs files from TCGA database. | PMID: 29671397 |
|  | COHCAP (City of Hope CpG Island Analysis Pipeline) | R language | <https://sourceforge.net/projects/cohcap/> | - Pre-processing and identification of differential methylated region of single-nucleotide resolution DNA methylation data. | - It offers integration with gene expression data. | PMID: 23598999 |
|  | FastDMA | R language | <http://bioinfo.au.tsinghua.edu.cn/software/fastdma/> | - Used for the identification of the single methylated probes and differentially methylated regions (DMRs). - Analysis of covariance (ANCOVA) interprets the analyzed results. | - Can be utilized for processing DNA methylation datasets sourced from the TCGA database. | PMID: 24040221 |
|  | NIMBL | R language | <https://cran.r-project.org/web/packages/nimble/index.html> | - Perform Quality Control and DMP calling. - Identifies DMR/DMS where the input contains the matrix of unmethylated and methylated average hybridization signal. | - NIMBL offers the capability to compare and visualize outcomes from multiple Infinium array analysis tools. | PMID: 22936948; 25233806 |
|  | SURVIVAL R package | R language | <https://cran.r-project.org/web/packages/survival/index.html> | - Used for survival analysis. - Cox regression analysis reveals gene methylation panel association with survival rate in univariate and multivariate models. | - Not used for identification of DMRs or DMPs. | PMID: 31547144 |
|  | DMRcate | R language | <https://bioconductor.org/packages/release/bioc/html/DMRcate.html> | - DMR calling. - The package utilizes linear model fit and empirical Bayesian statistical approach, identifying and extracting differentially methylated regions. | - Also functions for visualization of DMRs and methylation patterns. | PMID: 34320181 |
|  | Bumphunter | R language | <https://www.bioconductor.org/packages/release/bioc/html/bumphunter.html> | - Identification of bumps in the genomic data. - Linear regression model finds differential methylation at CpG sites, with permutation tests determining its significance. | - Also, perform Statistical testing and Multiple testing correction. | PMID: 22422453 |
|  | Probe Lasso | R language | <https://bioconductor.org/packages/ChAMP/> | - Define DMR boundaries using the Bioconductor package ChAMP. - The correlation matrix is computed from normalized beta values and then uses Stouffer's method to compute a P-value. | - Using TCGA data, this algorithm can uncover potential roles of hypomethylated transcription factor binding motifs not detectable by a fixed-window approach. | PMID: 25461817 |
|  | Comb-p | python language | <https://github.com/brentp/combined-pvalues> | - Deals with BED files to compute auto-correlation, combine neighboring P-values, and compensate for false discovery. | - Also find the enrichment zones, and determine significance. | PMID: 22954632 |
|  | BatMeth2 | Python and R language | <https://github.com/Guolian-gLi-HZAU/BatMeth2> | - It improves the DNA methylation calling and is a basic alignment tool. - This aligns BS reads (bsseq data) with reference genome and calculates methylation levels, visualizes, and analyzes DNA methylation | - DiffMeth conducts differential analyses with automatically defined or predefined regions. | PMID: 30669962 |
|  | DMRcaller | R language | <https://bioconductor.org/packages/release/bioc/html/DMRcaller.html> | - It improves the DNA methylation calling and is a basic alignment tool. - The package accurately calculates methylation levels and plots the correlation between the methylation levels | - Also used for calling DMRs, merging DMRs, visualization, annotation, and analysis of DNA methylation data. | PMID: 29986099 |
|  | Scikit-learn package | Python language | <https://scikit-learn.org/stable/install.html> | - Robust library of machine learning. - It provides tools for statistical modeling and machine learning including classification, regression, clustering, and dimensionality reduction. | - Used for integrative analysis of DNA methylation data with gene expression data and - development of biomarkers. | PMID: 34682092 |
|  | CpGtools | Python language | <https://github.com/liguowang/cpgtools.> | - Analysis of the DNA methylation data from bisulphide and Illumina array. - Used for quality control. | - ‘CpG position modules’ analyze genomic positions of CpGs, linking them with other genomic and epigenomic features, and producing enriched DNA motif logos within specific genomic contexts. - ‘CpG signal modules’ analyze the DNA methylation beta values to classify the CpG sites. - ‘Differential CpG analysis modules’ use the statistical methods for identifying the differentially methylated CpGs | PMID: 31808791 |
|  | HMST-Seq-Analyzer | Python language | <https://hmst-seq.github.io/hmst/> | - Calling DMR region of whole-genome bisulfite sequencing (WGBS) and reduced representation bisulfite sequencing (RRBS) datasets. - This package conducts initial data quality checks. | - It is a robust method for comprehensive analysis of 5mC and 5hmC data, capable of identifying Differentially Methylated Regions (DMRs), providing annotations, and visualizing methylation status, | PMID: 33163148 |
|  | Methylprep | Python language | <https://github.com/FoxoTech/methylprep/blob/master/README.md> | - Preprocessing and quality check steps including type-I channel switch inference, NOOB, poobah for p-value filtering, a quality mask for unreliable probe exclusion, and nonlinear dye bias correction, followed by calculation of beta-value, m-value, or copy-number matrix. | - Processing the Illumina methylation array. - Part of the Methylsuite package that processes and analyzes methylation data, enabling quality control and analysis. | PMID: 33992829 |
|  | Methyl-analyzer | Python language | <http://github.com/epigenomics/methylmaps.> | - Analysis of the genome-wide methylation. - This package analyzes the genome-wide DNA methylation data produced by the methylation mapping analysis by paired-end sequencing (Methyl-MAPS) method. | - Filters the methylated/unmethylated fragments, estimates the probability of CpG methylation probability, and visualization of the methylation profiles. | PMID: 21685051 |
| 1. C | gprofiler2 | R language | <https://cran.r-project.org/web/packages/gprofiler2/vignettes/gprofiler2.html> | - This package initiates the gene list functional profiling and incorporates functional enrichment analysis. - These enrichment results are also visualized for better interpretation. | - This package is not involved in identifying DMRs/DMPs but can also convert gene/protein/SNP identifiers and map orthologous genes across species. | PMID: 33564394 |
|  | IlluminaHumanMethylationEPICanno.ilm10b4.hg19 | Bioconductor R package | <https://www.bioconductor.org/packages/release/data/annotation/html/IlluminaHumanMethylationEPICanno.ilm10b4.hg19.html> | - The EPIC array CpG loci were annotated using this package. Overlap is based on genomic mappings from the annotation package. | - This package is not involved in identifying DMRs/DMPs but can also annotate SNP from various versions of dbSNP. | PMID: 27347385 |
|  | Rnbeads | R language | <http://bioconductor.org/packages/release/bioc/html/RnBeads.html> | - RnBeads supports various assays including Infinium and EPIC microarrays, bisulfite sequencing, MeDIP-seq, and MBD-seq data. - It incorporates advanced normalization Quality control for detecting sample outliers and filtration. - The package allows for the identification of batch effects and phenotype covariates based on sample annotations. | - Finding the differentially methylated genes (DMGs) from a large sample size. | PMID: 30871603 |
|  | ComplexHeatmap | R language | <https://www.bioconductor.org/packages/release/bioc/html/ComplexHeatmap.html> | - Majorly used for data visualization purposes. - ComplexHeatmap package visualizes the association between datasets and potential patterns in Differentially Methylated Genes (DMGs). | - This package is not involved in identifying DMRs/DMPs. | PMID: 27207943 |
|  | MethylMix | R language | <https://www.bioconductor.org/packages/release/bioc/html/MethylMix.html> | - It analyzes the functional methylation changes that affect the gene expression levels. - Preprocessing involves removing samples and genes with excessive missing values, filling in missing data, and performing batch correction. | - The identification of the MDG (methylation-driven genes) was done with the use of the METHYLMIX R package, supporting the integrative analysis of gene expression and DNA methylation data. | PMID: 29668835 |
|  | ChAMP (Chip Analysis Methylation Pipeline) | R language | <https://bioconductor.org/packages/ChAMP/> | - Analysis of the DNA methylation 450K array. - Package identifies copy number alterations and implements several quality control and normalization methods. | - This pipeline can also identify differentially methylated regions by using Probe Lasso, Bumphunter, and DMRcate algorithms. | PMID: 24336642 |
|  | MethylAid | R language | [www.bioconductor.org/packages/3.0/bioc/html/MethylAid.html](file:///C:\Users\karishma\OneDrive\Desktop\DNA%20methylation%20review\uploading%20review%20folder\Computational%20and%20Structural%20Biotechnology%20Journal\www.bioconductor.org\packages\3.0\bioc\html\MethylAid.html) | - Access to quality control. - Packages enable an interactive user interface for quality control of genome-wide DNA methylation using RStudio's shiny package. | - Summarize and visualize the function calls. - Not involved in identifying DMRs/DMPs. | PMID: 25147358 |
|  | Illumina450ProbeVariants.db | R language | <https://bioconductor.org/packages/Illumina450ProbeVariants.db/> | - Used as an Annotation package. - This is an annotation package for Illumina human methylation 450K data which is a collection of the variant’s information belonging to four different populations (Asian, American, African, and European) | - Not involved in identifying DMRs/DMPs. - This also provides a database that links probe identifiers from the array to genetic variants such as single nucleotide polymorphisms (SNPs) and insertions/deletions (indels). | PMID: 25233806 |
|  | shinyMethyl | R language | <http://www.bioconductor.org/shinyMethyl> | - The interactive visualization tools for the Illumina methylation data array. - This package is based on the minfi and shiny used for quality assessment and analysis of the effect of pre-processing of the 450K methylation array. | - ShinyMethyl condenses 450k experiments into compact, exportable R objects, facilitating interactive exploration via a user-friendly interface. - It enables the exploration of phenotypic associations by incorporating features like coloring and principal component analysis within its interactive environment. | PMID: 25285208 |
|  | MethRaFo | Python language | <https://github.com/phoenixding/methrafo.> | - Estimation of the MeDIP-seq methylation with the use of random forest Regressor. - This package involves computing RPKM from bam files (input), representing normalized read counts per nucleotide. Subsequently, local CpG density is determined for a specified window size. | - This decreases the runtime of the estimation of the genome-wide methylation and increases the accuracy of the task. | PMID: 29036558 |
|  | DMRfinder | Python and R language | <https://github.com/jsh58/DMRfinder> | - Identify the DMR region from high-throughput bisulfite sequencing datasets. - Pipeline for alignment and extracting the methylation count followed by performing single-linkage clustering of the methylation sites (CpG sites) into the genomic regions. | - Afterwards, it contrasts methylation levels utilizing beta-binomial hierarchical modeling and Wald tests. - It also includes the examination of new methylation sites and methylation linkage, along with simultaneous statistical analysis of multiple sample groups. | PMID: 29187143 |
|  | GLINT | Python language | <https://github.com/cozygene/glint/releases> | - GLINT focuses solely on analyzing pre-processed methylation data. - It maintains quality control, adjusting for tissue heterogeneity, inferring population structure, methylation imputation, association testing, and Visualization. | - Identification of methylation in Illumina methylation array data. | PMID: 28177067 |
|  | CpGassoc | R language | <https://cran.r-project.org/web/packages/CpGassoc/index.html> | - CpGassoc analyzes DNA methylation data using quality control checks, permutation tests, and quality plots, examining methylation relationships and individual phenotypes. | - The generation of Q-Q plots, scatterplots, boxplots, and Manhattan plots enables the visualization of significant CpG sites' deviation from the expected null distribution and evaluation of genomic inflation. | PMID: 22451269 |

**Supplementary Material V: The list of packages and their uses majorly selected in the workflows of DNA methylation analysis**

References

[213]Liu D., Zhao L., Wang Z., Zhou X., Fan X., Li Y., et al. EWASdb: Epigenome-wide association study database. Nucleic Acids Res 2019;47:D989–D993. doi:10.1093/nar/gky942.

[214]Satterlee J.S., Chadwick L.H., Tyson F.L., Mcallister K., Beaver J., Birnbaum L., et al. The NIH Common Fund/Roadmap Epigenomics Program: Successes of a comprehensive consortium. 2019.

[215]Bujold D., Morais D.A. de L., Gauthier C., Côté C., Caron M., Kwan T., et al. The international human epigenome consortium data portal. Cell Syst 2016;3:496–499.e2. doi:10.1016/j.cels.2016.10.019.

[216]Komaki S., Shiwa Y., Furukawa R., Hachiya T., Ohmomo H., Otomo R., et al. IMETHYL: An integrative database of human DNA methylation, gene expression, and genomic variation. Hum Genome Var 2018;5. doi:10.1038/hgv.2018.8.

[217]Cantelli G., Bateman A., Brooksbank C., Petrov A.I., Malik-Sheriff R.S., Ide-Smith M., et al. The European Bioinformatics Institute (EMBL-EBI) in 2021. Nucleic Acids Res 2022;50:D11–D19. doi:10.1093/nar/gkab1127.

[218]Li M., Zou D., Li Z., Gao R., Sang J., Zhang Y., et al. EWAS Atlas: a curated knowledgebase of epigenome-wide association studies. Nucleic Acids Res 2019;47:D983–D988. doi:10.1093/nar/gky1027.

[219]Fernández J.M., de la Torre V., Richardson D., Royo R., Puiggròs M., Moncunill V., et al. The BLUEPRINT data analysis portal. Cell Syst 2016;3:491–495.e5. doi:10.1016/j.cels.2016.10.021.

[220]The German epigenome programme (DEEP) n.d.

[221]Allelic Epigenome Project n.d.

[222]Fetal brain meQTLs n.d.

[223]Li D., Purushotham D., Harrison J.K., Hsu S., Zhuo X., Fan C., et al. WashU epigenome browser update 2022. Nucleic Acids Res 2022;50:W774–W781. doi:10.1093/nar/gkac238.

[224]Tryka K.A., Hao L., Sturcke A., Jin Y., Wang Z.Y., Ziyabari L., et al. NCBI’s database of genotypes and phenotypes: DbGaP. Nucleic Acids Res 2014;42. doi:10.1093/nar/gkt1211.

[225]Deng G., Yang J., Zhang Q., Xiao Z.X., Cai H. MethCNA: a database for integrating genomic and epigenomic data in human cancer. BMC Genom 2018;19. doi:10.1186/s12864-018-4525-0.

[226]Cerami E., Gao J., Dogrusoz U., Gross B.E., Sumer S.O., Aksoy B.A., et al. The cBio Cancer Genomics Portal: an open platform for exploring multidimensional cancer genomics data. Cancer Discov 2012;2:401–404. doi:10.1158/2159-8290.CD-12-0095.

[227]Ding W., Chen J., Feng G., Chen G., Wu J., Guo Y., et al. DNMIVD: DNA methylation interactive visualization database. Nucleic Acids Res 2020;48:D856–D862. doi:10.1093/nar/gkz830.

[228]Pan-cancer analysis of whole genomes. Nature 2020;578:82–93. doi:10.1038/s41586-020-1969-6.

[229]Tate J.G., Bamford S., Jubb H.C., Sondka Z., Beare D.M., Bindal N., et al. COSMIC: the catalogue of somatic mutations in cancer. Nucleic Acids Res 2019;47:D941–D947. doi:10.1093/nar/gky1015.

[230]Gong J., Wan H., Mei S., Ruan H., Zhang Z., Liu C., et al. Pancan-meQTL: a database to systematically evaluate the effects of genetic variants on methylation in human cancer. Nucleic Acids Res 2019;47:D1066–D1072. doi:10.1093/nar/gky814.

[231]rdocumentation n.d.

[232]Analysis of 450k data using minfi n.d.

[233]Drummond G.B., Vowler S.L., Gordon D., Drummond B. Analysis of variance: variably complex Gordon Drummond is Senior Statistics Editor for The Journal of Physiology. Key points. Adv Physiol Educ, Microcirc, Clin Exp Pharmacol Physiol 2012. doi:10.1111/(ISSN)1476-5381/homepage/statistical_reporting.htm.

[234]Shiah Y.J., Fraser M., Bristow R.G., Boutros P.C. Comparison of pre-processing methods for Infinium HumanMethylation450 BeadChip array. Bioinformatics 2017;33:3151–3157. doi:10.1093/bioinformatics/btx372.

[235]Jose Claudio Faria A., Bezerra Allaman I., Ivan Bezerra Allaman M. Package “TukeyC.” 2023.

[236]Xu Z., Niu L., Taylor J.A. The ENmix DNA methylation analysis pipeline for Illumina BeadChip and comparisons with seven other preprocessing pipelines. Clin Epigenetics 2021;13. doi:10.1186/s13148-021-01207-1.

[237]Lena P.Di, Sala C., Prodi A., Nardini C. Methylation data imputation performances under different representations and missingness patterns. BMC Bioinforma 2020;21. doi:10.1186/s12859-020-03592-5.

[238]Troyanskaya O., Cantor M., Sherlock G., Brown P., Hastie T., Tibshirani R., et al. Missing value estimation methods for DNA microarrays 2001;vol. 17.

[239]Package “sva” Title Surrog Var Anal 2024.

[240]Korobeynikov A., Larsen R.M., Berkeley L., Laboratory N.-T., Maintainer]. Package “svd” Title Interfaces to Various State-of-Art SVD and Eigensolvers. 2023.

[241]Müller F., Scherer M., Assenov Y., Lutsik P., Walter J., Lengauer T., et al. RnBeads 2.0: Comprehensive analysis of DNA methylation data. Genome Biol 2019;20. doi:10.1186/s13059-019-1664-9.

[242]Wang D., Yan L., Hu Q., Sucheston L.E., Higgins M.J., Ambrosone C.B., et al. IMA: An R package for high-throughput analysis of Illumina’s 450K Infinium methylation data. Bioinformatics 2012;28:729–730. doi:10.1093/bioinformatics/bts013.

[243]Assenov Y., Müller F., Lutsik P., Walter J., Lengauer T., Bock C. Comprehensive analysis of DNA methylation data with RnBeads. Nat Methods 2014;11:1138–1140. doi:10.1038/nmeth.3115.

[244]Noble W.S. How does multiple testing correction work? Nat Biotechnol 2009;27:1135–1137. doi:10.1038/nbt1209-1135.

[245]Li D., Xie Z., Pape M., Le, Dye T. An evaluation of statistical methods for DNA methylation microarray data analysis. BMC Bioinforma 2015;16. doi:10.1186/s12859-015-0641-x.
